# Polymer–prodrug conjugates as candidates for degradable, long-acting implants, releasing the water-soluble nucleoside reverse-transcriptase inhibitor emtricitabine†

**DOI:** 10.1039/d3tb02268d

**Published:** 2023-11-07

**Authors:** Chung Liu, Faye Y. Hern, Anika Shakil, Kartik Temburnikar, Pierre Chambon, Neill Liptrott, Tom O. McDonald, Megan Neary, Charles Flexner, Andrew Owen, Caren Freel Meyers, Steve P. Rannard

**Affiliations:** a Department of Chemistry, University of Liverpool Crown Street Liverpool L69 7ZD UK srannard@liverpool.ac.uk; b Materials Innovation Factory, University of Liverpool Crown Street L69 7ZD UK; c Centre of Excellence in Long-acting Therapeutics (CELT), University of Liverpool Liverpool L7 3NY UK; d Department of Pharmacology and Molecular Sciences, The Johns Hopkins University School of Medicine 725 North Wolfe St. Baltimore MD 21205 USA; e Department of Pharmacology and Therapeutics, Institute of Systems, Molecular and Integrative Biology, University of Liverpool Liverpool L7 3NY UK

## Abstract

Circulating, soluble polymer–drug conjugates have been utilised for many years to aid the delivery of sensitive, poorly-soluble or cytotoxic drugs, prolong circulation times or minimise side effects. Long-acting therapeutics are increasing in their healthcare importance, with intramuscular and subcutaneous administration of liquid formulations being most common. Degradable implants also offer opportunities and the use of polymer–prodrug conjugates as implant materials has not been widely reported in this context. Here, the potential for polymer–prodrug conjugates of the water soluble nucleoside reverse transciption inhibitor emtricitabine (FTC) is studied. A novel diol monomer scaffold, allowing variation of prodrug substitution, has been used to form polyesters and polycarbonates by step-growth polymerisation. Materials have been screened for physical properties that enable implant formation, studied for drug release to provide mechanistic insights, and tunable prolonged release of FTC has been demonstrated over a period of at least two weeks under relevant physiological conditions.

## Abstract 2

Emtricitabine, has been modified to form a series of diol monomers for pendant polymer–prodrug conjugate synthesis. Screening has identified structures for implant formation able to release parent drug *via* enzymatic cleavage mechanisms.

## Graphical abstract

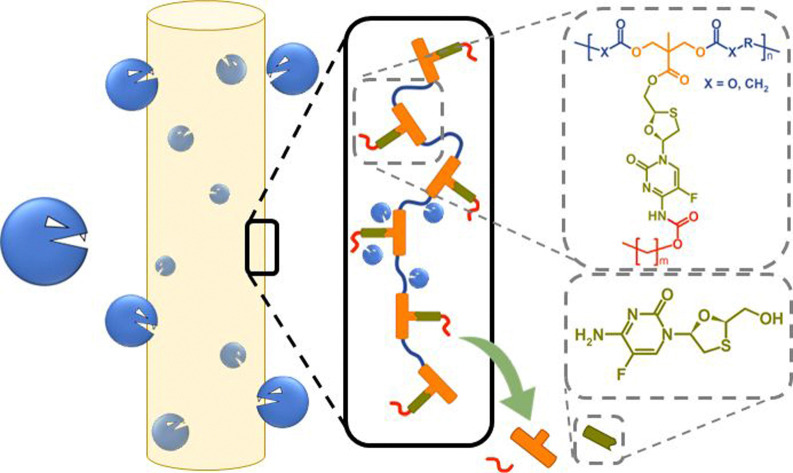

## Introduction

Controlling drug release to enable targeting of disease,^1^ prolong duration of drug circulation,^2^ or provide sustained exposure within a target therapeutic window for long periods^3^ has benefitted from polymer science for many years. Since 1955 demonstration of extended release of mescaline (mouse model) after subcutaneous dosing of the drug covalently bound *via* a cleavable linker to a poly(vinyl pyrrolidone-*stat*-acrylic acid) statistical copolymer,^4^ polymer–drug conjugates have been a significant area of research interest. Foundational research and development from groups led by Ringsdorf, Duncan, Kopecek and others,^5–7^ have taken some of the most toxic drug compounds through to human evaluation of anticancer benefits. Additionally, conjugation of drugs to poly(ethylene glycol), PEG, has spawned a range of PEG-ylated medicines with US Food and Drug Administration clinical approval stretching back to Adagen® in 1990.^8,9^ A host of new PEG-ylated therapies are undergoing development or progressing through clinical studies.

Cancer is a global research focus for polymer therapeutics and has received considerable attention. The growing interest in long-acting therapeutics, however, encompasses many diseases and conditions, including therapy and prevention/prophylaxis strategies.^10–12^ In short, long-acting therapeutics aim to provide efficacious circulating drug exposures for considerable timescales after a single administration; timescales that may stretch to weeks or several months. The benefits to patients are considerable, including minimising the impact of daily oral tablet dosing on lifestyle, but carers and clinicians are better able to ensure adherence to therapy with resulting improvements in efficacy and outcomes.^13–16^ Unmet healthcare needs span areas such as chronic infection, cardiovascular health, psychosis treatments and prevention of life-threatening diseases such as malaria, hepatitis and tuberculosis.^17^

**Fig. 1:**
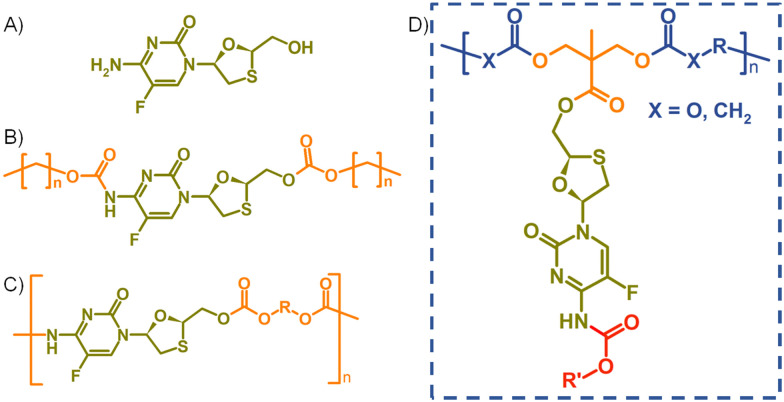
Structures of (A) emtricitabine (FTC), (B) symmetrical carbamate–carbonate small molecule prodrugs, (C) step-growth polymers containing FTC within the backbone, and (D) target pendant polymer–prodrug conjugates within this study.

Human immunodeficiency virus (HIV) infection requires a life-long commitment to daily dosing of a combination of antiviral drug compounds.^17^ The first long-acting HIV treatment, containing a rilpivirine/cabotegravir combination, Cabenuva®, has recently received regulatory approval in the UK, Canada, Europe and the US.^12^ Conventional oral HIV therapies utilise combinations of up to three ARVs including nucleoside reverse transcriptase inhibitors (NRTIs). NRTIs are widely considered as the “backbone” of oral HIV regimens and usually exhibit relatively high water-solubilities; for example, the reported water solubility of emtricitabine (FTC, Fig. 1(A)) is 112 mg mL^−1^ at 25 °C,^18^ and lamivudine is 140 mg mL^−1^ at 37 °C.^19^ Such values render NRTIs as poor candidates for conventional long-acting therapy approaches.

In recent years, strategies utilising poorly water-soluble NRTI prodrugs have opened new opportunities for long-acting product candidates, Fig. 1.^20,21^ Typically, these approaches have utilised the poorly water-soluble prodrug, Fig. 1(B), within nanoparticle or micelle formation to enable intramuscular injection, with prodrug activation at the injection site, or shortly after interaction with plasma, leading to sustained circulating parent drug concentrations. A polymeric prodrug approach has also been reported, where the amine and hydroxyl functional groups of FTC were utilised in a step-growth polymerisation to form a polymeric carbamate/carbonate structure containing monomeric FTC backbone repeat units, Fig. 1(C). The FTC-containing polymer–prodrug was shown to activate and release FTC parent drug during enzymatic degradation under physiological conditions.^20^ The use of FTC as a monomer in a step-growth polymerisation took inspiration from reports from Uhrich and coworkers who established opportunities using drugs such as morphine and aspirin as step-growth monomers.^22–25^ Here we report the design, synthesis and evaluation of polymer–prodrug conjugate implants containing FTC, Fig. 1(D) and their activation under physiological conditions.

## Results and discussion

### Design and synthesis of FTC-derived ester-carbamate prodrug monomers

FTC prodrugs and step-growth polymers, Fig. 1(B) and (C), were readily previously synthesised using mono- or bis-chloroformates with varying structures.^20,26^ The resulting carbonate and carbamate links were known enzymatic substrates and activation to parent FTC was expected to occur under appropriate conditions. The carbamate linking group has previously been utilised in the chemistry of capecitabine, a prodrug of 5-fluorouracil,^27^ and the 5-flucytosine ring is common to FTC. The cleavage of the pentyl carbamate of capecitabine has been studied clinically and is known to be highly susceptible to human carboxylesterases in the liver.^28^ To create a pendant polymer–drug conjugate using FTC, the carbamate group was maintained, predominantly as a route to modify the resulting polymer physical properties through substituent variation. The primary hydroxyl functionality present on the 1,3-oxathiolane ring was therefore targeted as a site for conjugation to the polymer backbone, with an ester considered to be an appropriate cleavable linking chemistry, Fig. 1(D). An ideal long-acting polymer–drug conjugate would fully degrade after administration and avoid the need to remove any non-drug components after drug release. As such, ester and carbonate polymer backbones were selected for evaluation, thus requiring the synthesis of an FTC-derived monomeric diol that could be used in a step-growth polymerisation.

Our previous reports of carbamate/carbonate FTC prodrugs, Fig. 1(B), involved the reaction of the amine and 5′-hydroxyl groups with various chloroformates to form the symmetrically substituted prodrugs; however, selective hydrolysis of the 5′-carbonate using LiOH allowed the comparative evaluation of the mono-carbamate structure.^20^ Ibrahim *et al.* have described the formation of the mono-palmitoyl ester of FTC using *t*-butyl magnesium chloride to generate the 5′ alkoxide at −78 °C and enable the selective reaction without protection of the primary amine functionality.^29^

**Scheme 1:**
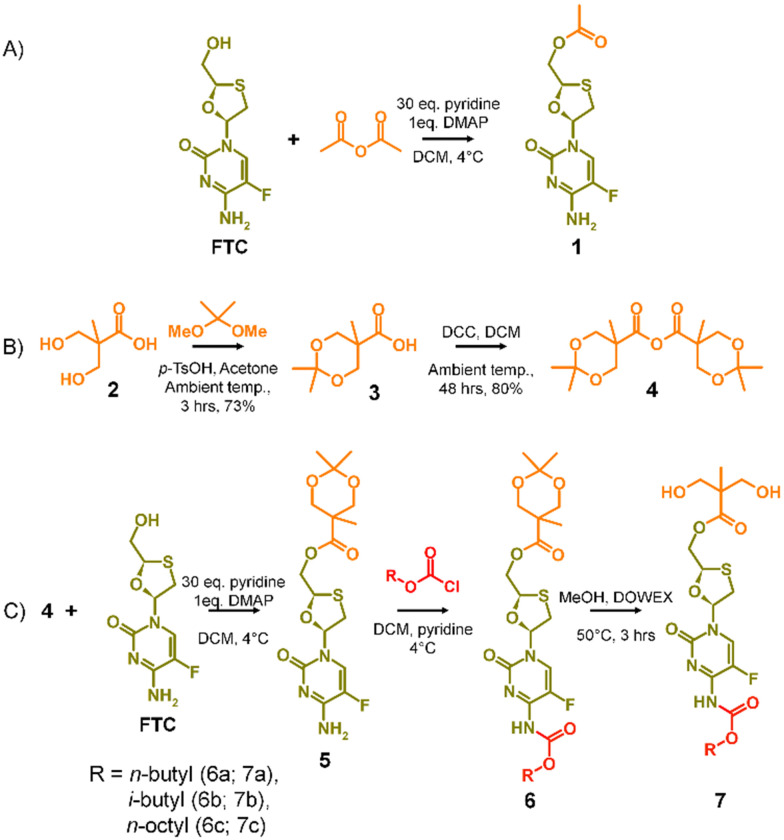
A_2_ diol monomer synthesis strategy (A) model study selectively forming mono-acetylated FTC, (B) formation of the acetonide protected 2,2-bis(hydroxymethyl)propionic acid anhydride, and (C) synthesis of carbamate protected diol monomers.

The potential for selective ester formation was therefore studied using an adaptation of the selective acetylation of the hydroxyl functionalities present on the deoxy-sugar ring of 2′,3′-diacetoxy-5′-deoxy-5-fluorocytidine during the synthesis of capecitabine.^30^ In summary, FTC, 4-dimethylaminopyridine (DMAP) and an excess of pyridine were dissolved in dichloromethane (DCM) at 4 °C followed by dropwise addition of acetic anhydride. After warming to room temperature and stirring overnight, the reaction was purified to give the mono-acetylated FTC product, 1, in a recovered yield of 44% based on FTC, Scheme 1(A).

The success of the synthesis of 1 led to a synthetic strategy for the formation of an FTC-derived A_2_ diol monomer that could be utilised in step-growth polymerisation to form the target polymer–prodrug conjugates, Scheme 1(B) and (C).

The use of 2,2-bis(hydroxymethyl)propionic acid, (BMPA) 2, has been reported many times in the synthesis of hyperbranched polymers, dendrimers and hyperbranched polydendrons amongst other polymeric materials.^31–36^ As in previous reports, the acetonide protection of BMPA was conducted to form 3 with subsequent dehydration to form the symmetric anhydride 4, Scheme 1(B). The reaction of 4 with FTC, under identical conditions used in the model synthesis of 1, provided the mono-ester of FTC, 5, without protection of the amine group, as seen in the formation of 1, and with conservation of the acetonide protection of the diol unit, Scheme 1(C). Access to 5 allowed the reaction of the available amine functionality with alkyl chloroformates (*n*-butyl, i-butyl, and *n*-octyl) to form a series of ester-carbamate structures, 6a–c, with subsequent removal of the acetonide protection using DOWEX® beads to yield the A_2_ monomers 7a–c, Scheme 1(C). The synthesis of 5 was optimised to allow scale up to approximately 500 g, providing adequate precursor for repeated synthesis of the three diol monomers and later polymerisations.

Each stage of the synthesis of the three FTC-derived diol A_2_ monomers was characterised using a combination of ^1^H and ^13^C nuclear magnetic spectroscopy (NMR), electrospray mass spectrometry and infra red spectroscopy. In conjunction with assignment of resonances within the NMR spectra, the success of each synthesis was confirmed by the presence of [M + H]^+^ and [M + Na]^+^ adducts within the mass sectrometry analysis (ESI,† Fig. S1–S36).

### Synthesis and characterisation of FTC-derived polymer–prodrug conjugates

**Scheme 2:**
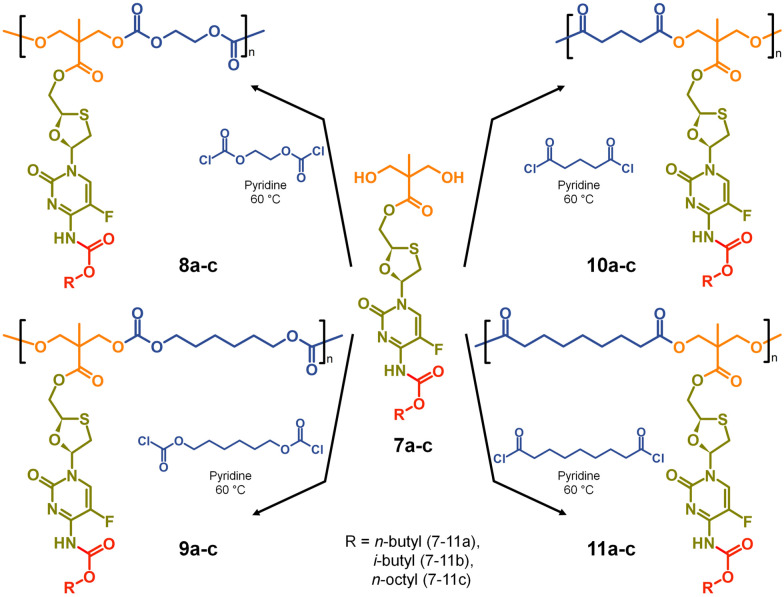
Synthesis of polycarbonate and polyester polymer–prodrug conjugates with structural diversity using three carbamate protected A_2_ diol monomers.

The formation of aliphatic polycarbonates was selected as the carbonate linking group is susceptible to cleavage within biological environments. Two commercially available bischloroformates, ethylene bischloroformate and hexamethylene bischloroformate, were chosen to act as B_2_ monomers within the step-growth polymerisation with 7a–c to add structural diversity to the resulting polymers. As such, polycarbonates 8a–c and 9a–c were synthesised through a 1 : 1 stoichiometry of the A_2_ and B_2_ monomers at 60 °C in pyridine, Scheme 2.

For comparison, polyester backbones were also synthesised utilising 7a–c and either glutaryl chloride or azelaic acid dichloride as the B_2_ monomers, forming 10a–c and 11a–c, Scheme 2. The diacid chlorides were selected to generate analogues of the polycarbonate structures with comparably short or long aliphatic links in the repeating units.

The twelve FTC-derived polymer–prodrug conjugates were characterised by ^1^H and ^13^C NMR, oligomer size exclusion chromatography (SEC) and differential scanning calorimetry (DSC) to investigate polymer structure, molecular weight, dispersity and glass transition temperature (*T*_g_). The thermal properties were of particular interest given the target of an implantable polymeric structure.

To enable ready identification of the polymer structures during the discussion, the following nomenclature will be utilised. Firstly, the structure number will be provided, followed by a description of the polymer backbone and, in parentheses, an indication of B_2_ monomer repeat and the pendant carbamate alkyl chain. For example, 8a polycarb(Et-*n*Bu) will represent the polycarbonate structure formed from the polymerisation of the ethylene bischloroformate and the FTC-derived diol containing an *n*-butyl carbamate modification, 7a.

**Fig. 2:**
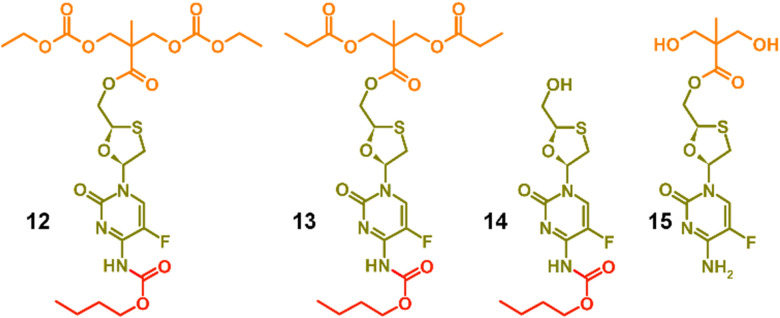
Model structures formed to enable characterisation and assignment of ^1^H and ^13^C nuclear magnetic resonance spectra.

To facilitate the assignment of NMR spectra and understanding of the behaviour of the polymers, four model compounds were synthesised; two that mimic the polymers 8a polycarb(Et-*n*Bu) and 10a polyester(Glu-*n*Bu), and two monosubstituted structures that represent possible intermediate degradation products. The synthesis of the polymer mimics was readily achieved *via* the reaction of 7a with ethyl chloroformate or propionyl chloride to form the dicarbonate, 12, or diester, 13, structures respectively. The selective synthesis of the mono substituted butyl carbamate of FTC, 14, was synthesised by slow addition of butyl chloroformate to FTC in the presence of pyridine using a 1 : 1 : 1 molar ratio of the three reagents, whilst the monoester 15 was synthesised by removal of the acetonide protecting group from 5, Fig. 2 (ESI,† Fig. S37–S52).

**Fig. 3:**
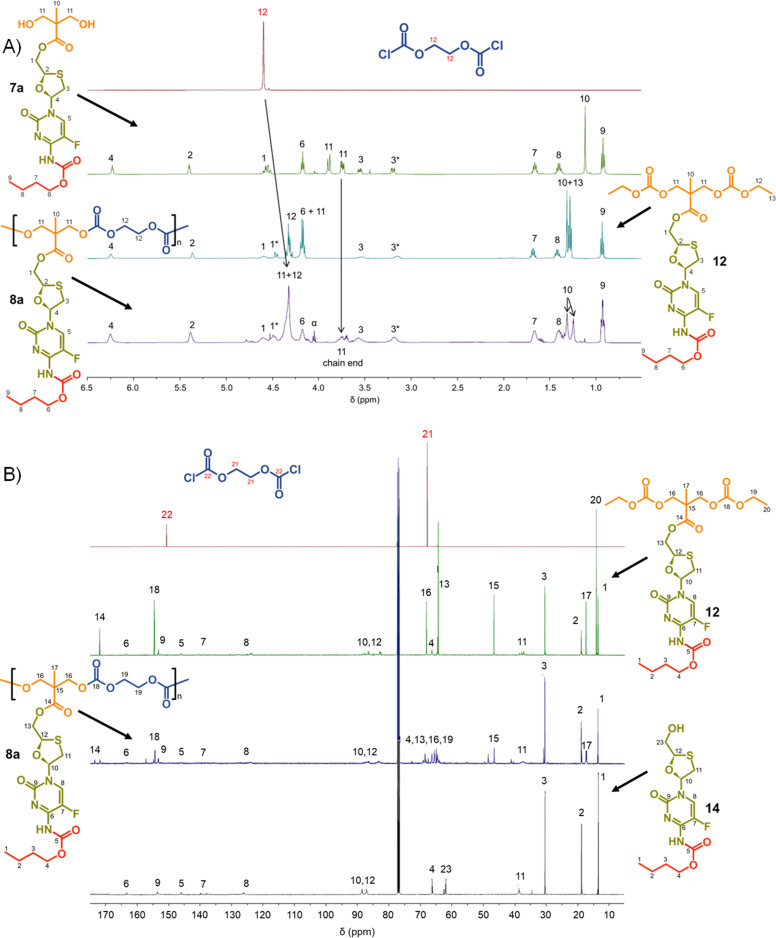
Nuclear magnetic resonance spectroscopy characterisation of polycarbonate polymer–prodrug conjugates (typical example): (A) ^1^H NMR spectra of monomers 7a and ethylene glycol bischloroformate, model compound 12 and polymer 8a, and (B) ^13^C NMR spectra of monomers ethylene glycol bischloroformate, model compounds 12 and 14, and polymer 8a.

Focussing first on the polycarbonate samples, a comparison of the ^1^H NMR spectra of 7a, 12 and 8a polycarb(Et-*n*Bu), Fig. 3, confirmed the successful polymerisation, with many resonances present within 12 also present within 8a polycarb(Et-*n*Bu). Complex splitting of the methylene protons adjacent to and within the oxathiolane sugar ring (H_1_ and H_3_, Fig. 3) was observed and is related to the conformation of the ring and restricted motion generating inequivalent environments in both locations. A broadening of the resonances within the polymer ^1^H spectrum is indicative of polymer formation, and a resonance at approximately 3.75 ppm was assigned as a hydroxymethyl chain end of the resulting polymer derived from the diol monomer 7a after spectra comparison with 12 (H_11_, Fig. 3(A)).

Similar ^13^C NMR comparisons using spectra obtained from 1, 12, 14, and 8a polycarb(Et-*n*Bu) also confirmed the formation of the polymeric backbone through reaction of ethylene bischloroformate with 7a (Fig. 3(B) and ESI,† Fig. S1–S4). Resonances at 154.4 ppm are indicative of carbonyls resulting from backbone carbonate formation and are clearly shown in the model 12 and 8a polycarb(Et-*n*Bu). 2D Heteronuclear single quantum coherence studies allowed the confident assignment of all resonances (ESI,† Fig. S53–S55). Similar syntheses and NMR analyses were conducted using 7b and 7c to form 8b polycarb(Et-iBu) and 8c polycarb(Et-*n*Oct). Additionally, 9a polycarb(Hex-*n*Bu), 9b polycarb(Hex-iBu) and 9c polycarb(Hex-*n*Oct) were also synthesised using hexamethylene bischloroformate, Table 1 (ESI,† Fig. S56–S65).

**Table 1:** Oligomer size exclusion chromatography (SEC) and differential scanning calorimetry (DSC) characterisation of polycarbonate polymer–prodrug conjugates with varying carbamate pendant group and backbone bischloroformate monomer residues

Polymer	SEC			DSC
	*M*_n_ (g mol^−1^)	*M*_w_ (g mol^−1^)	*Đ*	*T*_g_ (°C)
8a polycarb(Et-*n*Bu)	4460	9785	2.19	33
8b polycarb(Et-iBu)	3790	6900	1.82	37
8c polycarb(Et-*n*Oct)	3200	4900	1.53	19

9a polycarb(Hex-*n*Bu)	4230	6020	1.42	11
9b polycarb(Hex-iBu)	4455	6715	1.51	15
9c polycarb(Hex-*n*Oct)	3940	5750	1.46	−6

**Fig. 4:**
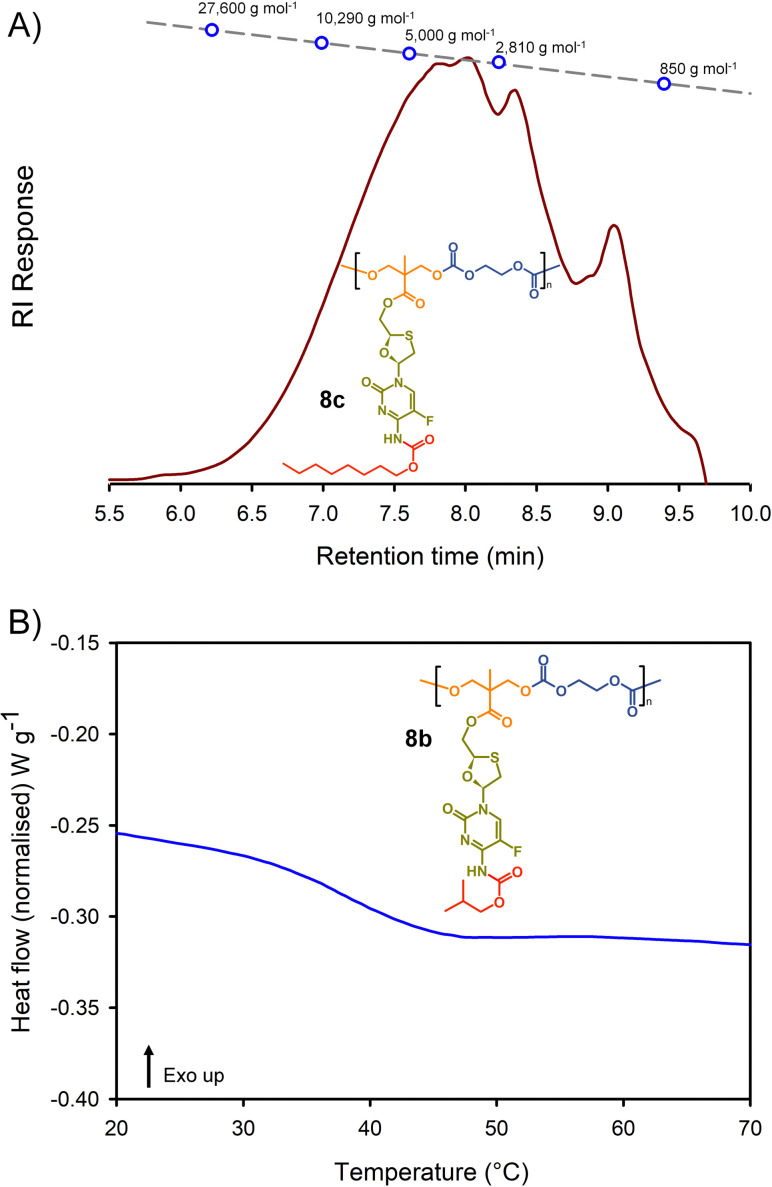
Example characterisation of polycarbonate polymer–prodrug conjugates: (A) oligomer size exclusion chromatography (SEC) of polycarbonate 8c, and (B) differential scanning calorimetry (DSC) characterisation of polycarbonate 8b.

Molecular weight analysis was complicated by the poor solubility of the polymers within commonly-used SEC solvents, therefore, oligomer SEC analysis using DMF as eluent was conducted against a calibration created from a series of poly(methyl methacrylate), *p*(MMA), standards (850–27 600 g mol^−1^) using a single detection method (refractive index) (Fig. 4(A)). The values obtained are, therefore, *p*(MMA) equivalent molecular weights and may not fully represent the true chain lengths of the prodrug-containing polymers due to restricted coiling of these polymers in comparison to *p*(MMA). Additionally, the exclusion limit of the oligomer columns was 30 000 g mol^−1^ and a clear cut-off is seen within some of the chromatograms at molecular weights above the calibration series. That said, the oligomer SEC was clearly able to determine several species within the molecular weight distributions of the polymers as expected from an A_2_ + B_2_ step-growth reaction using the diols 7a–c and two bischloroformates.

The *p*(MMA) equivalent number average (*M*_n_) and weight average (*M*_w_) molecular weights for 8a polycarb(Et-*n*Bu), 8b polycarb(Et-iBu) and 8c polycarb(Et-*n*Oct) are shown in Table 1 (Fig. 4(A) and ESI,† Fig. S66, S67). Dispersity (*Đ*) values of approximately 2 are to be expected from a step-growth polymerisation and lower values may suggest removal of some portion of the low molecular weight species within the distributions during purification.

*T*_g_ measurement of the polymers was conducted using a heat–cool–heat cycle to avoid inaccurate measurement that may be derived from the thermal history of each polymer. A smooth heating profile was observed for all polymers and values were determined from the midpoint of the transitions (Fig. 4(B)). The polymer series 9a polycarb(Hex-*n*Bu), 9b polycarb(Hex-iBu) and 9c polycarb(Hex-*n*Oct) were also characterised using identical techniques (Table 1 and ESI,† Fig. S68–S76).

As can be seen in Table 1, the polycarbonates derived from the shorter ethylene bischloroformate had markedly higher *T*_g_ values than their hexamethylene bischloroformate analogues, as would be expected from the less flexible backbone of the polymers containing these short alkyl chains. Also, a noticeable trend to reduced *T*_g_ was seen in both polymer series with changing carbamate substitution on the pendant FTC prodrug (iBu > *n*Bu ≫ *n*Oct). This may be expected given the impact of flexible and bulky pendant groups on the conformational freedom of polymer backbones; this is most commonly seen in chain-growth polymers such as poly(i-butyl methacrylate), poly(*n*-butyl methacrylate), and poly(*n*-octyl methacrylate) with literature *T*_g_ values of 48 °C, 20 °C, and −20 °C respectively.^37^ It is interesting to note that although the carbamate substitution comprised a relatively small portion of the total FTC-derived pendant group, it was still able to markedly affect *T*_g_ values.

**Fig. 5:**
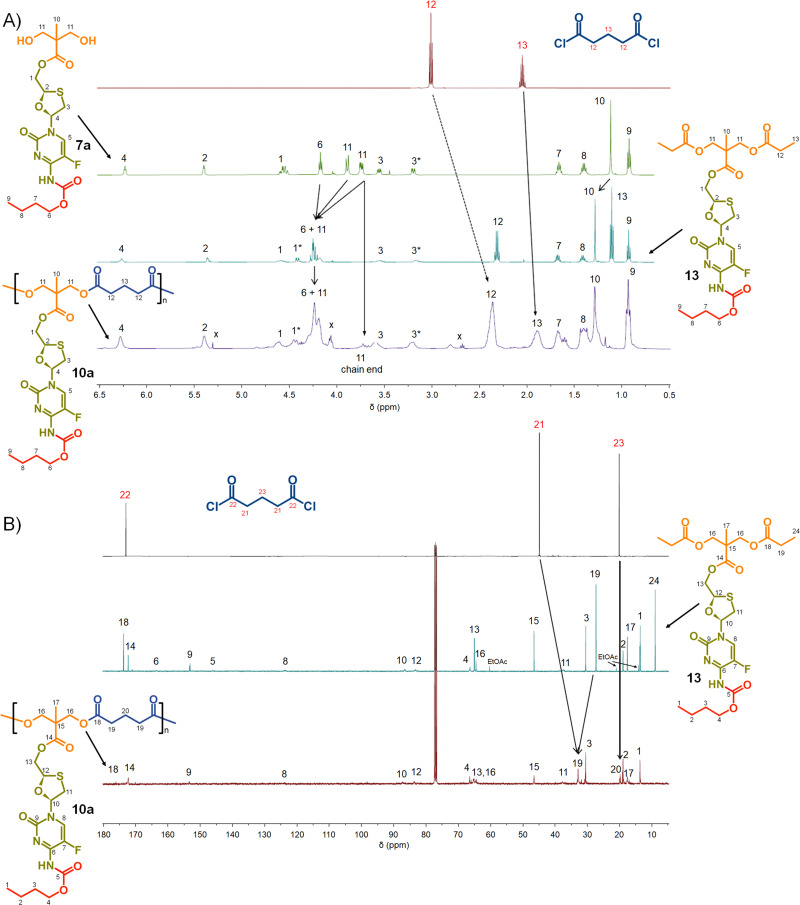
Nuclear magnetic resonance spectroscopy characterisation of polyester polymer–prodrug conjugates (typical example): (A) ^1^H NMR spectra of monomers 7a and glutaryl chloride, model compound 13 and polymer 10a, and (B) ^13^C NMR spectra of monomers glutaryl chloride, model compound 13, and polymer 10a.

Turning to the analogous polyester materials, the synthesis of polymer prodrugs was achieved through the reaction of 7a–c with either glutaryl chloride or azelaic acid dichloride, Scheme 2. As an example, the synthesis of 10a polyester(Glu-*n*Bu) was confirmed using a comparison of ^1^H and ^13^C NMR spectra against 7a and the model diester 13, Fig. 5.

As seen in the analogous comparison of NMR spectra for the series of carbonate backbone polymers, some of the resonances appear to be suppressed when analysing this polyester series, potentially due to the slow relaxation from the polymer structures or the complex nature of the FTC drug structure. This correlates to reported observations of small molecule prodrug synthesis from FTC and our own model structures;^20^ for example, the resonance for the F-bearing carbon C_7_ in 12 and 13 is not readily observed under these conditions, Fig. 3 and 5, and the carbamate carbonyl C_5_ is also very weak in some spectra.

**Fig. 6:**
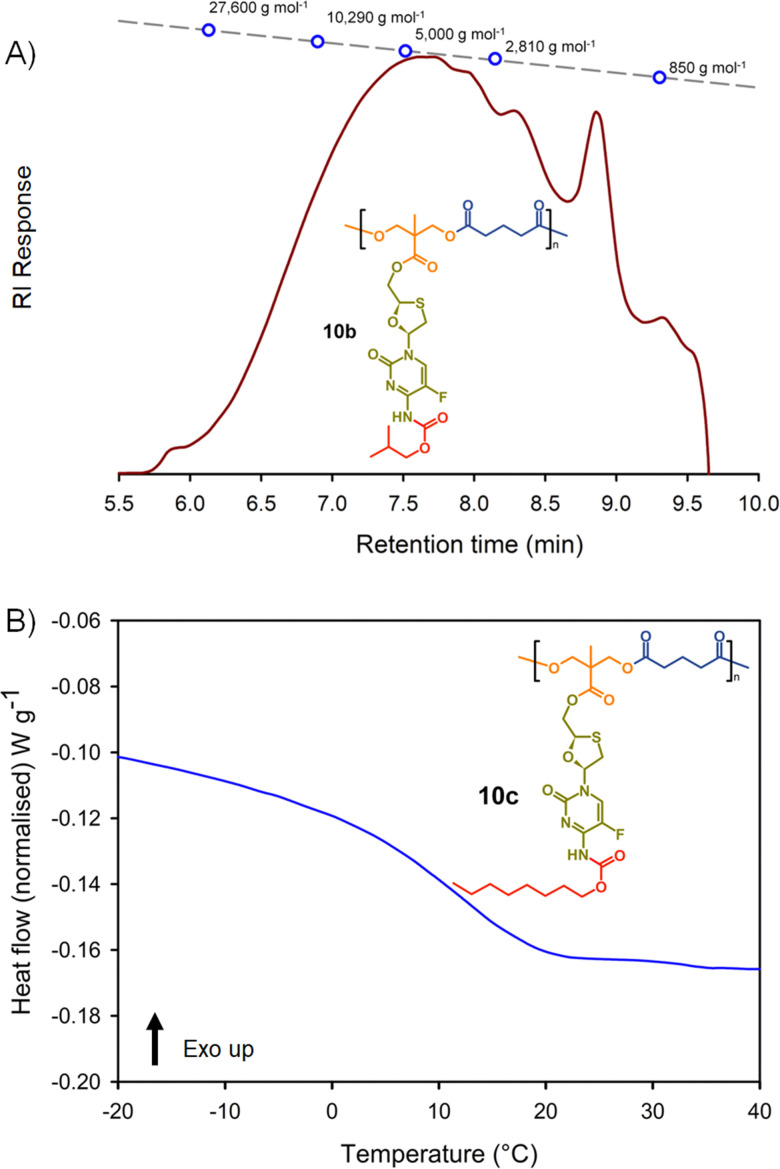
Example characterisation of polyester polymer–prodrug conjugates: (A) oligomer size exclusion chromatography (SEC) of polyester 10b, and (B) differential scanning calorimetry (DSC) characterisation of polycarbonate 10c.

The synthesis of the two polyester series were equally successful when using either glutaryl chloride or azelaic acid dichloride, leading to six systematically varying polymers, namely 10a polyester(Glu-*n*Bu), 10b polyester(Glu-iBu), 10c polyester (Glu-*n*Oct), 11a polyester(Az-*n*Bu), 11b polyester(Az-iBu), and 11c polyester(Az-*n*Oct). SEC and DSC analyses were conducted as described earlier for the polycarbonate series, Fig. 6 and ESI,† Fig. S77–S100.

The same trends that were observed across the polycarbonates were also seen throughout the six polyester structures. Dispersities and *p*(MMA) equivalent *M*_n_ and *M*_w_ values were highly comparable, and the *T*_g_ values exhibited an increase when moving from an *n*-butyl modified side chain to the iso-butyl modification, with a considerable decrease when substituting an *n*-octyl pendant group, Table 2.

**Table 2:** Size exclusion chromatography (SEC) and differential scanning calorimetry (DSC) characterisation of polyester polymer–prodrug conjugates with varying carbamate pendant group and backbone diacid monomer residues

Polymer	GPC			DSC
	*M*_n_ (g mol^−1^)	*M*_w_ (g mol^−1^)	*Đ*	*T*_g_ (°C)
10a polyester(Glu-*n*Bu)	3390	7610	2.25	30
10b polyester(Glu-iBu)	3395	5950	1.75	44
10c polyester(Glu-*n*Oct)	3255	7210	2.21	12

11a polyester(Az-*n*Bu)	4230	6020	1.42	12
11b polyester(Az-iBu)	4455	6715	1.51	20
11c polyester(Az-*n*Oct)	3940	5750	1.46	−5

### Activation of FTC-derived polymer–prodrug conjugates

The formation of polymer–prodrug conjugates is only of potential value if the polymers activate and release parent FTC under physiological conditions. The step-growth nature of the polymer backbones were selected to also cleave and breakdown into biocompatible small molecule fragments, therefore multiple products would be expected to be formed during any dual degradation–activation process.

**Fig. 7:**
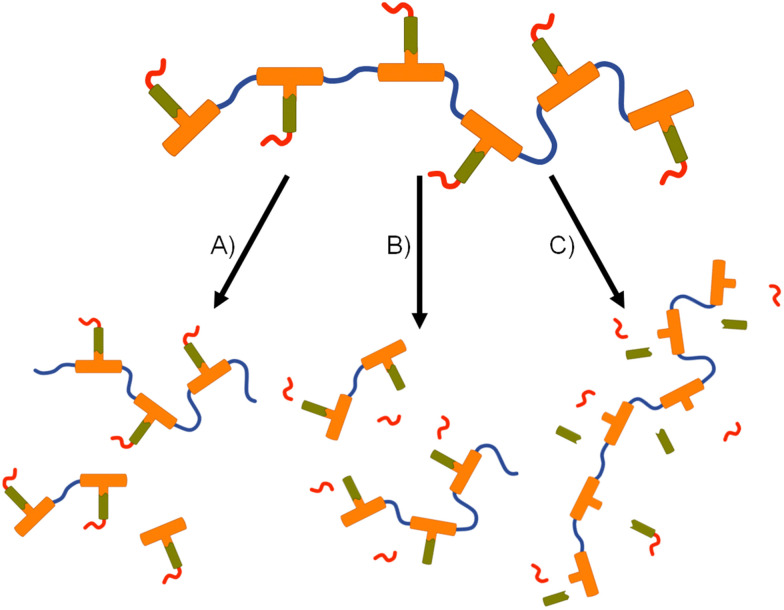
Schematic representation of potential degradation pathways for polymer–prodrug conjugates. (A) Backbone cleavage without parent drug release, (B) backbone and pendant group cleavage without parent drug release, and (C) cleavage of pendant group and drug compound from backbone without backbone degradation.

For example, backbone degradation may dominate the biological processes, Fig. 7(A), leading to delayed or no release of FTC. Alternatively, activation at the carbamate pendant-group substitution site, possibly in combination with backbone degradation, Fig. 7(B), would also not immediately release FTC. Only processes that cleave the ester linker to the backbone and the carbamate pendant group will liberate the parent drug substance, Fig. 7(C), and this may or may not be accompanied by backbone degradation on comparable timescales.

**Fig. 8:**
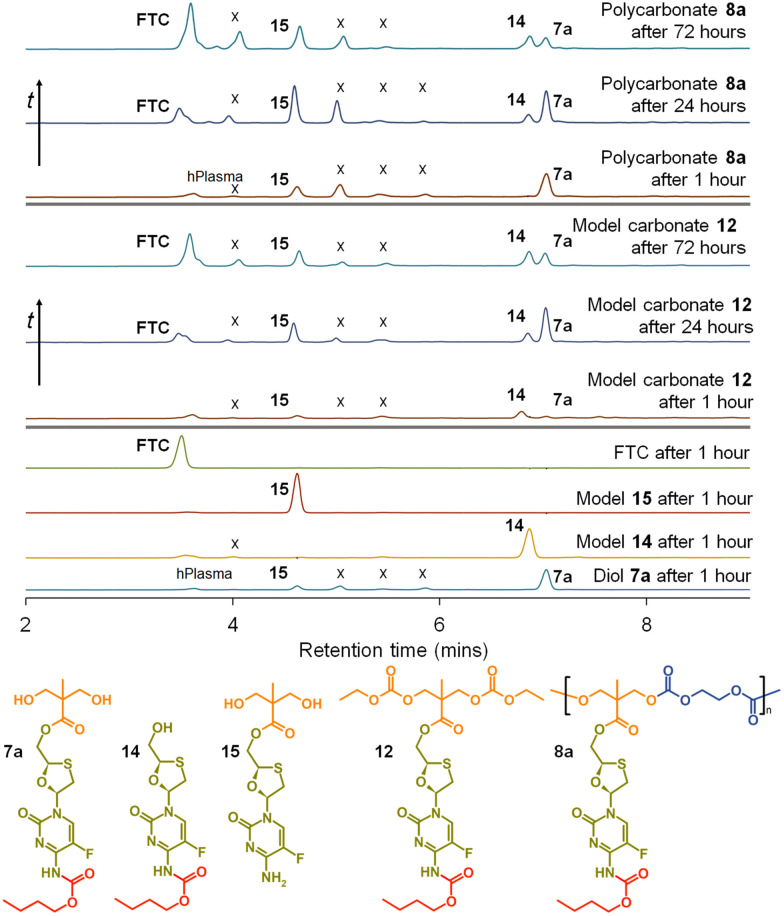
HPLC studies of emtricitabine (FTC) release from model compounds and polycarbonate 8a when exposed to mixed gender human plasma. From bottom: Exposure of diol monomer 7a, models 14 and 15, and FTC for 1 hour; exposure of model symmetric carbonate 12 for 1 hour, 24 hours and 72 hours with appearance of FTC parent drug compound; and exposure of polycarbonate polymer–prodrug conjugate 8a for 1 hour, 24 hours and 72 hours also liberating parent FTC. (X indicates unidentified species).

This is clearly complex, but with parent FTC release being the only physiologically relevant outcome of the dual degradation–activation process, a series of high-pressure liquid chromatography (HPLC) studies were conducted to detect FTC formation and determine the viability of the polymer–prodrug conjugates within their intended role as degradable, long-acting drug release implants. As all candidate polymers possess the ester and carbamate substitutions of FTC, and degradation may involve cleavage of either of these groups, the model structures 14 and 15 were included in the studies, whilst 8a polycarb(Et-*n*Bu) was selected as a representative polycarbonate. Backbone dominated degradation would yield the monomer diol 7a (and possibly 15), whilst the model carbonate 12 would provide a guide for the relative susceptibility of backbone carbonates *vs.* the ester and carbamate groups to cleavage. Comparison of the degradation of each model against the degradation of 8a polycarb(Et-*n*Bu) was expected to provide an insight into the route of cleavage and the formation of FTC parent drug. Separately each of the structures 7a, 8a polycarb(Et-*n*Bu), 12, 14 and 15 were dissolved in DMSO and added to mixed gender human plasma with subsequent incubation at 37 °C for varying times, followed by HPLC analysis using a UV wavelength of 280 nm, Fig. 8.

FTC was shown to be unaffected by incubation in mixed gender human plasma for 1 hour, as would be expected. Interestingly, the model monocarbamate 14 and monoester 15 also appeared to be relatively stable under these conditions, although a low level of degradation was observed for 14. A clear indication of the site-specific cleavage of the carbamate substitution within the monomer 7a was also seen, suggesting that the ester linkage to the polymer backbone may be more stable and supporting the findings for 14 and 15.

Incubation of the model carbonate 12, containing both the ester and carbamate substitutions, showed the clear formation of 7a, 14 and 15, after 1 hour and the release of the parent FTC. The formation of 7a indicates cleavage of the two carbonate groups, whilst 15 requires both the cleavage of the carbonates and the carbamate substituent. 14 may be present as simple cleavage of the ester group, although it may also be formed after the combined carbonate and ester cleavage; its relative concentration *vs.*7a and 15 should not therefore be over interpreted. After 24 hours of incubation, the mixed gender human plasma contained an increased concentration of FTC, 7a, and 15, whilst the 72 hours incubation sample showed a further increase in FTC and similar peak heights for 7a, 14 and 15.

These studies of model structures clearly indicate the potential for polymer–prodrug conjugates of FTC based on a polycarbonate backbone to activate all linking groups and fully release FTC. To further evaluate this potential, 8a polycarb(Et-*n*Bu) was incubated under identical conditions for a total of 72 hours. After 1 hour, the parent FTC was visible, the diol monomer repeat unit 7a was clearly observed, suggesting rapid cleavage of the backbone carbonates, and the presence of a significant concentration of 15 indicated rapid cleavage of the pendant carbamate substitution. After 24 hours the sample contained an increased concentration of FTC, the relative ratio of 7a and 15 suggested a build up of 15 in the sample, and the presence of a small peak corresponding to 14 was also seen. By the 72 hours sample, 7a had decreased considerably relative to 14 and 15, and presence of FTC dominated the analysis.

**Fig. 9:**
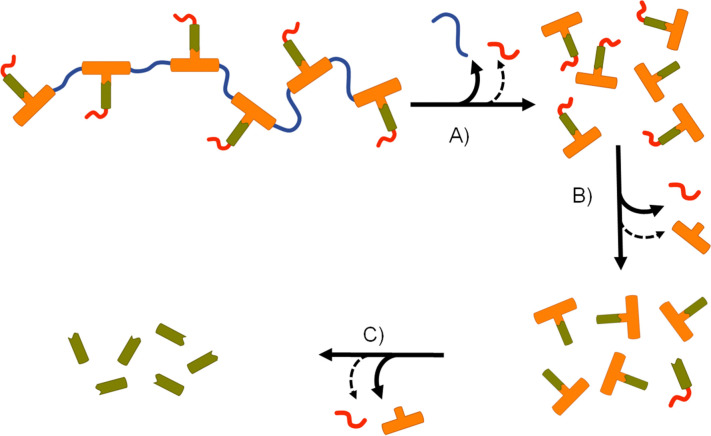
Apparent degradation and activation pathway for emtricitabine (FTC) containing polymer–prodrug conjugates: (A) rapid backbone degradation accompanied by minor cleavage of carbamate pendant groups, (B) carbamate pendant group cleavage from diol monomer units accompanied by minor ester cleavage, and (C) cleavage of ester–FTC bond releasing parent FTC.

Collectively, the data suggests an FTC release mechanism that is dominated by a rapid carbonate backbone cleavage to form the diol monomer 7a, potentially with a slow but simultaneous, removal of carbamate substitution, Fig. 9(A); no ester cleavage is observed at this stage.

15 therefore builds within the sample, directly from carbamate cleavage of 7a, although some slow ester cleavage to form 14 is evident during this phase, Fig. 9(B). Ester cleavage of 15 appears to be the dominant process in the formation of FTC with carbamate cleavage of 14 also appearing to contribute, Fig. 9(C).

A similar HPLC study of 10a polyester(Glu-*n*Bu), using 7a, 13, 14, and 15 as comparators, showed backbone ester cleavage dominating the breakdown of the polymer–prodrug conjugate, with 15 present after 1 hour and increasing through to 72 hours of incubation. 7a is present at a very low concentration after 1 hour and only shows a minor increase after 24 and 72 hours. 14 is only observed after 24 hours and does not increase significantly after 72 hours, despite the formation of an appreciable amount of FTC (ESI,† Fig. S101).

### Formation of, and drug release from, FTC-derived polymer–prodrug conjugate implants

The formation of biocompatible implantable structures from the FTC-derived polymer–prodrug conjugates requires materials that are solid at ambient temperature, thereby ruling out the low *T*_g_ materials generated during this study (8c, 9a, 9b, 9c, 10c, 11a, 11b and 11c).

**Fig. 10:**
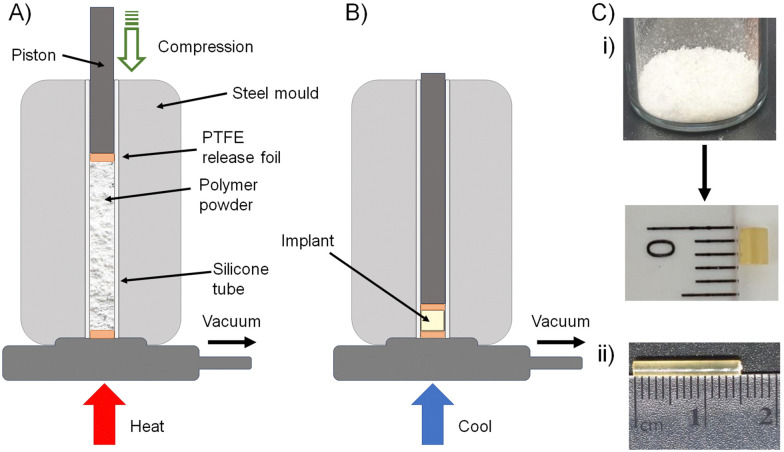
Solid implant formation from polymer–prodrug conjugates containing emtricitabine (FTC): schematic representation of (A) vacuum compression moulding apparatus after packing of powdered polymer into the mould, and (B) compression of the powdered polymer after simultaneous vacuum degassing and heating (colling allows the sample to solidify). (C) Photographs of (i) powdered polycarbonate polymer–prodrug conjugate sample before (top) and after moulding, (ii) larger homogeneous implant.

The four polymers 8a polycarb(Et-*n*Bu), 8b polycarb(Et-iBu), 10a polyester(Glu-*n*Bu), and 10b polyester(Glu-iBu) were, therefore, subjected to vacuum compression moulding (VCM) as previously described.^26^ In summary, the powdered polymers were loaded into a silicone tube that was mounted within a VCM mould, and sandwiched between PTFE release foils, Fig. 10(A). Application of vacuum removes air from the powder column during heating to 50 °C (55 °C was required in the formation of implant rods from 8b polycarb(Et-iBu)). Compression under the applied vacuum is provided by a piston above the powder and the samples were left to compress during heating for 2 minutes, followed by cooling, Fig. 10(B), and removal of the homogeneous implant rod with approximate dimensions of 2 × 2 mm, Fig. 10(C)-(i); implants of up to 15 × 2 mm were readily achievable, Fig. 10(C)-(ii).

Rod formation from the four polymers was highly successful and the implants formed from 8a polycarb(Et-*n*Bu), 8b polycarb(Et-iBu), 10a polyester(Glu-*n*Bu), and 10b polyester(Glu-iBu) showed no chain cleavage during the process, as studied by SEC, or chemical degradation, confirmed by NMR analysis (ESI,† Fig S102–S104).

**Fig. 11:**
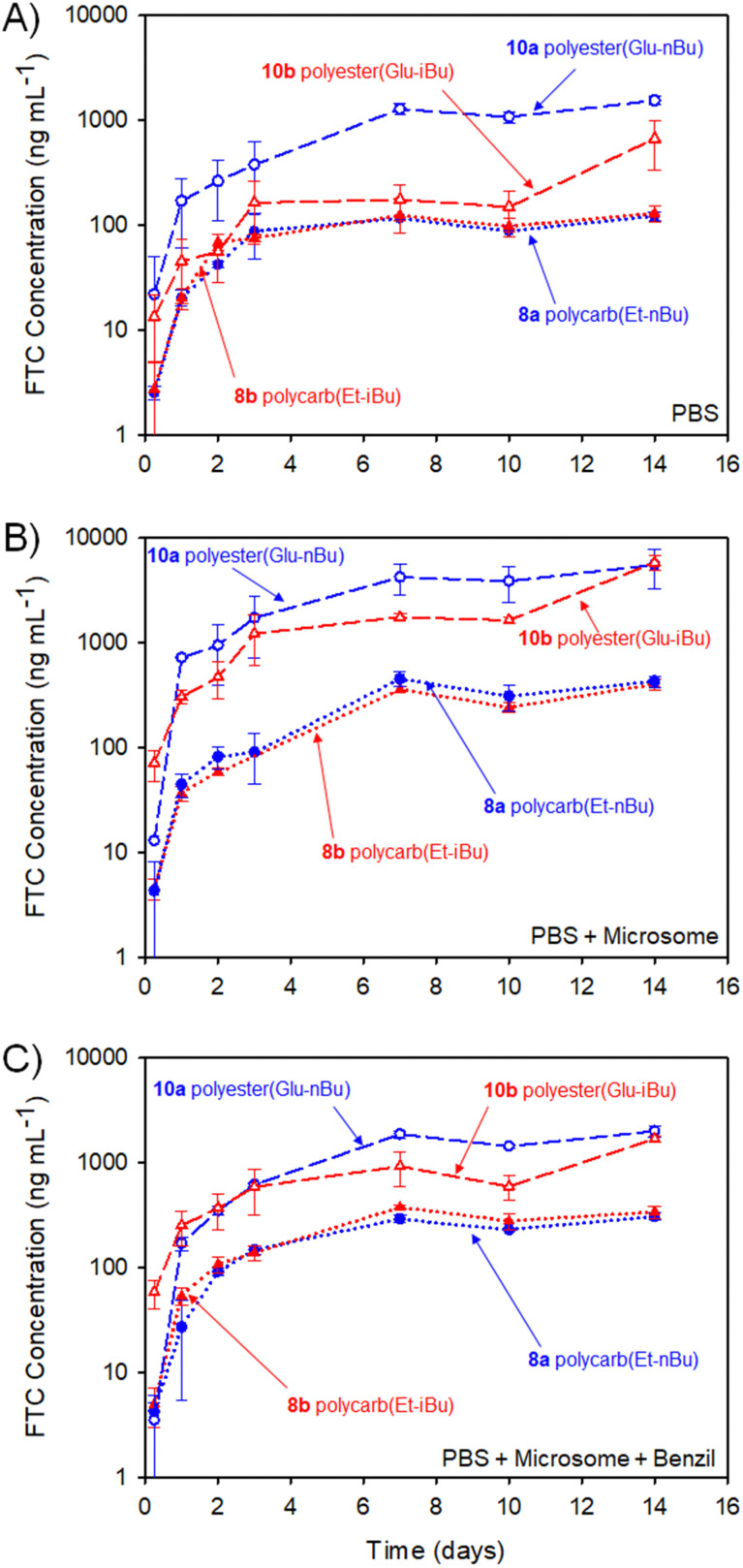
Cumulative FTC parent drug release curves (*in vitro*) over 14 days from HPLC-MS/MS studies of polyester (10a and 10b) and polycarbonate (8a and 8b) polymer–prodrug conjugate implants under different conditions (assay measures emtricitabine (FTC) concentration only). (A) FTC release from implants incubated in PBS, (B) FTC release in the presence of PBS and pooled human liver microsomes, and (C) FTC release in the presence of PBS and microsomes with added Benzil carboxylesterase inhibitor.

The implant rods were incubated (*n* = 3) in a series of media at 37 °C for 14 days with stirring (250 rpm), Fig. 11. Each implant contained 5 mg of FTC within the polymer–prodrug conjugate structure and were incubated in either phosphate buffered saline (PBS), PBS containing pooled human liver microsomes, or PBS containing microsomes and an inhibitor of carboxylesterase 1 (CES1), namely 1,2-diphenylethane-1,2-dione (also known as benzil).

The study selected human liver microsomes as these contain a range of carboxylesterases and cytochrome P450 metabolic enzymes, many of which will be present in a human implant site.^38,39^ The use of benzil to inhibit CES1 allows a preliminary indication of the enzymatic mechanisms involved in FTC release. A previously validated HPLC-mass spectroscopy assay^40^ was employed to monitor release of FTC alone, therefore no other fragments of the polymer degradation were studied. Cumulative release curves, Fig. 11, were constructed for the four polymer–prodrug conjugates under the three conditions.

As can be seen from Fig. 11(A), all implants released FTC when incubated in PBS alone. Despite hydrolysis being the most obvious mechanism in this case, the polyester-derived implants appear to be more susceptible than the polycarbonates to hydrolytic degradation, correlating well with reported comparative studies of aliphatic polymers.^41^

Importantly, the carbamate substitution must also cleave to allow detection of FTC parent drug and the chemistry of the carbamate must also be considered. For the polyesters incubated in PBS, the *n*-butyl carbamate appeared to yield the fastest formation of free FTC, with i-butyl substituted polymer–prodrug conjugates providing a slower formation of parent FTC. Whether this is due to the different carbamate contributing to the overall hydrophobicity of the polymer implant, and hence modifying ester cleavage rates, or simply due to structures analogous to 14 having a slower rate of hydrolysis, is unclear. No definitive differentiation within the polycarbonate implants was observed, but overall, the non-enzymatic release of FTC is an important factor in the activation of all prodrug-containing implants studied here.

In the presence of the metabolic enzymes within the human liver microsome environment, the rate of FTC release was considerably enhanced across all implants studied. The difference in the two polyester derived polymer–prodrug conjugates was still apparent with the *n*-butyl carbamate substitution providing a faster release than the i-Bu substituted material and this ordering was also observable, but not statistically relevant, across the polycarbonate analogues. The faster release from the polyesters *vs.* polycarbonates was maintained, and this is also in agreement with previous studies of aliphatic step-growth polymer degradation under enzymatic conditions.^41^

A noticeable reduction in FTC release was observed across all implants when benzil was added to the PBS/microsome medium. This is in accordance with the inhibition of CES1 but there was still a clear enhanced release for all implants when compared to PBS alone. This suggests that other enzymatic mechanisms, not inhibited by benzil, are also contributing to the activation of the polymer–prodrug conjugates in the presence of microsomes.

## Conclusions

Long-acting therapeutics offer extended systemic drug exposure over prolonged periods. Many clinical products target timescales over multiple months from a single administration, however, drugs with short half-lives are particularly challenging to formulate successfully. Drug compounds with appreciable water-solubility offer particular problems, but many conventional medicines (*e.g.*, orally dosed products) utilise drugs with a wide range of physical properties and often rely upon molecules such as FTC as the foundation for efficacy.

Altering the solubility of existing drug compounds through prodrug strategies is well established, but here we have conjugated the prodrug to a polymer backbone in order to generate solid objects as implant candidates. Under the physiological conditions studied, FTC formation is clearly seen to be maintained for at least 2 weeks.

Although the polyester candidates provided a faster release, the slower evolution of FTC from polycarbonates bodes well for the provision of options that may deliver FTC for periods of >1 month. Prolonged drug release from these implants is expected to be seen in future *in vivo* studies; however, the plasma concentrations will need to lie within the therapeutic window to be efficacious, and long release below the minimum effective concentration would be of little value. It is reassuring that two specific opportunities exist to tune release within an *in vivo* study, namely backbone chemistry and pendant carbamate substitution. There is also considerable potential for the synthesis of backbone copolymers, to blend carbonate and ester links, and copolymers derived from mixed monomers containing variation in carbamate chemistry. When compared to our previous reports of polymers containing FTC within the backbone,^26^ we have a considerably slower release of parent drug during activation which may be highly important when evaluating these approaches *in vivo*.

It is also important to note that the opportunity described here presents an opportunity to moderate the release of FTC, but drug combinations are required for a full HIV regimen. Establishing the creation of relevant circulating FTC concentrations and matching the pharmacokinetics with relevant additional drugs is the next step and *in vivo* studies will be required. The use of polymer–prodrug conjugates in long-acting degradable implants may also have application that extends significantly from the antiretroviral example shown here.

## Author contributions

SPR conceptualised the polymer chemistry, supervised the experimental polymer and materials chemistry, wrote the draft and edited the final manuscript. SPR, AO, CFM wrote the grant proposal and secured the funding. CL, FYH and AS carried out the synthetic work and characterisation. PC and TOM co-supervised the synthetic programme with SPR, CFM supervised the analysis of the materials by HPLC methodology in the presence of mixed human plasma; KT conducted these experiments. AO and NL supervised the analysis of the materials by HPLC-MS/MS methodologies in the presence of microsomes; MN conducted these experiments and analysed the data.

## Conflicts of interest

SPR, AO, CL, FYH, AS, KT, MN, AO, CFM and SPR are coinventors on a published patent application (WO2020128525) describing the presented polymers.

## Supplementary Material

TB-011-D3TB02268D-s001
