# Supplementary material for: Polymer–prodrug conjugates as candidates for degradable, long-acting implants, releasing the water-soluble nucleoside reverse-transcriptase inhibitor emtricitabine
Source: J Mater Chem B. 2023 Nov 7;11(48):11532–43. doi: 10.1039/d3tb02268d (PMC10718295; doi:10.1039/d3tb02268d)
Supplement: TB-011-D3TB02268D-s001 [file TB-011-D3TB02268D-s001.pdf]

Electronic Supplementary Material (ESI) for Journal of Materials Chemistry B.
This journal is © The Royal Society of Chemistry 2023

Polymer-prodrug conjugates as candidates for degradable, long-acting implants, releasing the water-soluble nucleoside reverse-transcriptase inhibitor emtricitabine.

Chung Liu, Faye Y. Hern, Anika Shakil, Kartik Temburnikar, Pierre Chambon, Neill Liptrott, Tom O. McDonald, Megan Neary, Charles Flexner, Andrew Owen, Caren Freel Meyers and Steve P. Rannard[*]

Corresponding author E-mail: srannard@liv.ac.uk

Supplementary information

Experimental

Materials

FTC was purchased from Wischem. Isobutyl chloroformate, (98 %), *n*-pentyl chloroformate (97 %), *n*-hexyl chloroformate (98 %) and *n*-octyl chloroformate (97 %) were purchased from Tokyo Chemical Industries. Ethyl chloroformate (97 %), *n*-propyl chloroformate (98 %), *n*-butyl chloroformate (98 %), neopentyl chloroformate (97 %), propionyl chloride (98 %), pyridine (anhydrous, 99.8 %), dichloromethane (DCM, analytical grade), *n*-hexane (HPLC grade), ethyl acetate (EtOAc, analytical grade), tetrahydrofuran (THF, HPLC grade), acetone (analytical grade), methanol (MeOH, HPLC grade), *N, N*-dimethylformamide (DMF, analytical grade, 99.5 %), lithium bromide (LiBr, anhydrous, 99 %), lithium hydroxide (LiOH, anhydrous, 98%), triethylamine (TEA, 99+ %), 4-(dimethylamino)pyridine (DMAP, 99 %), magnesium sulphate (MgSO4, anhydrous, 99.5+ %), hydrochloric acid (HCl, 12.2 M), silica gel used for column chromatography, and silica-coated aluminium plates used for analytical TLC were purchased from Fisher Scientific. Chloroform-d ($CDCl_3$, 99.8 % atom D), dimethyl sulphoxide-d6 (DMSO-d6, 99.9 % atom D), 2,2-bis(hydroxymethyl)propionic acid (bis-MPA, 98 %), toluene (anhydrous, analytical grade), 2,2-dimethoxypropane (98 %), para-toluenesulfonic acid monohydrate (98.5 %), DOWEX® (hydrogen form, 100-200 mesh), ethylene bischloroformate (98 %), glutaryl chloride (97 %), diglycolyl chloride (95 %), azelaic acid dichloride (98 %) were purchased from Merck. Prepacked silica gel columns for automated flash chromatography were purchased from Büchi. All materials were used as received.

Characterisation

NMR spectra were recorded in $CDCl_3$ or DMSO-d_6 using a Bruker Avance III HD spectrometer operating at 500 MHz. 1H NMR were recorded at 500 MHz, and 13C NMR were recorded at 100 MHz. Chemical shift (δ) values are reported in parts-per-million (ppm), and trimethylsilane (TMS) was used as the internal standard for both 1H and 13C NMR spectra. Electrospray mass spectrometry (ESI-MS) data were recorded in the Mass Spectrometry Laboratory at the University of Liverpool, Department of Chemistry, using an Agilent 6200 series TOF and 6500 Series Q-TOF LC/MS system. All materials were solubilised in methanol. Infrared spectrometry (IR) data were recorded using a Vertex 70 FT-IR spectrometer equipped with an attenuated total reflection (ATR) germanium crystal. All spectra were recorded in the transmission mode. Size exclusion chromatography (SEC) data were recorded using an Agilent 1260 Inifinity II system equipped with a PL-Gel Mixed E column and a guard column, and a single RI detector, with a mobile phase of DMF with 0.01 M LiBr at 60 °C and at a flow rate of 1 mL min^{-1}. Differential scanning calorimetry (DSC) data were recorded using a TA Instrument DSC 2500 system calibrated using an indium calibration standard, and the samples were obtained using a TZero® aluminium hermetic pans under a N_2 atmosphere. High-performance liquid chromatography (HPLC) data were recorded using an Agilent Infinity 1260 System equipped with an InfinityLab Poroshell 120 column, using an eluent system consisting of 50 mM ammonium acetate (NH4OAc), pH 6 and acetonitrile (CH_3CN). Liquid chromatography-mass spectrometry (LC-MS) was performed on a SCIEX 6500+ 273 Experimental QTRAP LC-MS/MS system equipped with Kinetex® F5 column. Mobile phases A and B consisted of H_2O with 0.1% acetic acid and MeOH with 0.1% acetic acid, respectively.

Synthesis of monoacetylated FTC product 1. FTC (10 mmol, 1 eq.) and DMAP (10 mmol, 1.22 g, 1 eq.) were added to a 250 mL round bottom flask fitted with a magnetic stirrer and an N_2 inlet. DCM (100 mL) was added to the flask, followed by the addition of pyridine (300 mmol, 23.7 mL, 30 eq.) and then the reaction mixture was cooled in an ice-water bath for approximately 10 min. Acetic anhydride (10 mmol, 0.95 mL, 1 eq.) was then added dropwise to the stirring FTC solution. Upon complete addition, a pale-yellow solution evolved. The reaction was warmed slowly to ambient temperature and stirred for 16 hrs, after which the reaction was deemed complete by TLC with the disappearance of FTC. The crude products were diluted with DCM (100 mL) and washed with HCl (1 M, 2 x 100 mL) and brine solution (100 mL). The organic layer was dried over MgSO4, filtered, and DCM was removed in vacuo. The recovered products were purified using automated flash chromatography on a silica column (normal phase, solvent gradient: 40 - 100 % EtOAc in n-hexane over 45 min). 1.27 g of white solids (43.9 %). 1H NMR: δ .91 (d, 1H), 6.31 (ddd, 1H), 5.36 (dd, 1H), 4.60 (dd, 1H), 4.45 (dd, 1H), 3.57 (dd, 1H), 3.18 (dd, 1H), 2.16 (s, 3H). 13C NMR: δ 170.3, 158.3, 158.2, 153.6, 137.6, 135.2, 125.5, 125.2, 87.3, 84.3, 63.5, 38.8, 20.7. ESI-MS: calc. $[M+H]^+$ = 290.05 Da, found 290.05 Da.

Synthesis of acetonide-protected BMPA 2. Bis-MPA (0.75 mol, 100 g, 1 eq.), 2,2-dimethoxy propane (1.12 mol, 140.0 mL, 1.5 eq.) and para-toluenesulfonic acid monohydrate (37.2 mmol, 7.09 g, 0.02 eq.) were added to a 1 L round bottom flask fitted with a magnetic stirrer bar. Acetone (500 mL) was added, and the resulting solution was stirred at ambient conditions for 3 hours. The reaction was stopped by adding TEA (74.4 mmol, 10.3 mL, 0.04 eq.) and acetone was removed in vacuo. The crude products were dissolved in DCM (500 mL) and washed three times with distilled water (250 mL) and brine solution (100 mL), after which the organic layer was dried over MgSO4 and filtered. White solids (75.4 g, 58.1%) were recovered following the removal of DCM in vacuo. 1H NMR: δ 11.34 (s, 1H), 4.20 (d, J = 11.8 Hz, 2H), 3.67 (d, J = 11.8 Hz, 2H), 1.43 (d, J = 16.2 Hz, 6H), 1.22 (s, 3H). 13C NMR: δ 180.33, 98.32, 65.79, 41.74, 25.00, 22.12, 18.46. ESI-MS: calc. $[M-H]^-$ =173.09 Da, found 173.09 Da.

Synthesis of symmetrical BMPA anhydride 3. Acetonide-protected bis-MPA 3 (0.12 mol, 20 g, 2 eq.) and DCM (100 mL) were added to a 250 mL round bottom flask equipped with a magnetic stirrer bar and stirred at ambient conditions. DCC (0.06 mol, 11.8 g, 1 eq.) was added to the stirring solution. The evolution of DCU as white solids was observed as the reaction progressed. The reaction was stopped after stirring at ambient conditions for 48 hrs. DCU was filtered off using a Büchner funnel; a total of three filtrations were performed. The crude products were concentrated in vacuo, and then purified by precipitation into cold nhexane (chilled in an acetone and dry-ice bath). DCM was removed in vacuo, and the recovered products were dissolved in EtOAc and chilled to 5 °C for 24 hrs to further remove residual DCU by filtration. Following the removal of EtOAc, a pale yellow liquid (31.1 g, 78.5 %) was recovered. 1H NMR: δ 4.05 (m, 2H), 3.55 (m, 2H), 1.28 (s, 3H), 1.23 (s, 3H), 1.07 (s, 3H). 13C NMR: δ 169.39, 98.18, 65.51, 43.55, 25.66, 21.22, 17.42, 14.04.

Synthesis of BMPA-FTC monoester 5. FTC (10 mmol, 1 eq.) and DMAP (10 mmol, 1.22 g, 1 eq.) were added to a 250 mL round bottom flask fitted with a magnetic stirrer and an N_2 inlet.

DCM (100 mL) was added to the flask, followed by the addition of pyridine (300 mmol, 23.7 mL, 30 eq.) and then the reaction mixture was cooled in an ice-water bath for approximately 10 min. BMPA anhydride **4** (10 mmol, 3.30 g dissolved in 10 mL DCM, 1 eq.) was then added dropwise to the stirring FTC solution. Upon complete addition, a pale-yellow solution evolved. The reaction was warmed slowly to ambient temperature and stirred for 16 hrs, after which the reaction was deemed complete by TLC with the disappearance of FTC. The crude products were diluted with DCM (100 mL) and washed with HCl (1 M, 2 x 100 mL) and brine solution (100 mL). The organic layer was dried over MgSO4, filtered, and DCM was removed in vacuo. The recovered products were purified using automated flash chromatography on a silica column (normal phase, solvent gradient: 40 - 100 % EtOAc in n-hexane over 45 min). 2.64 g of off-white solids (63.0 %). 1H NMR: δ 7.77 (m, 1H), 6.27 (t, 1H), 5.37 (dt, 1H), 4.66 (dt, 1H), 4.44 (dt, 1H), 4.20 (m, 2H), 3.67 (dt, 2H), 3.55 (ddd, 1H), 3.13 (ddd, 1H), 1.42 (s, 3H), 1.35 (s, 3H), 1.17 (s, 3H). 13C NMR: δ 173.9, 158.1, 153.5, 137.6, 135.2, 124.8, 98.3, 87.5, 83.3, 66.0, 64.3, 42.2, 38.1, 25.4, 21.8, 18.5. ESI-MS: calc. $[M+H]^+$ = 420.12 Da, found 420.12 Da.

General procedure for the synthesis of alkyl-carbamate, BMPA-ester products 6a-c. In a typical synthesis, BMPA-FTC monoester **5** (23.86 mmol, 10 g, 1 eq.) was added to a 250 mL round bottom flask fitted with a magnetic stirrer bar and fitted with a N_2 inlet. Pyridine (28.63 mmol, 2.26 mL, 1.2 eq.) and DCM (100 mL) were then added to the flask, and the resulting pale-yellow solution was chilled in an ice-water bath for approximately 5 min. Alkyl chloroformates (28.63 mmol, 1.2 eq.) was then added dropwise to the chilled solution. The reaction mixture warmed slowly to ambient temperature and stirred for 16 hrs, after which the reaction was deemed complete by TLC with the disappearance of monoester **5**. The reaction mixture was diluted with DCM (100 mL), and washed with HCl (1 M, 2 x 100 mL) and brine solution (100 mL). The organic layer was dried over MgSO4, filtered, and DCM was removed in vacuo. The recovered materials were purified further using automated flash chromatography on a silica column (normal phase, solvent gradient: 20 to 70 % EtOAc in n-hexane over 30 min).

***n*-Butyl carbamate BMPA-ester product 6a:** 10.3 g (87%) of pale yellow semi-solid. 1H NMR: δ 7.88 (bs, 1H), 6.28 (bs, 1H), 5.41 (bt, 1H), 4.69 (bdd, 1H), 4.48 (dd, 1H), 4.21 (bm, 4H), 3.66 (dd, 2H), 3.56 (bs, 1H), 3.20 (bdd, 1H), 1.69 (m, 2H), 1.43 (m, 5H), 1.37 (s, 3H), 1.17 (s, 3H), 0.94 (t, 3H). 13C NMR: δ 173.9, 163.6, 153.2, 146.0, 138.4, 124.1, 98.2, 86.6, 83.8, 66.4, 66.0, 63.9, 42.3, 37.7, 30.5, 25.9, 21.2, 19.0, 18.4, 13.7. ESI-MS: calc. $[M+H]^+$ = 504.17 Da, found 504.17 Da.

***i-Butyl* carbamate BMPA-ester product 6b:** 10.2 g (85%) of yellow semi-solid. 1H NMR: δ 7.86 (s, 1H), 6.21 (ddd, 1H), 5.35 (dd, 1H), 4.64 (dd, 1H), 4.41 (dd, 1H), 4.14 (ddd, 2H), 3.90 (d, 2H), 3.61 (dd, 2H), 3.52 (dd, 1H), 3.15 (dd, 1H), 1.94 (dt, 1H), 1.36 (s, 3H), 1.29 (s, 3H), 1.11 (s, 3H), 0.90 (d, 6H). 13C NMR: δ 173.8, 153.2, 146.9, 125.3, 98.2, 87.0, 83.9, 72.6, 65.9, 63.9, 42.2, 37.8, 27.6, 25.7, 21.3, 19.0, 18.3. ESI-MS: calc. [M+H]+ = 504.17 Da, found 504.17 Da.

***n*-Octyl carbamate BMPA-ester product 6c:** 10.7 g (80%) of pale-yellow semi-solid. 1H NMR: δ 7.87 (bs, 1H), 6.27 (bs, 1H), 5.41 (bs, 1H), 4.69 (bd, 1H), 4.48 (d, 1H), 4.20 (bm, 4H), 3.69 (d, 2H), 3.56 (bs, 1H), 3.21 (bd, 1H), 1.70 (m, 2H), 1.43 (s, 3H), 1.37 (bm, 5H), 1.27 (bm, 9H), 1.17 (s, 3H), 0.88 (s, 3H). 13C NMR: δ 173.9, 153.2, 146.0, 140.7, 124.1, 98.3, 86.6, 83.7, 66.7, 66.0, 66.0, 63.9, 42.3, 37.7, 31.8, 29.1, 28.6, 25.8, 22.6, 21.2, 18.4, 14.1. ESI-MS: calc. [M+H]+ = 560.24 Da, found 560.24 Da.

General procedure for the synthesis of diol monomers 7a-c. In a typical synthesis, alkyl-carbamate BMPA-ester products **6a-6c** (5 mmol) was added to a 250 mL round bottom flask fitted with a magnetic stirrer bar. MeOH was added to the flask to yield a final concentration of 0.02 g mL^{-1} of **6a-6c**. Following complete dissolution, the reaction mixture was heated to 50 °C. DOWEX® 50WX2 resin (50 wt% relative to **6a-6c**) was added and the resulting turbid orange solution was stirred at 50 °C. The reaction was monitored by TLC and was deemed complete after 1 hr with the disappearance of**6a-6c**. The DOWEX® resin were filtered using a Büchner funnel. MeOH was removed from the filtrate in vacuo and the resulting diol monomers **7a-7c** were dried under vacuum at 50 °C for at least 48 hrs before use.

***n*-Butyl carbamate diol monomer 7a.** 2.00 g of orange semi-solid (86.5 %). 1H NMR: δ 7.94 (d, 1H), 6.23 (s, 1H), 5.40 (dd, 1H), 4.56 (qd, 2H), 4.17 (t, 2H), 3.89 (d, 2H), 3.74 (dd, 2H), 3.57 (d, 1H), 3.20 (dd, 1H), 1.66 (m, 2H), 1.40 (h, 2H), 1.12 (s, 3H), 0.92 (t, 3H). 13C NMR: δ 175.2, 157.5, 153.3, 148.8, 139.3, 125.9, 87.1, 83.8, 67.0, 66.5, 63.8, 49.6, 37.7, 30.5, 19.0, 17.2, 13.7. ESI-MS: calc. [M+H]+ = 464.14 Da, found 464.14 Da.

***i*-Butyl carbamate diol monomer 7b.** 2.05 g of orange semi-solid (86.9 %). 1H NMR: δ 7.95 (bs, 1H), 6.25 (bs, 1H), 5.42 (bs, 1H), 4.57 (m, 2H), 3.93 (m, 4H), 3.78 (m, 2H), 3.55 (bs, 1H), 3.22 (m, 3H), 2.01 (m, 1H), 1.13 (s, 3H), 0.97 (d, 6H). 13C NMR: δ 175.3, 163.1, 153.5, 146.1, 124.3, 86.9, 83.8, 72.7, 67.3, 63.8, 49.6, 37.7, 27.7, 19.0, 17.2. ESI-MS: calc. [M+H]+ = 464.14 Da, found 464.14 Da.

***n*-Octyl carbamate diol monomer 7c.** 2.19 g of orange semi-solid (84.5 %). 1H NMR: δ 7.93 (s, 1H), 6.25 (s, 1H), 5.41 (s, 1H), 4.56 (s, 2H), 4.18 (t, 2H), 3.92 (d, 2H), 3.76 (dd, 2H), 3.56 (q, 1H), 3.20 (dd, 3H), 1.69 (t, 2H), 1.30 (m, 11H), 1.13 (s, 3H), 0.88 (m, 3H). 13C NMR: δ 175.3, 163.2, 153.4, 146.5, 124.6, 86.9, 83.8, 67.5, 67.3, 66.8, 63.8, 49.6, 37.6, 31.7, 29.2, 29.1, 28.6, 25.8, 22.6, 17.2, 14.2, 14.1. ESI-MS: calc. [M+H]+ = 520.21 Da, found 520.21 Da.

General procedure for the polymerisation of polymers 8a-c, 9a-c, 10a-c and 11a-c. In a typical synthesis, diol monomer (7a-c, 4 mmol, 1 eq.) was added to a 50 mL round bottom flask fitted with a magnetic stirrer bar and a N_2 inlet. The reaction flask was then heated to 60 °C and following the melting of the diol monomer, pyridine (50 wt% relative to **6a-c**) was added. The resulting viscous solution was stirred for a further 10 min, after which ethylene bischloroformate (to generate polycarbs **8a-8c**, 4 mmol, 0.52 mL, 1 eq.), or hexamethylene bischloroformate (to generate polycarb **9a-9c**, 4 mmol, 0.76 mL, 1 eq.), or glutaryl chloride (to generate polyester **10a-c**, 4 mmol, 0.51 mL, 1 eq.), or azelaic acid dichloride (to generate polyester **11a-c,** 4 mmol, 0.79 mL, 1 eq.) was added to the viscous solution, resulting in a highly viscous solution which was stirred slowly (50 revolutions per minute (RPM)) for 18 hrs. The crude products were diluted in DCM (200 mL), and washed with HCl (1 M, 3 x 100 mL),

distilled water (2 x 100 mL) and brine solution (100 mL). The organic layer was dried over MgSO4, filtered, and DCM was removed in vacuo. The polymers were analysed by NMR, SEC and DSC; spectra provided.

Synthesis of model dicarbonate compound 12 and model diester compound 13. In a typical synthesis, diol monomer **6a** (4.32 mmol, 2 g, 1 eq.), pyridine (8.64 mmol, 0.68 mL, 2 eq.) and DCM (100 mL) were added to a 100 mL round bottom flask fitted with a magnetic stirrer and a N_2 inlet. After cooling in an ice-water bath for approximately 10 min, ethyl chloroformate (to generate model dicarbonate **12,** 9.50 mmol, 0.90 mL, 2.2 eq.) or propionyl chloride (to generate model diester **13,** 9.50 mmol, 0.86 mL, 2.2 eq) was then added dropwise to the stirring solution and the reaction mixture warmed slowly to ambient temperature. After stirring for 4 hrs, the reaction was deemed complete by TLC with the disappearance of FTC diol monomer 6a. The reaction mixture was diluted with DCM (100 mL), and washed with HCl (1 M, 2 x 100 mL) and brine solution (100 mL). The organic layer was dried over MgSO4, filtered, and DCM was removed in vacuo. The recovered products were purified further using automated flash chromatography on a silica column (normal phase, solvent gradient: 0 - 50 % EtOAc in n-hexane over 30 min).

Model dicarbonate compound 12: 1.80 g of colourless semi-solid (68.5 %). 1H NMR: δ 7.77 (s, 1H), 6.25 (s, 1H), 5.37 (s, 1H), 4.59 (m, 1H), 4.46 (dd, 1H), 4.33 (m, 4H), 4.17 (q, 6H), 3.55 (s, 1H), 3.16 (s, 1H), 1.67 (m, 2H), 1.42 (h, 2H), 1.29 (m, 9H), 0.93 (t, 3H). 13C NMR: δ 172.0, 163.6, 154.6, 153.2, 146.1, 138.6, 127.9, 124.0, 86.7, 83.0, 68.1, 66.4, 64.4, 46.7, 37.3, 30.6, 19.0, 17.5, 14.2, 13.7. ESI-MS: calc. [M+H]+ = 608.18 Da, found 608.19 Da.

Model diester compound 13: 1.60 g of colourless semi-solid (64.4 %). 1H NMR: δ 7.78 (s, 1H), 6.27 (s, 1H), 5.36 (s, 1H), 4.59 (s, 1H), 4.41 (s, 1H), 4.25 (s, 6H), 3.56 (s, 1H), 3.18 (s, 1H), 2.31 (q, 4H), 1.68 (dq, 2H), 1.42 (m, 2H), 1.28 (s, 3H), 1.11 (t, 6H), 0.93 (t, 3H). 13C NMR: δ 173.8, 172.4, 153.2, 146.1, 123.7, 86.7, 83.2, 66.4, 65.1, 64.5, 46.6, 37.5, 30.6, 27.4, 19.0, 17.6, 13.7, 9.0. ESI-MS: calc. [M+H]+ = 575.19 Da, found 575.20 Da.

Synthesis of *n*-butyl monocarbamate product 14. FTC (8.08 mmol, 2.0 g, 1 eq.) was added to a 100 mL round bottom flask fitted with a magnetic stirrer bar fitted with a N_2 inlet. DCM (50 mL) and pyridine (8.88 mmol, 0.70 mL, 1.1 eq.) were added to the flask, and the resulting mixture was chilled in an ice-water bath and stirred for approximately 10 mins. *n*-Butyl chloroformate (8.88 mmol, 1.1 eq.) was added dropwise to the stirring mixture over a period of 30 mins. The reaction warmed slowly to ambient temperature and stirred for 16 hrs, after which the reaction was deemed complete by TLC with the disappearance of FTC. The crude products were diluted in DCM (200 mL), then purified by washing with HCl (1M, 2 x 100 mL) and brine solution (100 mL). The organic layer was dried over MgSO4, filtered, and concentrated in vacuo. The recovered products were further purified by automated flash chromatography on a silica column (normal phase, solvent gradient: 10 % - 70 % EtOAc in n-hexane over 30 min, gradient held during elution of products). 1.68 g of pale yellow semi-solid (60.0 %). 1H NMR: δ 8.54 (bs, 1H), 6.25 (bs, 1H), 5.31 (bs, 1H), 4.17 (m, 3H), 4.01 (dd, 1H), 3.50 (m, 1H), 3.27 (dd, 1H), 1.67 (m, 2H), 1.41 (m, 2H), 0.93 (m, 3H). 13C NMR: δ 163.4, 153.6, 146.1, 126.2, 88.6, 87.2, 66.3, 62.1, 38.8, 30.5, 19.0, 13.6. ESI-MS: calc. [M+H]+ = 348.10 Da, found 348.10 Da.

Synthesis of model diol-monoester 15. BMPA-FTC monoester **5** (9.92 mmol, 4 g) was added to a 250 mL round bottom flask fitted with a magnetic stirrer bar. Methanol (100 mL) was added to the flask, and a pale orange solution evolved. DOWEX® resins (100 wt% relative to prodrug 7) was added to the solution, and the reaction mixture was heated to 50 °C for 1 hr, after which the reaction was deemed complete by TLC with the disappearance of **5**. DOWEX® resin were removed using a Büchner funnel. MeOH was removed in vacuo to reveal an orange solid (2.32 g, 64.5 %). 1H NMR: δ 8.30 (s, 2H), 8.00 (d, 1H), 6.17 (td, 1H), 5.37 (dd, 1H), 4.90 (s, 2H), 4.40 (t, 2H), 3.49 (m, 5H), 3.21 (dd, 1H), 1.08 (s, 3H). 13C NMR: δ 174.9, 156.9, 151.6, 137.2, 135.3, 126.5, 126.2, 87.1, 82.7, 64.4, 64.2, 50.8, 36.4, 17.3. ESI-MS: calc. $[M+H]^+$ = 364.09 Da, found 364.09 Da.

HPLC degradation studies of polymer-prodrug conjugate rods. Diol monomer **7a**, model molecules **12, 13**, **14, 15** and polymers **8a** and **10a** were each dissolved in DMSO to yield 20 mM FTC stock solutions, based on the w/w% of FTC in the small molecules. Each FTC-containing stock (5 µL) was incubated in human plasma (hPlasma, male and female mixed, 95 µL) at 37 °C, resulting in a final FTC concentration = 1 mM. Aliquots of this mixture (10 µL, 10 nmoles of FTC) were withdrawn at 1 h, 24 h and 72 h, and added to phosphate buffer solution (PBS) (190 µL, 100 mM, pH 7.4), resulting in a final FTC concentration = 0.05 nM. This diluted mixture was centrifuged at 14,000 RPM in 3 kDa molecular weight cut off centrifugal filters for 10 mins, to separate FTC from the proteins. 20 µL (1 nmoles of FTC) of the filtrate was analysed by high-performance liquid chromatography (HPLC; solvent A: 50 mM ammonium acetate (NH4OAc), pH 6. Solvent B: Acetonitrile (CH3CN). Gradient of 95% to 100% solvent A over 15 mins). The percentage release of FTC was calculated using an FTC standard curve (sample size of three, n = 3), and based on an expected amount of 1 nmole of FTC per injection.

***in vitro* drug release studies of polymer-prodrug conjugate rods.** Polymer rods were incubated in 1 mL of microsome (125 µg/mL, diluted in phosphate buffer saline (PBS)) containing 8.62 µg/mL total carboxylesterase (CES) 1 and quantified using a CES specific activity assay kit (Abcam, Cambridge, UK: product number: ab109717) following the manufacture protocol. at 37 °C and spun 250 RPM for 14 days. Two control conditios were used: (i) polymer rods incubated with 1 mL of PBS, and; (ii) polymer rods incubated in 1 mL of PBS containing 1 mM benzil (CES1 inhibitor), 0.1% DMSO, 4% MeOH and 125 µg/mL of microsome. 250 µL samples were taken at 4 hours, 1 day, 2 days, 3 days, 7 days, 10 days and 14 days. In order to maintain sink conditions, after each sampling time point, 250 µL of fresh microsome-containing PBS, or the control solutions ((i) neat PBS, or (ii) PBS containing benzil+microsome) was added to each polymer rod samples respectively. The FTC concentration within all samples were quantified using an adapted, previously validated liquid chromatography mass spectrometry (LCMS) method.

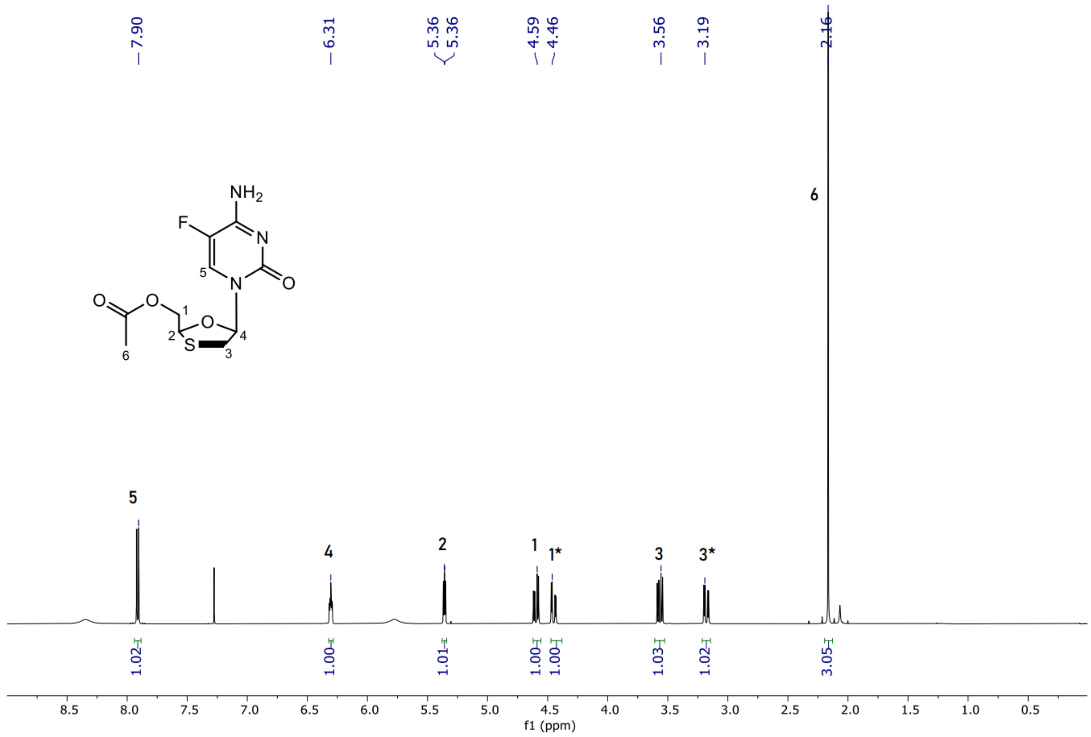

*Figure S1: ^{1}H NMR ($CDCl_3$, 500 MHz) of mono-acetylated FTC product **1.***

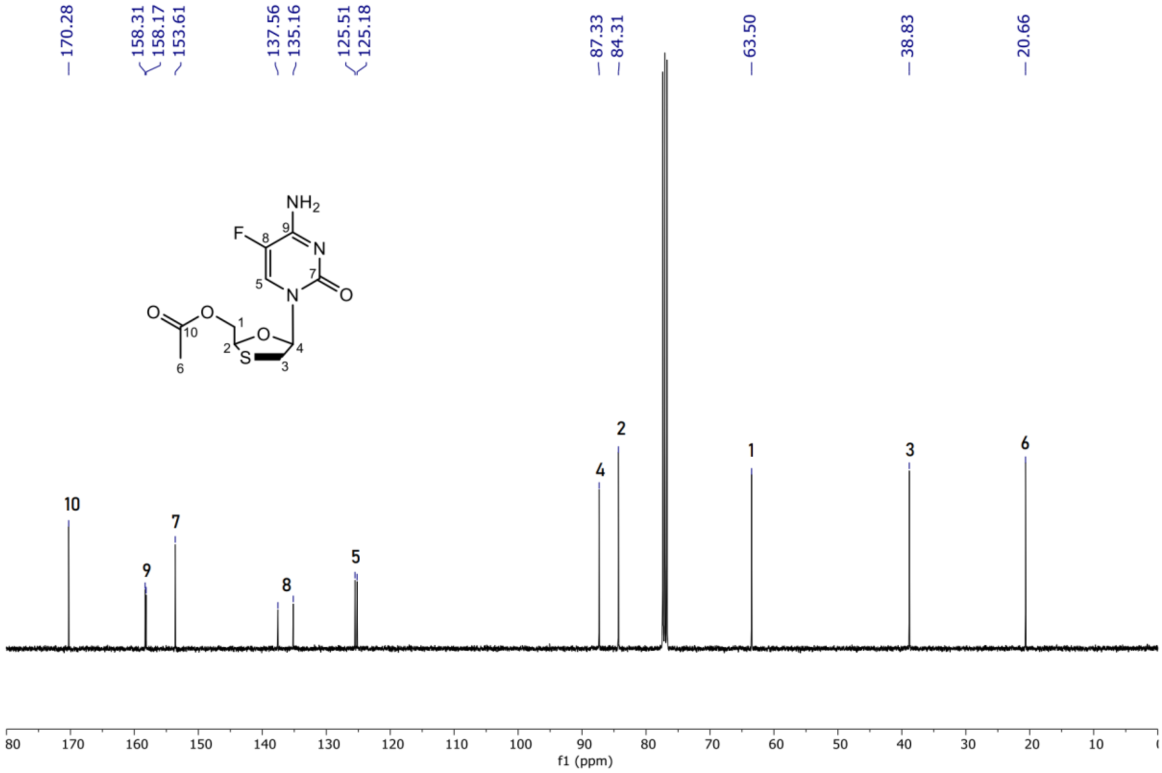

*Figure S2: ^{13}C NMR ($CDCl_3$, 100 MHz) of mono-acetylated FTC product **1.***

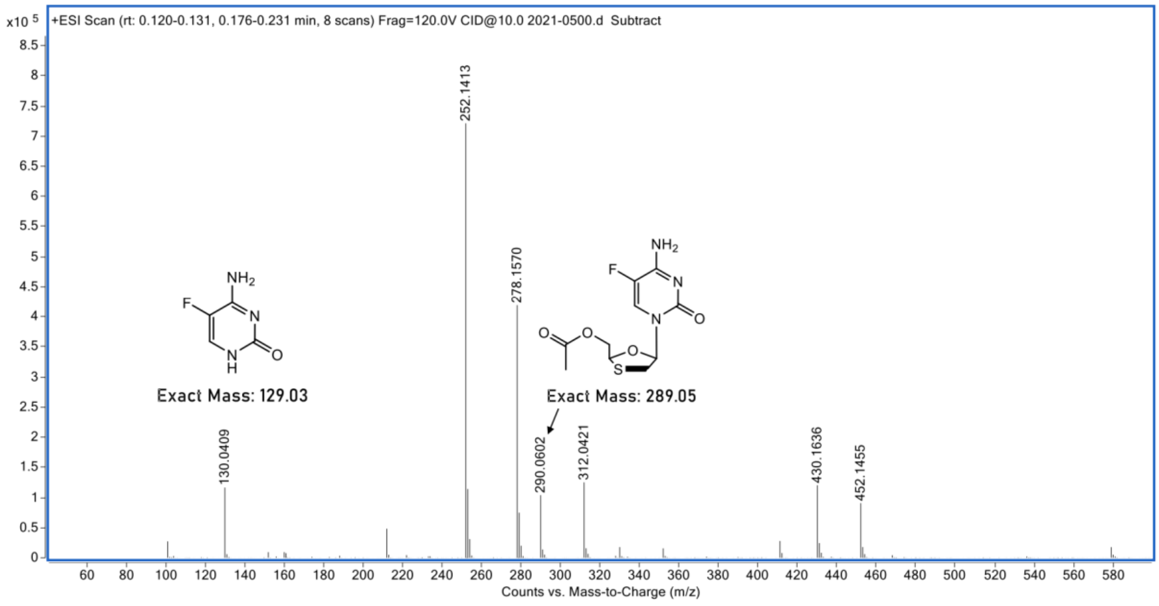

*Figure S3: ESI-MS spectrum of mono-acetylated FTC product **1** (positive ion mode).*

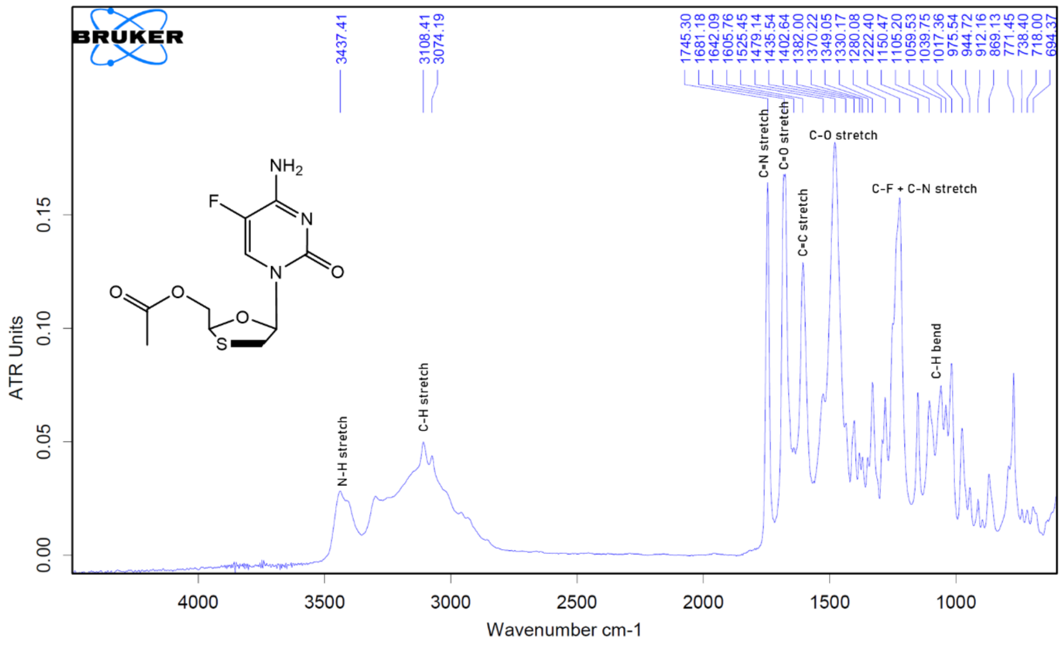

*Figure S4: FTIR spectrum of mono-acetylated FTC product **1**.*

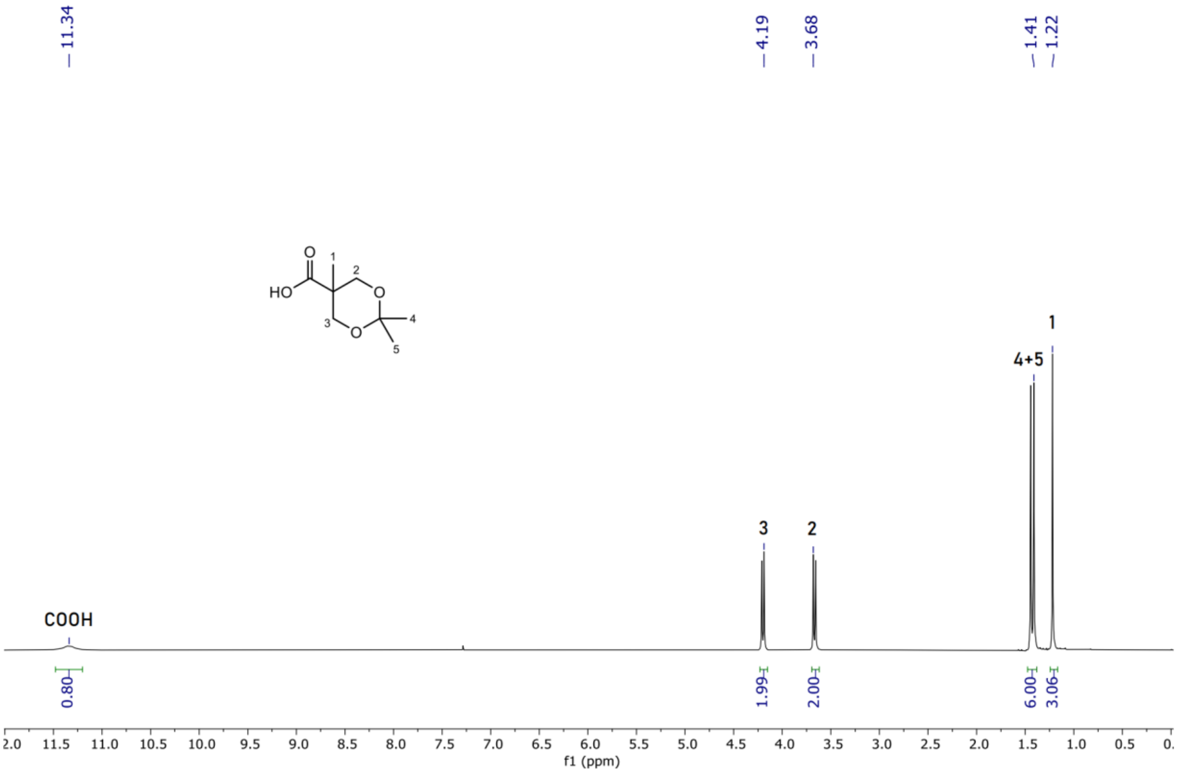

*Figure S5: ^{1}H NMR ($CDCl_3$, 500 MHz) of acetonide-protected BMPA **3**.*

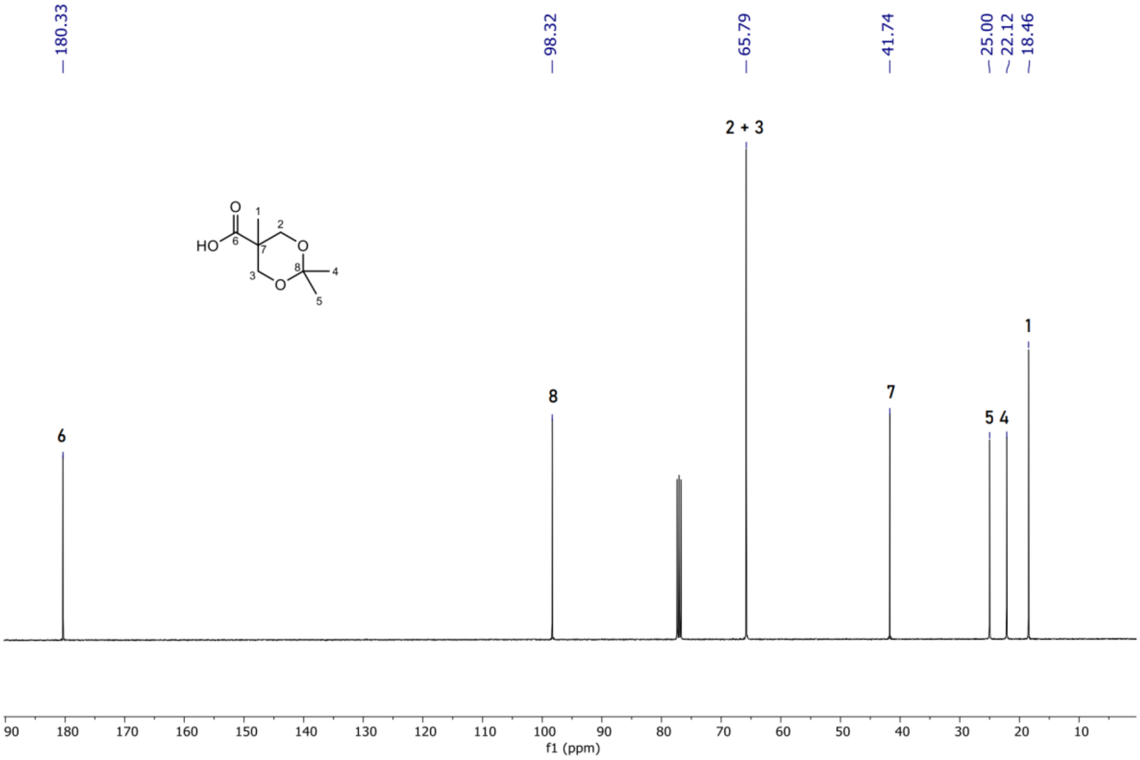

*Figure S6: ^{13}C NMR ($CDCl_3$, 100 MHz) of acetonide-protected BMPA **3**.*

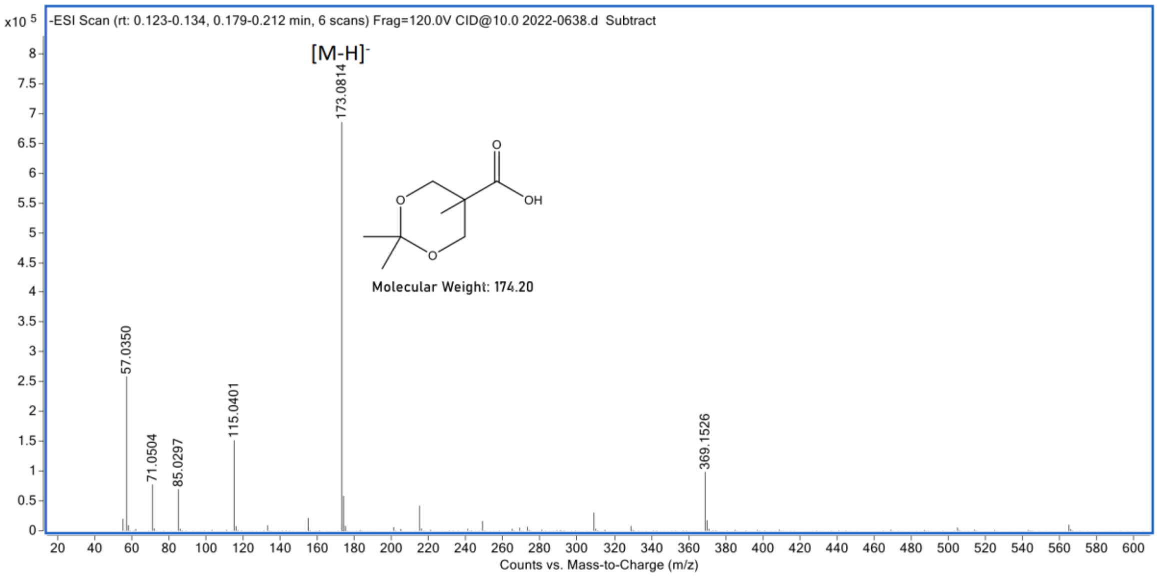

*Figure S7: ESI-MS of acetonide-protected BMPA **3** (negative ion mode).*

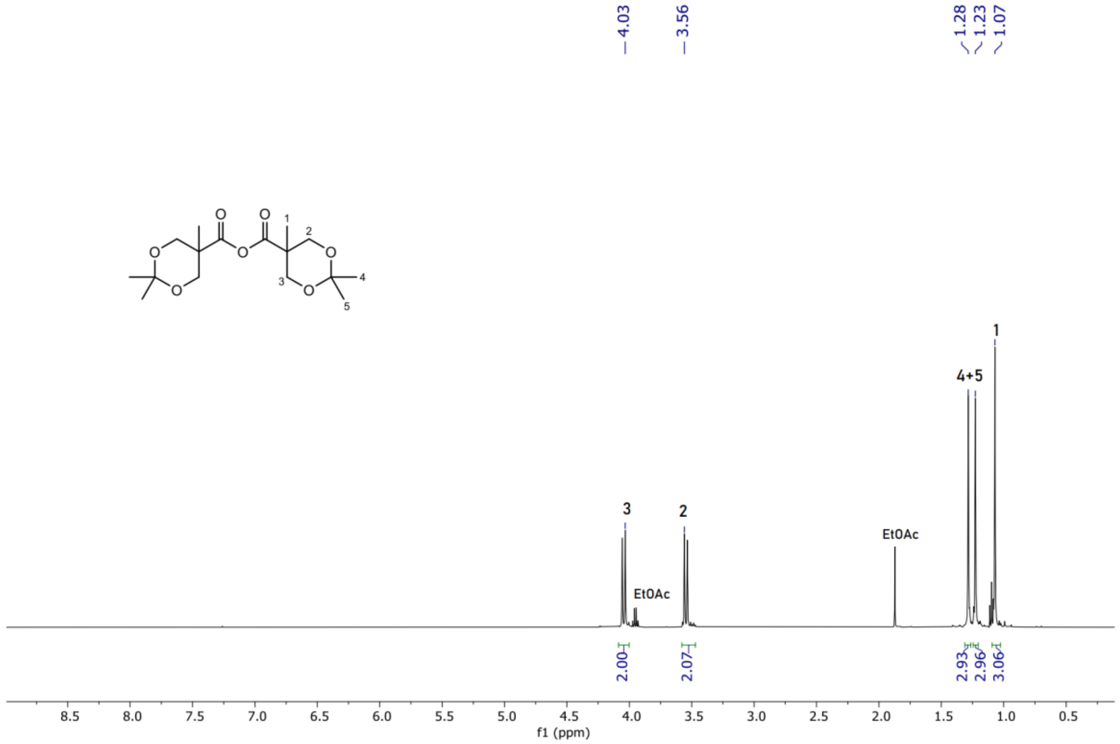

*Figure S8: ^{1}H NMR ($CDCl_3$, 500 MHZ) of symmetrical BMPA anhydride **4**.*

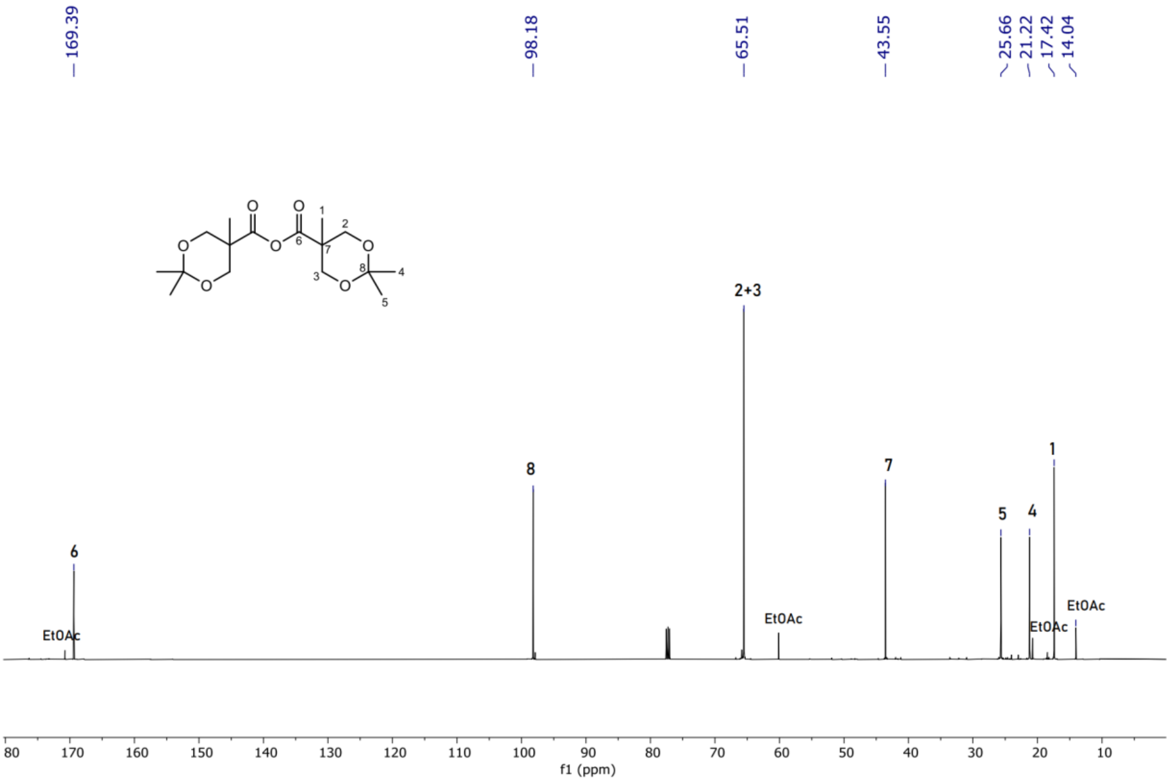

Figure S9: ^{13}C NMR ($CDCl_3$, 100 MHz) of symmetrical BMPA anhydride ***4****.*

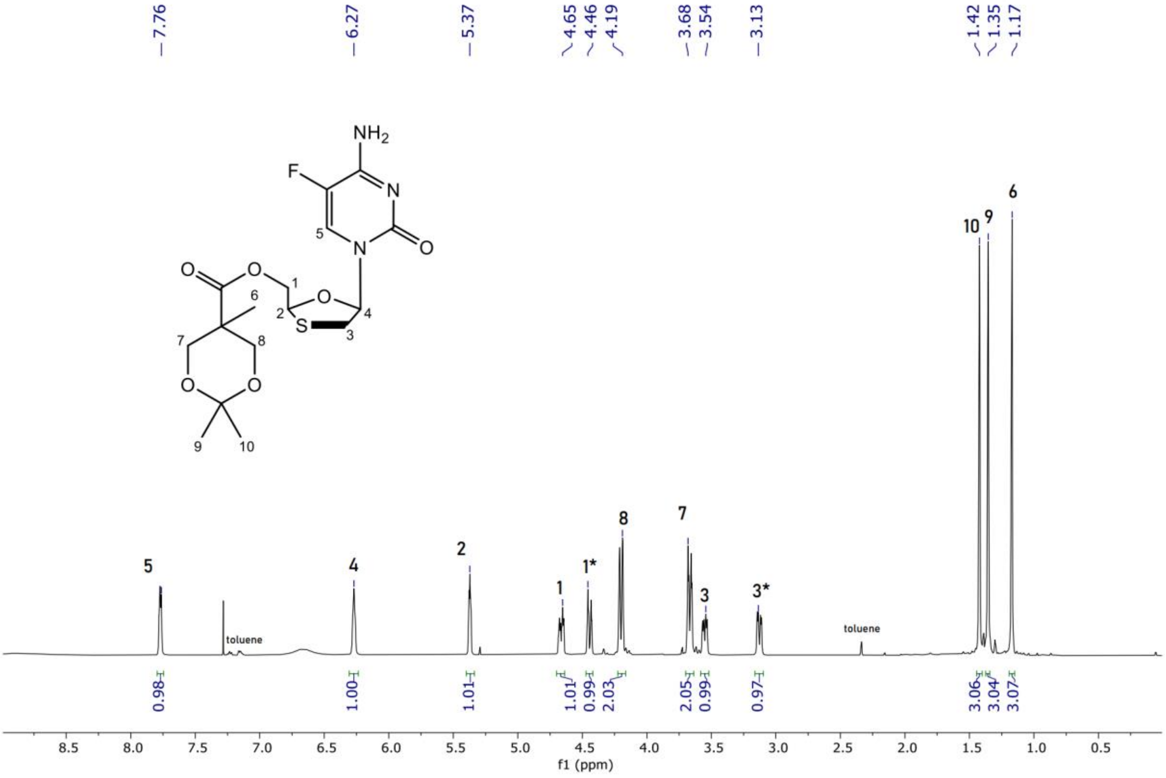

Figure S10: ^{1}H NMR ($CDCl_3$, 500 MHz) of BMPA-FTC mono-ester ***5****.*

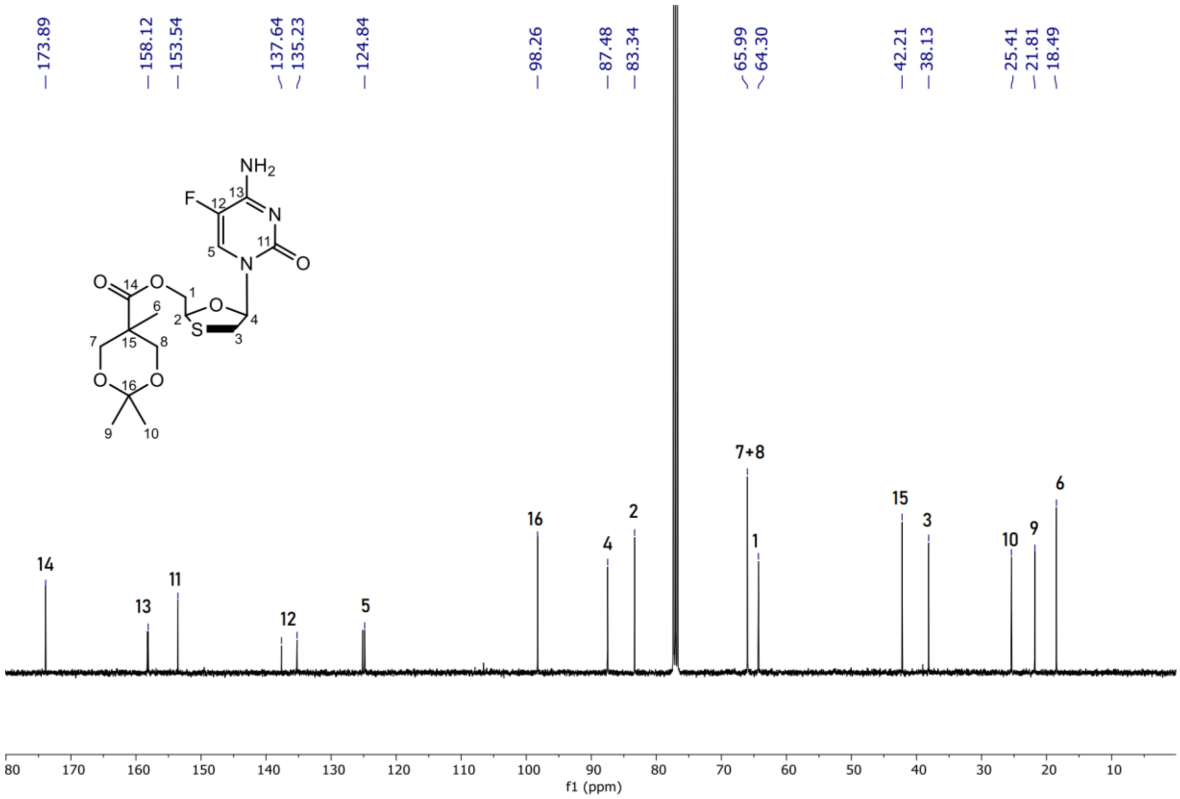

*Figure S11: ^{13}C NMR ($CDCl_3$, 100 MHz) of BMPA-FTC mono-ester **5.***

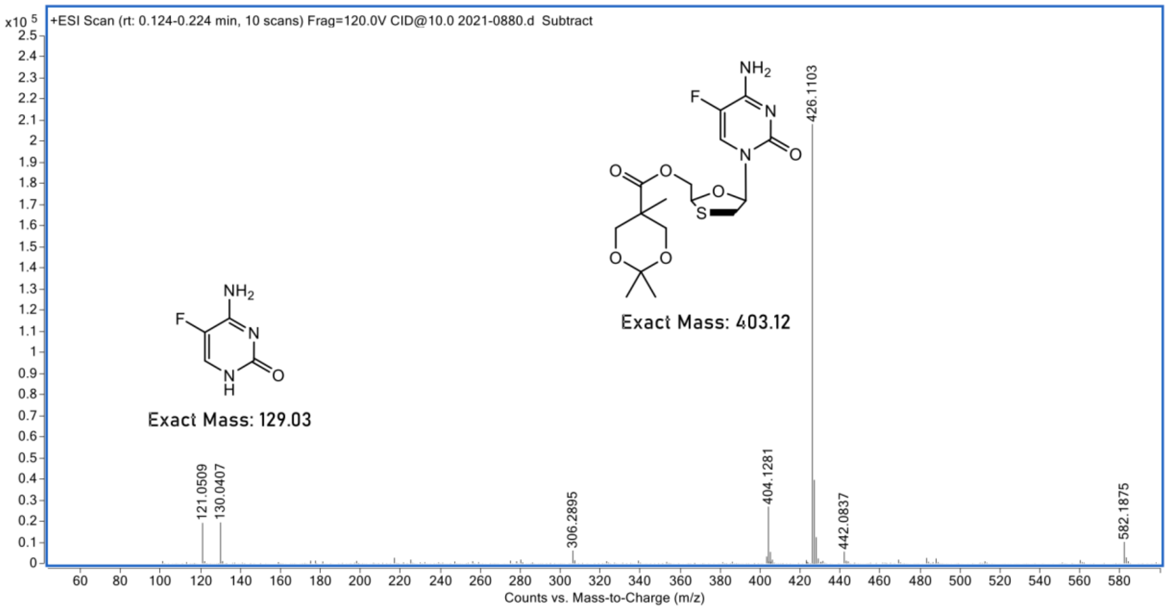

*Figure S12: ESI-MS spectrum of BMPA-FTC mono-ester **5** (positive ion mode).*

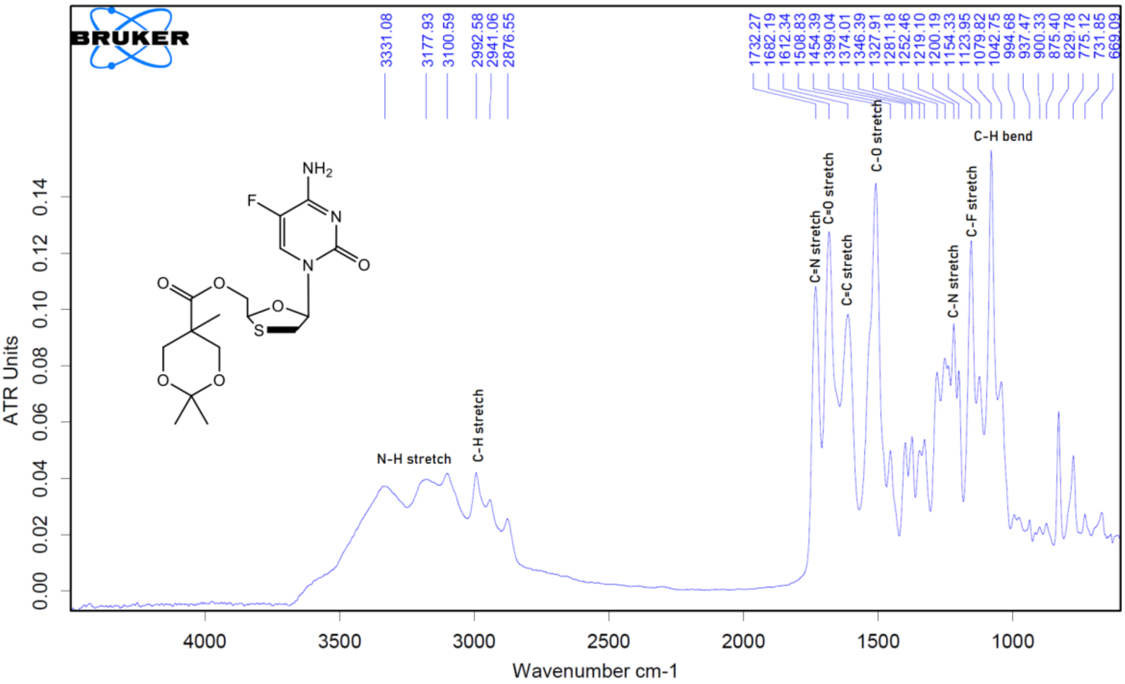

*Figure S13: FTIR spectrum of BMPA-FTC mono-ester **5**.*

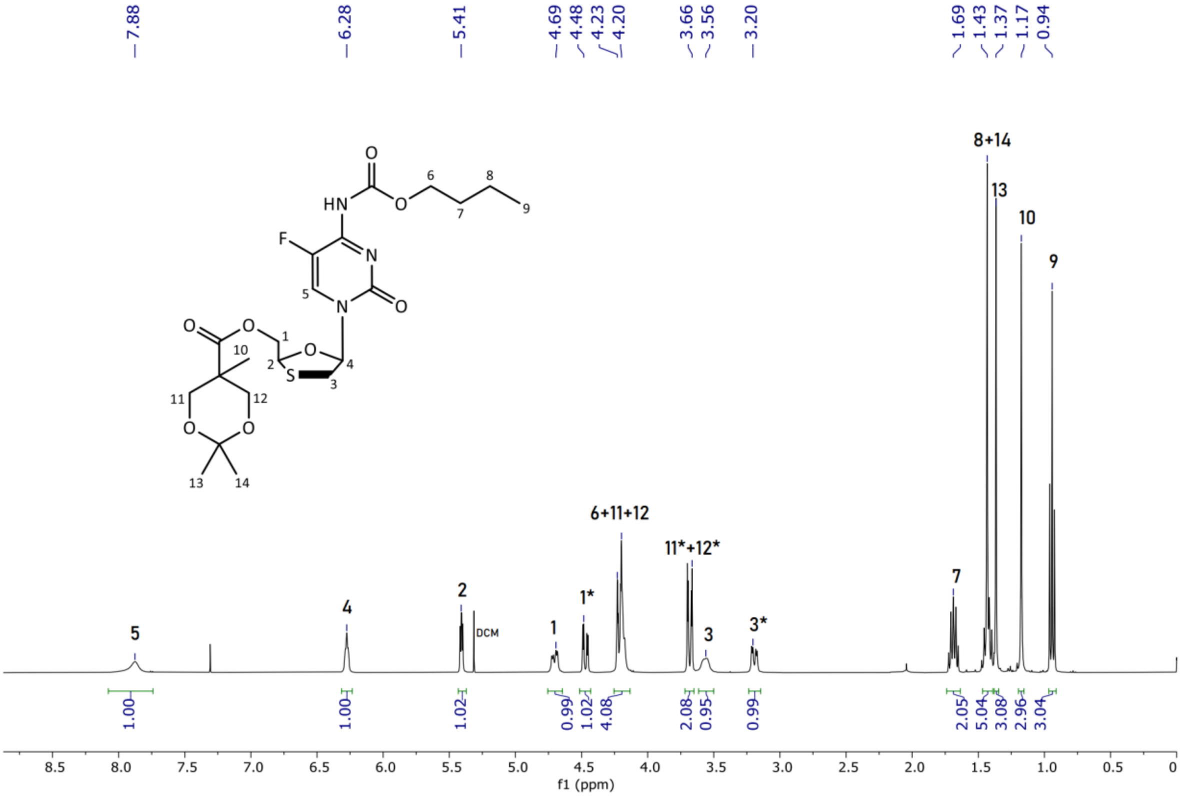

*Figure S14: ^{1}H NMR ($CDCl_3$, 500 MHz) of n-butyl-carbamate, BMPA-ester product **6a**.*

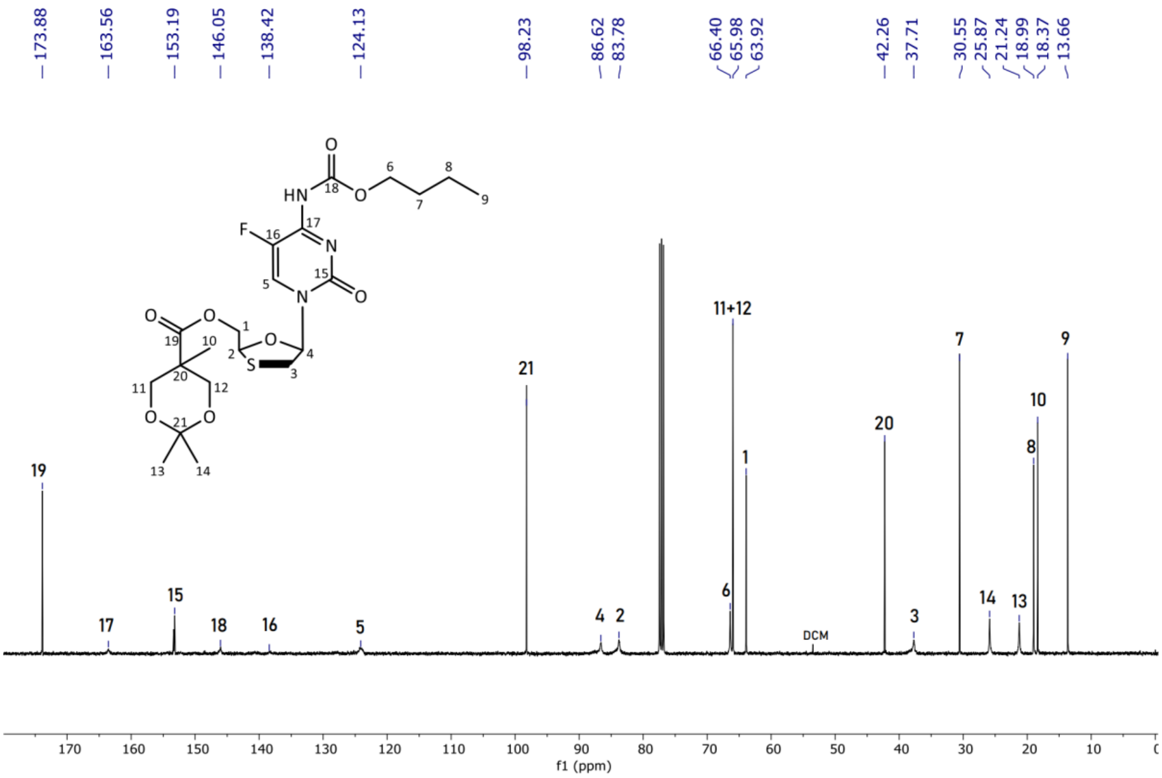

Figure S15: ^{13}C NMR ($CDCl_3$, 100 MHz) of n-butyl-carbamate, BMPA-ester product ***6a.***

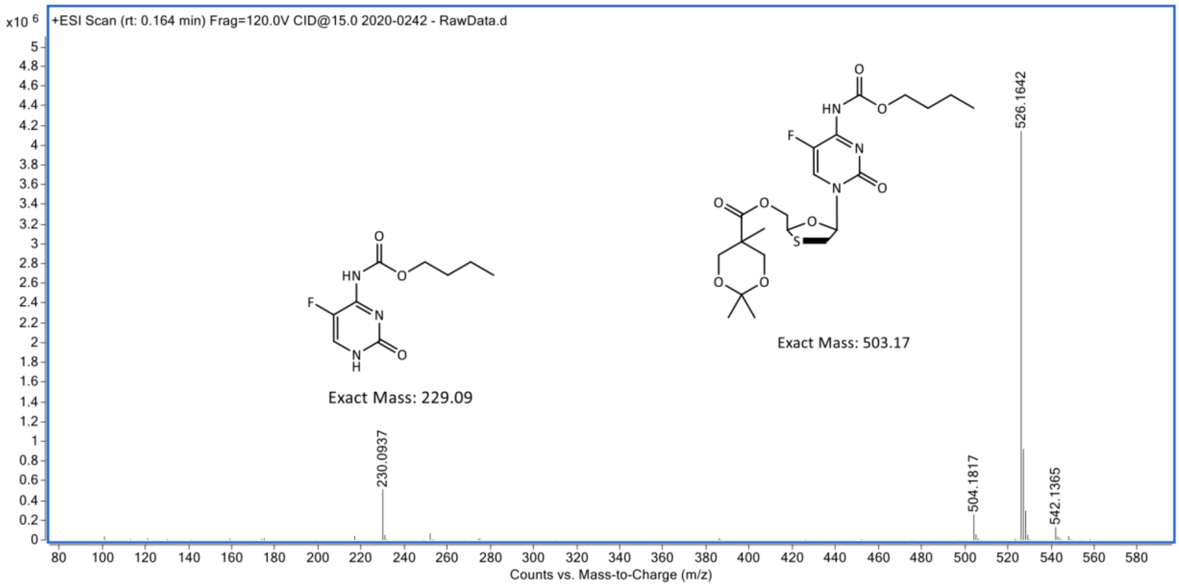

Figure S16: EST-MS spectrum of n-butyl-carbamate, BMPA-ester product ***6a*** *(positive ion mode).*

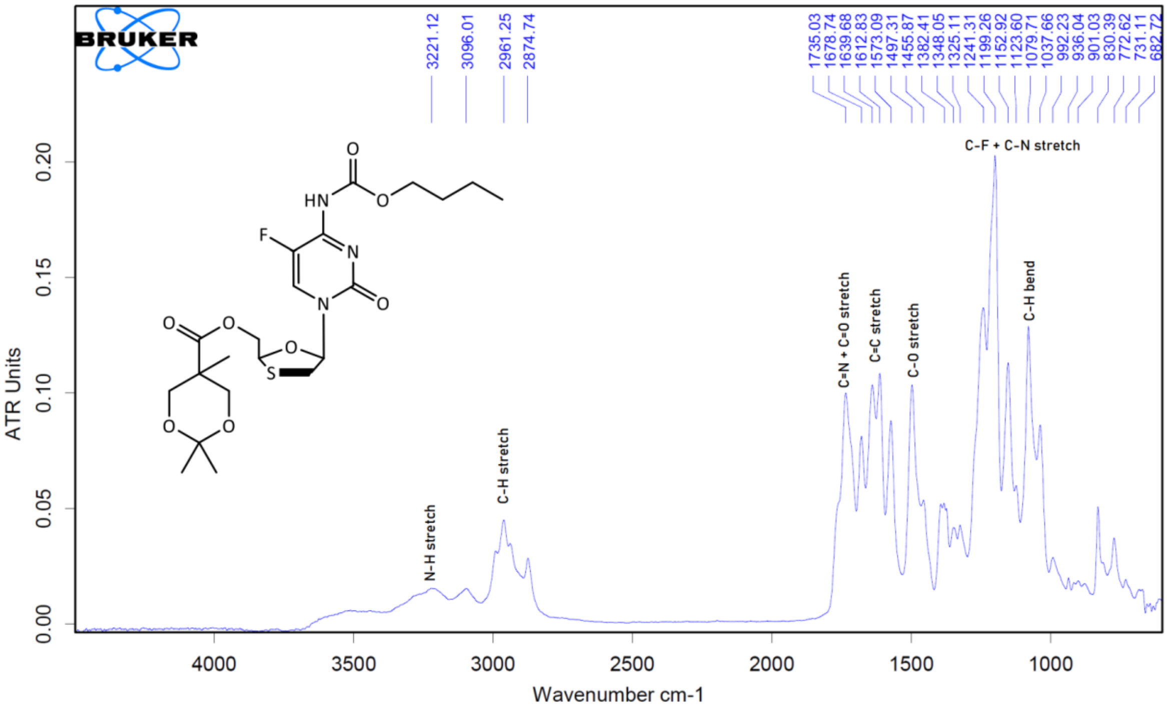

Figure S17: FTIR spectrum of n-butyl-carbamate, BMPA-ester product ***6a.***

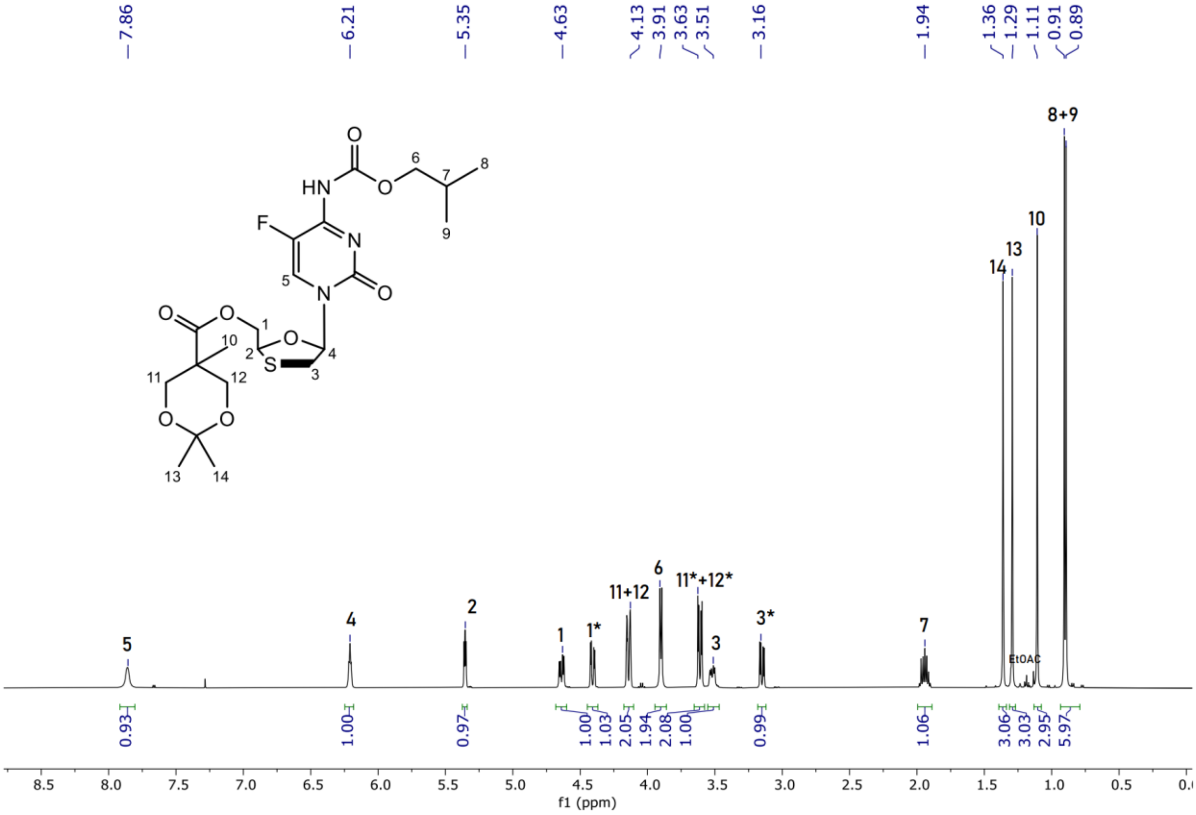

Figure S18: ^{1}H NMR ($CDCl_3$, 500 MHz) of i-butyl-carbamate, BMPA-ester product ***6b****.*

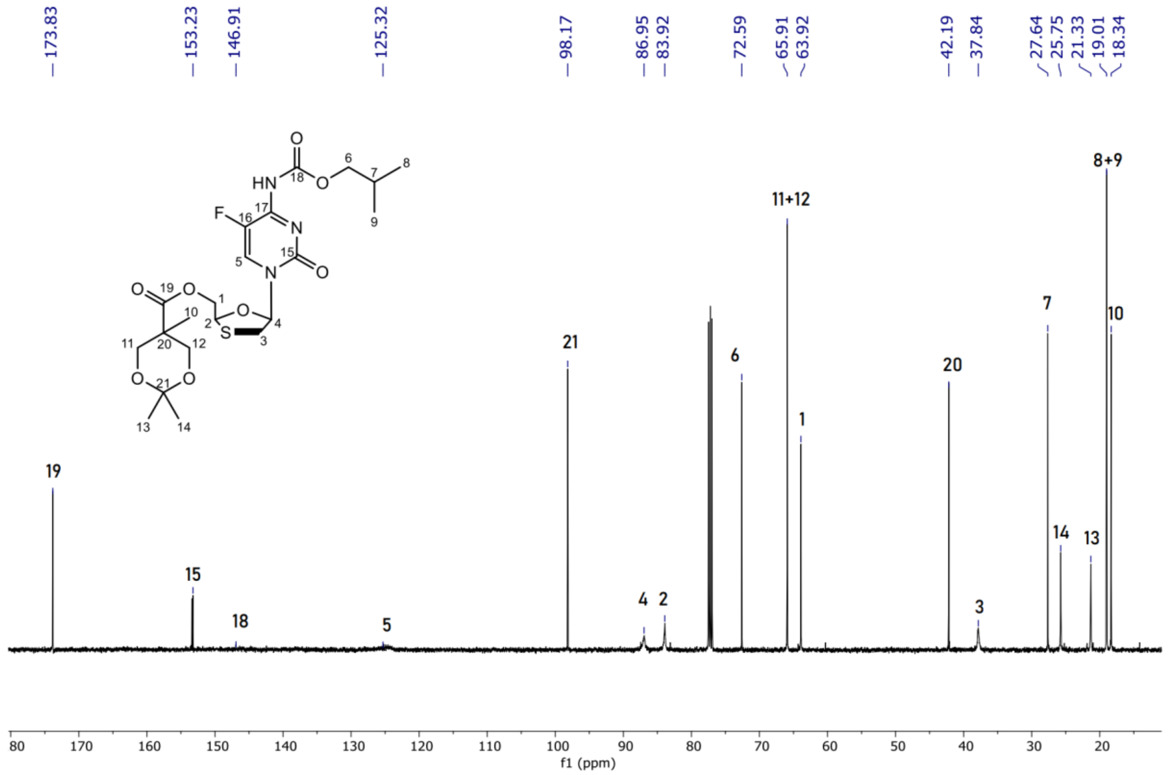

*Figure S19: ^{13}C NMR ($CDCl_3$, 100 MHz) of i-butyl-carbamate, BMPA-ester product **6b**.*

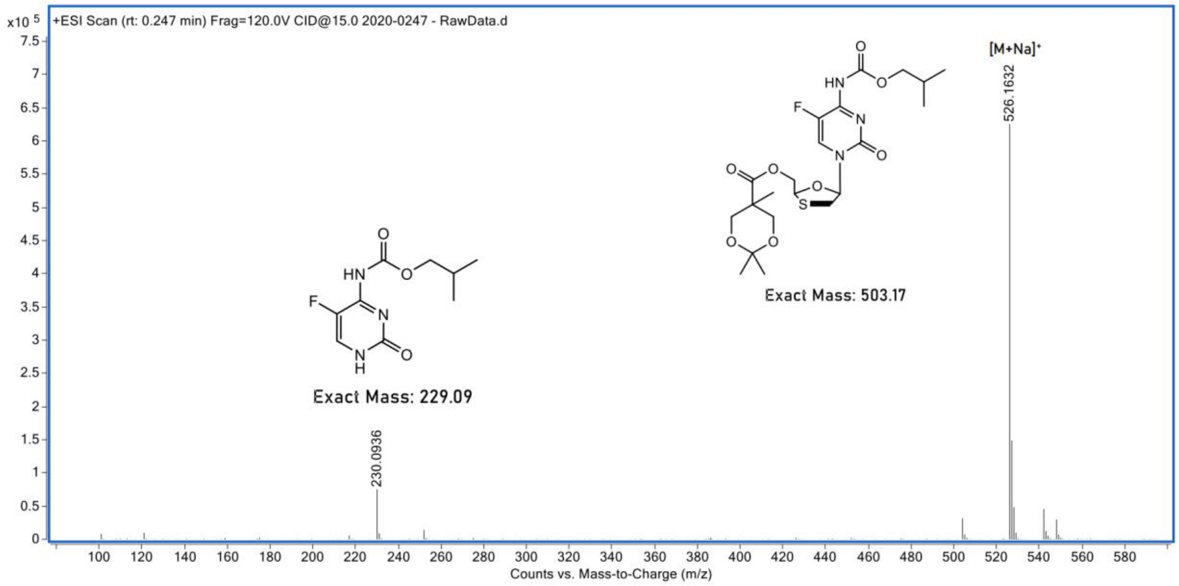

*Figure S20: ESI-MS spectrum of i-butyl-carbamate, BMPA-ester product **6b** (positive ion mode)*

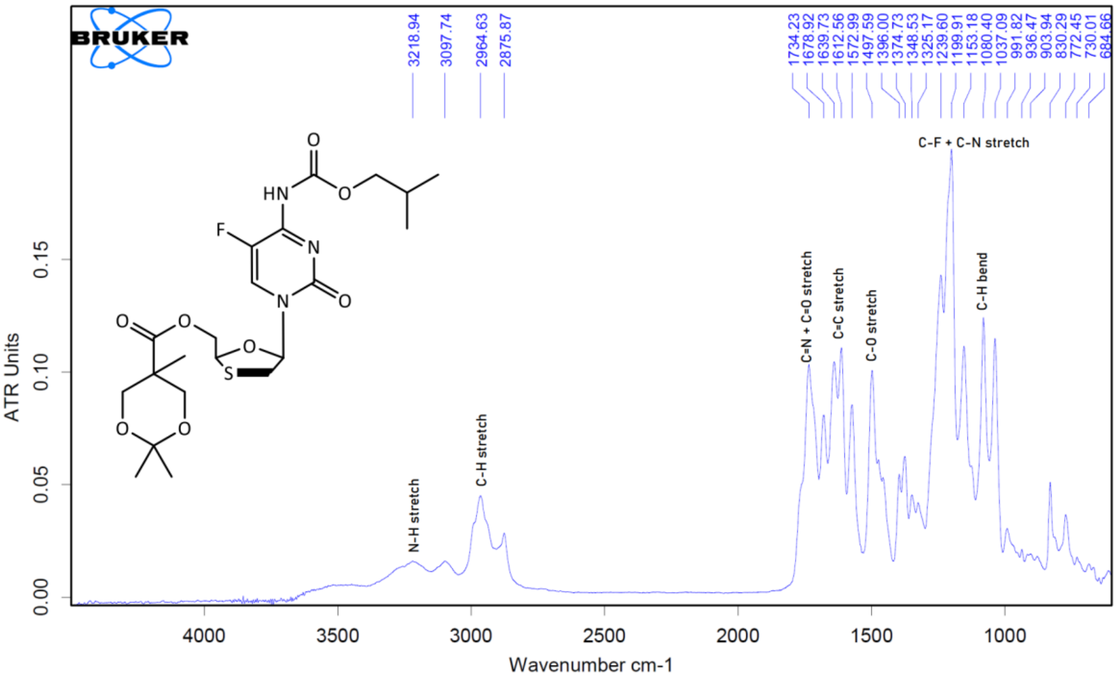

*Figure S21: FTIR spectrum of i-butyl-carbamate, BMPA-ester product **6b**.*

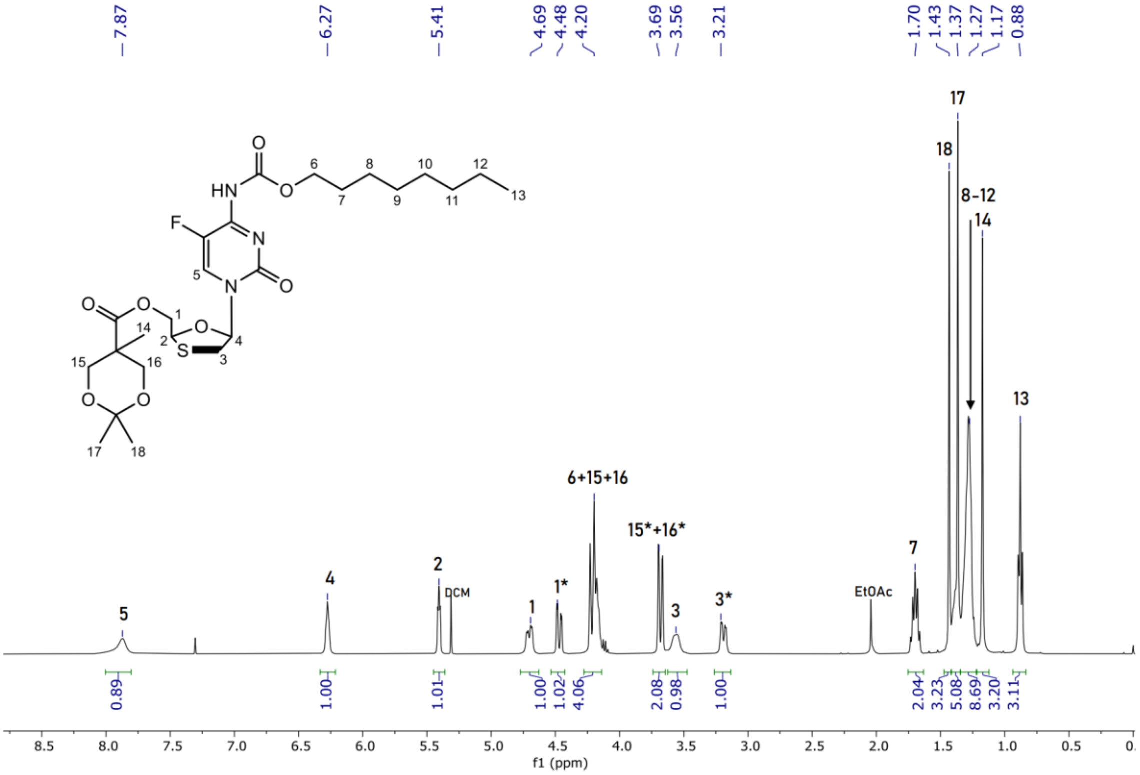

*Figure S22: ^{1}H NMR ($CDCl_3$, 500 MHz) of n-octyl-carbamate, BMPA-ester product **6c**.*

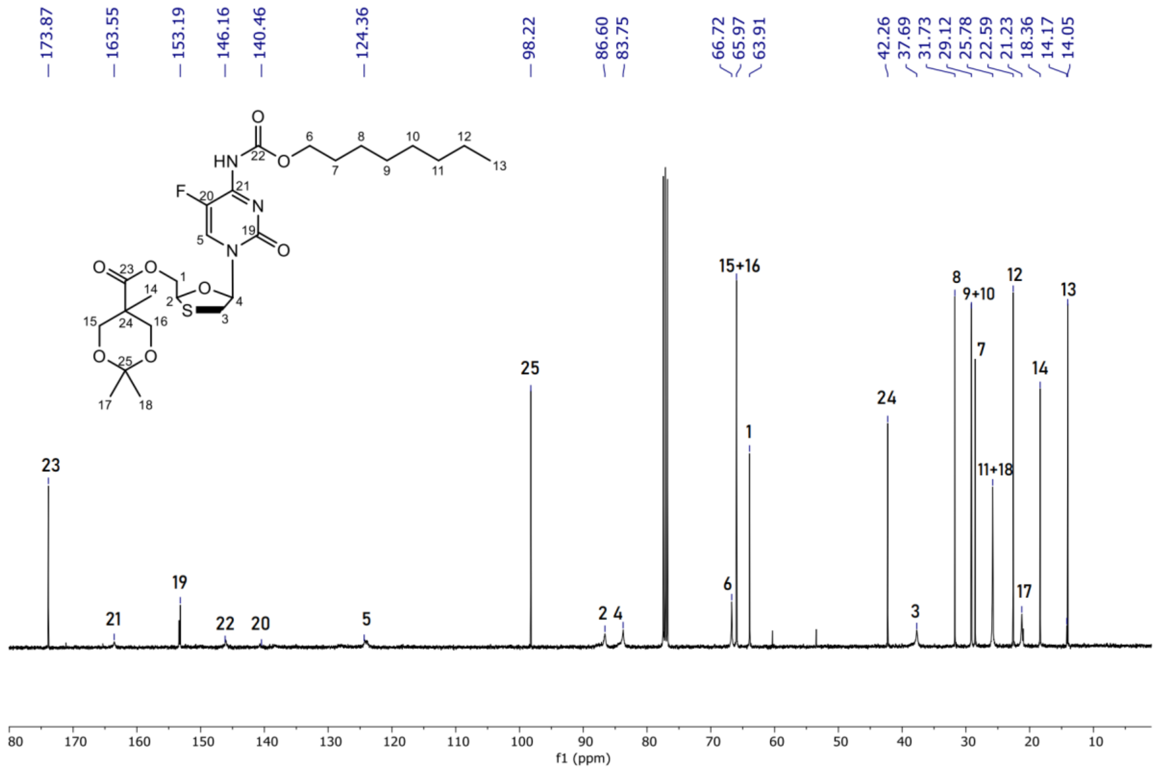

*Figure S23: ^{13}C NMR ($CDCl_3$, 100 MHz) of n-octyl-carbamate, BMPA-ester product **6c**.*

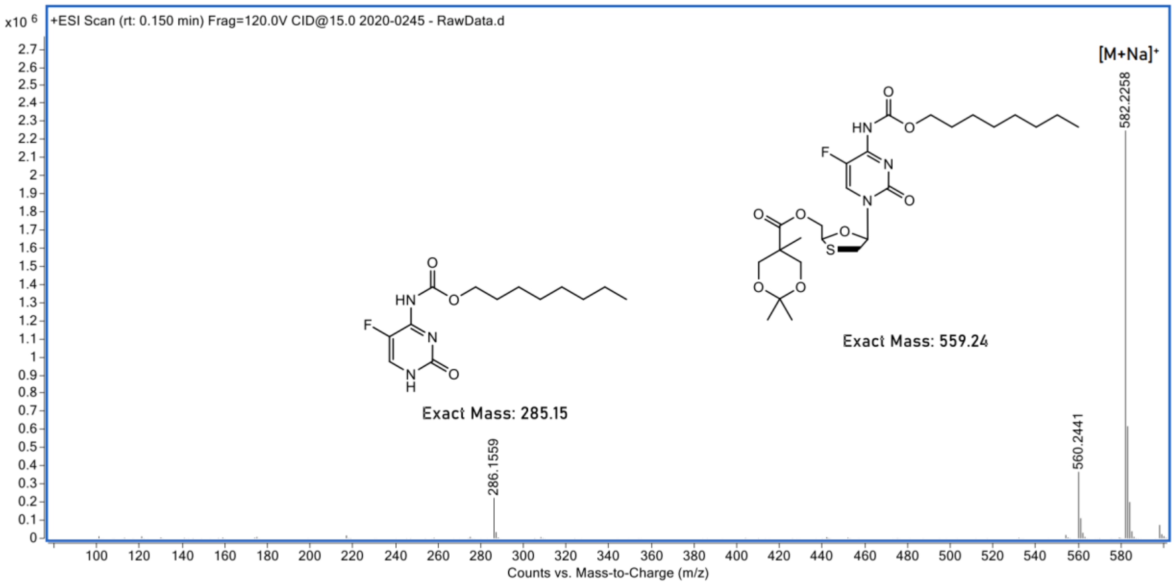

*Figure S24: ESI-MS spectrum of n-octyl-carbamate, BMPA-ester product **6c** (positive ion mode)*

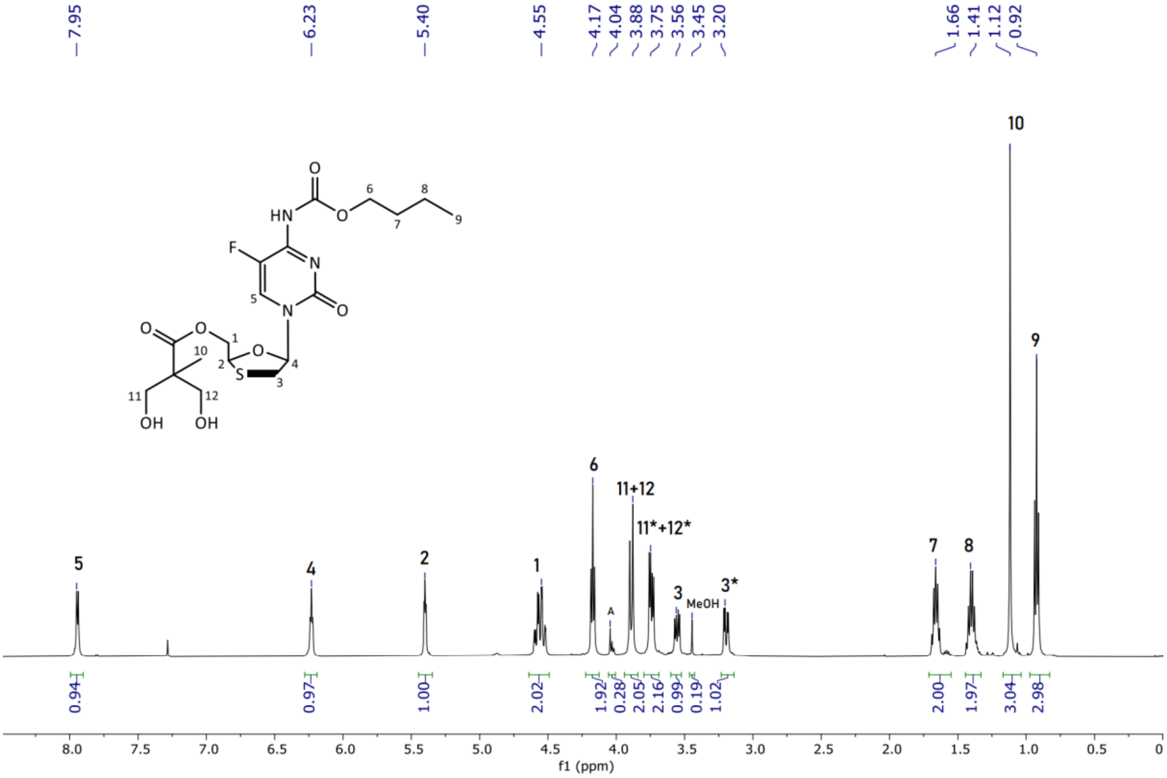

*Figure S25: ^{1}H NMR ($CDCl_3$, 500 MHz) of n-butyl-carbamate diol monomer **7a**.*

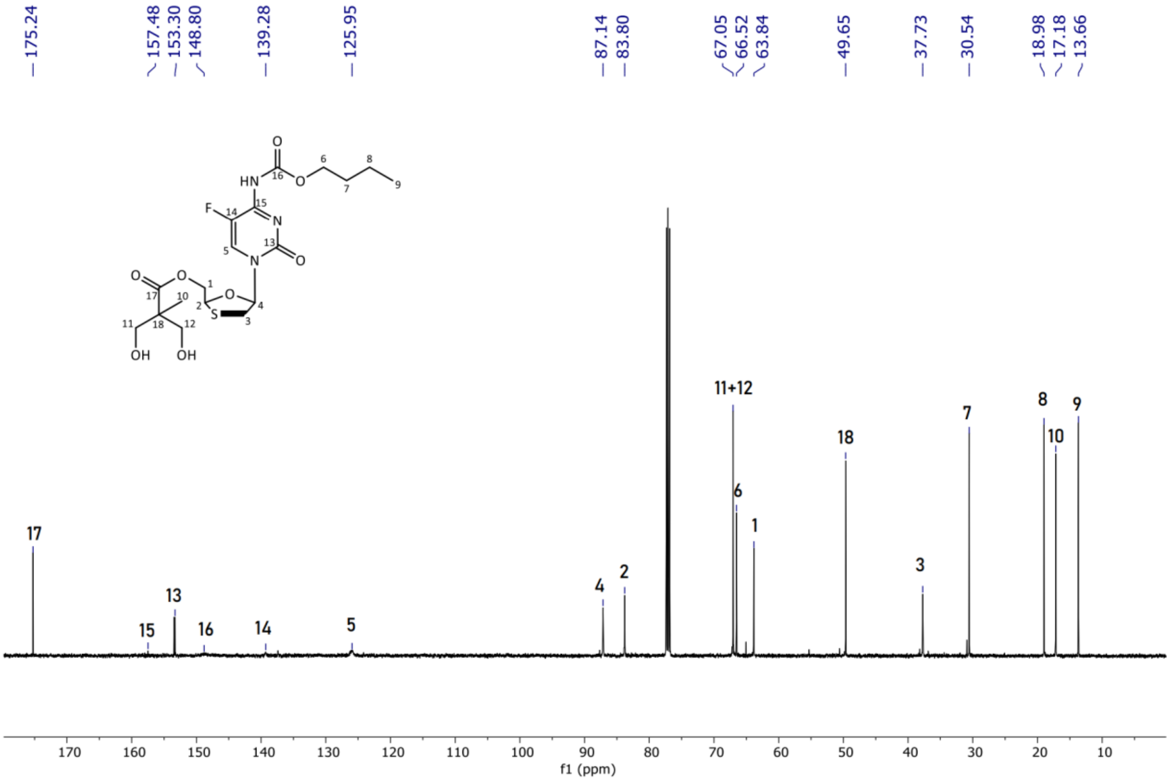

*Figure S26: ^{13}C NMR ($CDCl_3$, 100 MHz) of n-butyl-carbamate diol monomer **7a.***

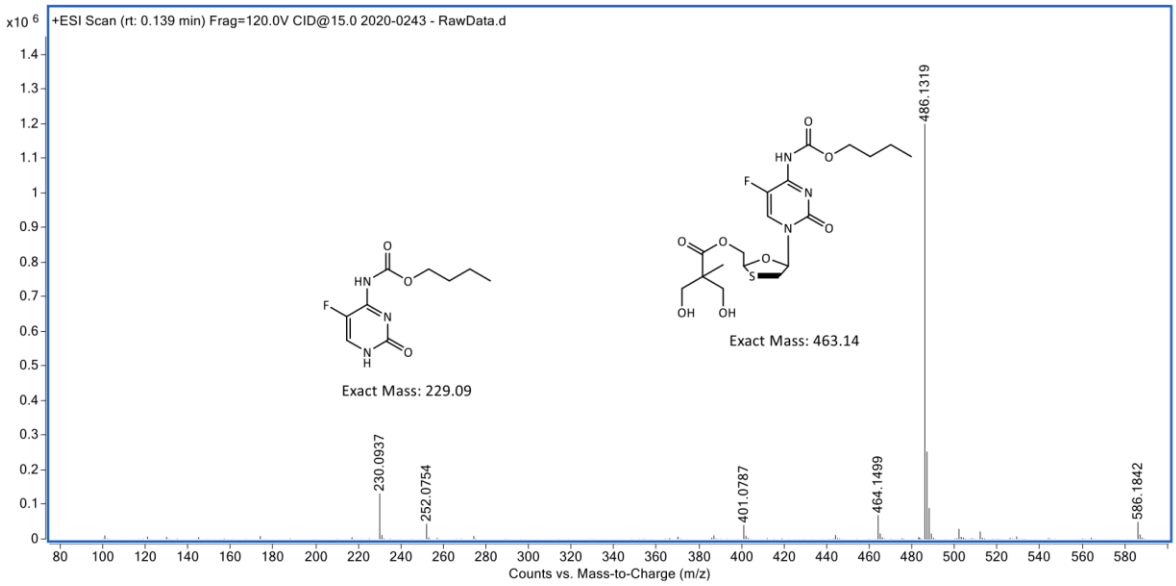

*Figure S27: ESI-MS spectrum of n-butyl-carbamate diol monomer **7a** (positive ion mode)*

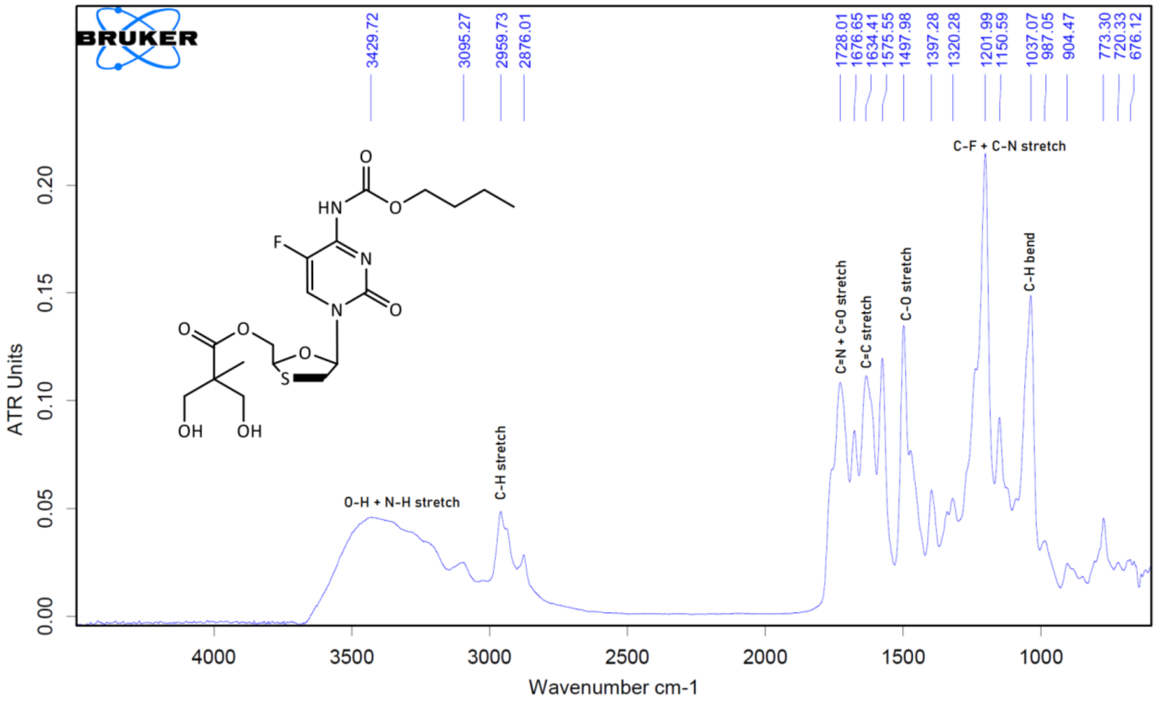

*Figure S28: FTIR spectrum of n-butyl-carbamate diol monomer **7a.***

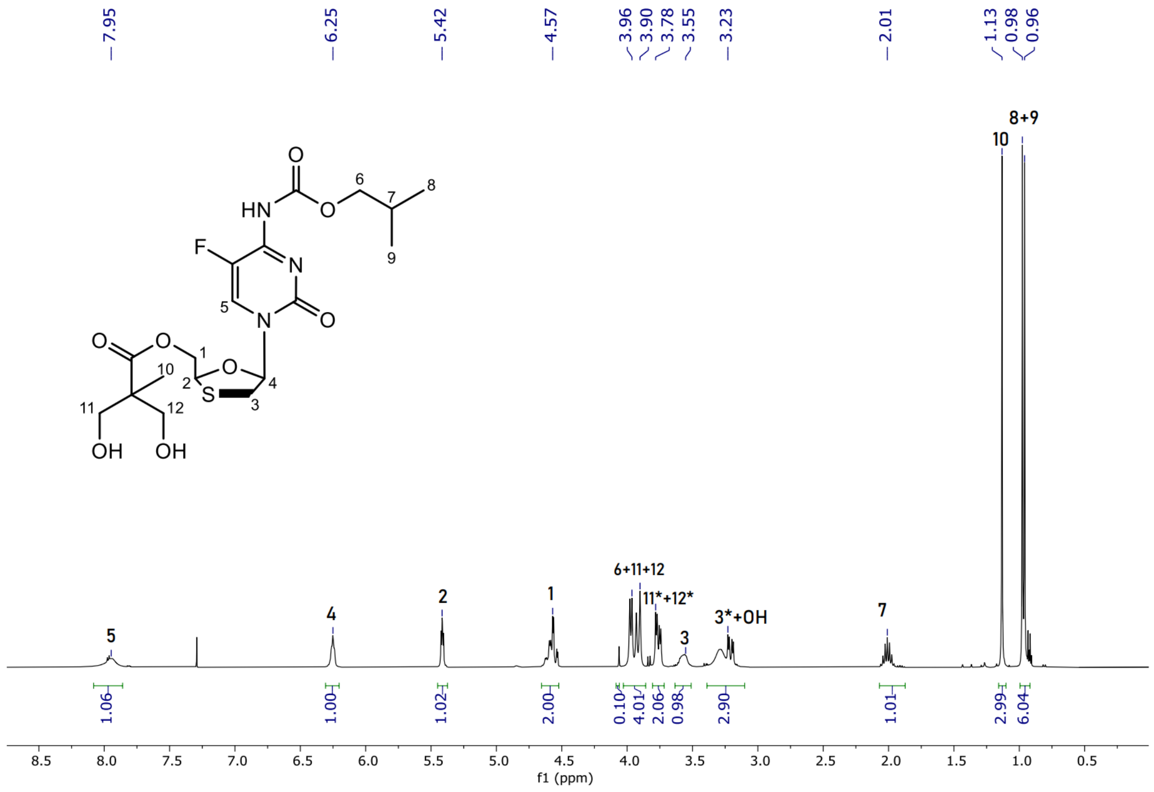

*Figure S29: ^{1}H NMR ($CDCl_3$, 500 MHz) of i-butyl-carbamate diol monomer **7b**.*

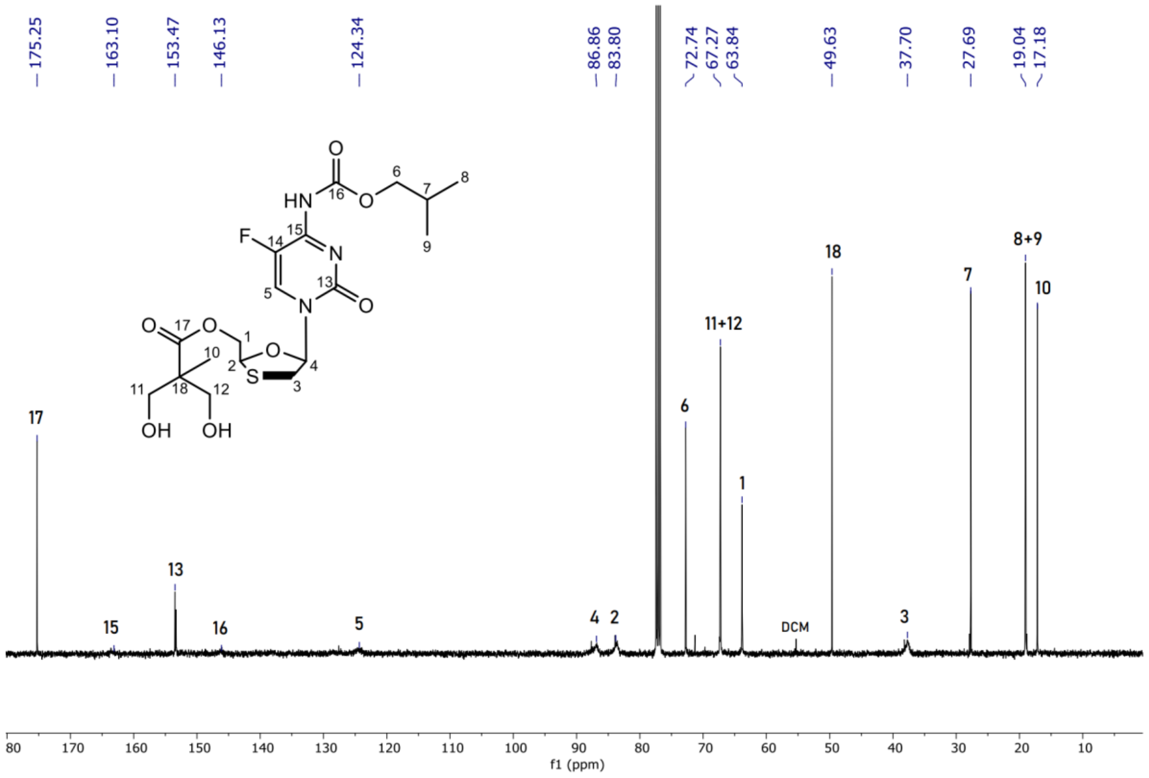

*Figure S30: ^{13}C NMR ($CDCl_3$, 100 MHz) of i-butyl-carbamate diol monomer **7b**.*

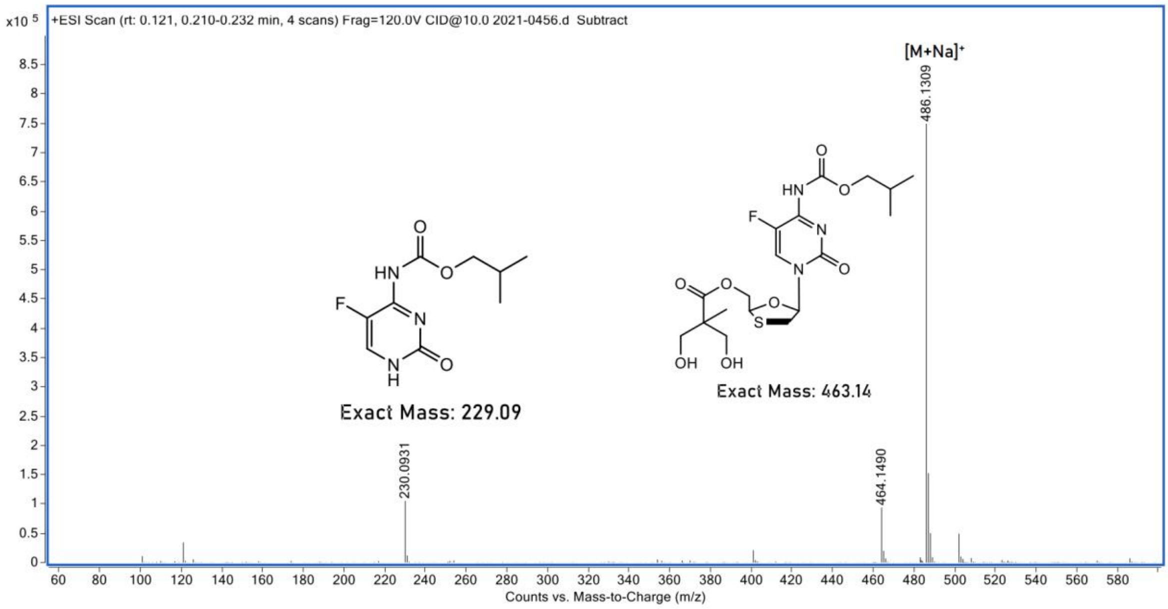

*Figure S31: ESI-MS of i-butyl-carbamate diol monomer **7b** (positive ion mode).*

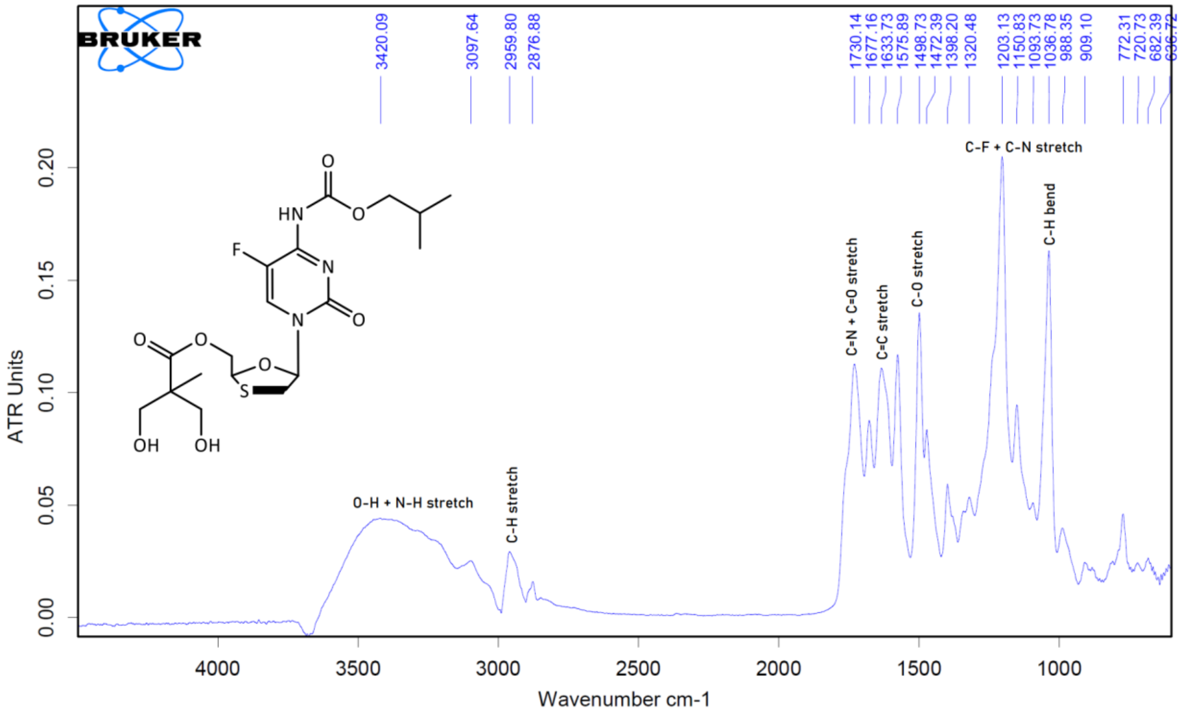

*Figure S32: FTIR of i-butyl-carbamate diol monomer **7b**.*

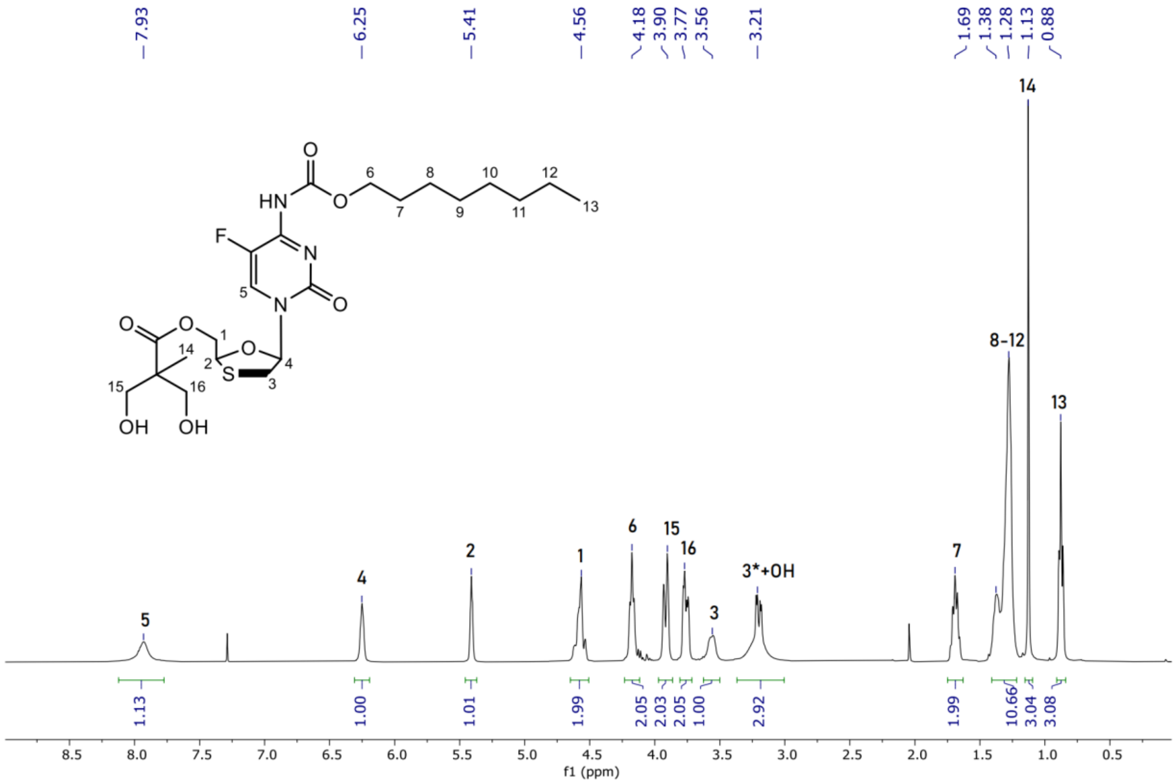

*Figure S33: ^{1}H NMR (CDCl$_3$, 500 MHz) of n-octyl -carbamate diol monomer **7c**.*

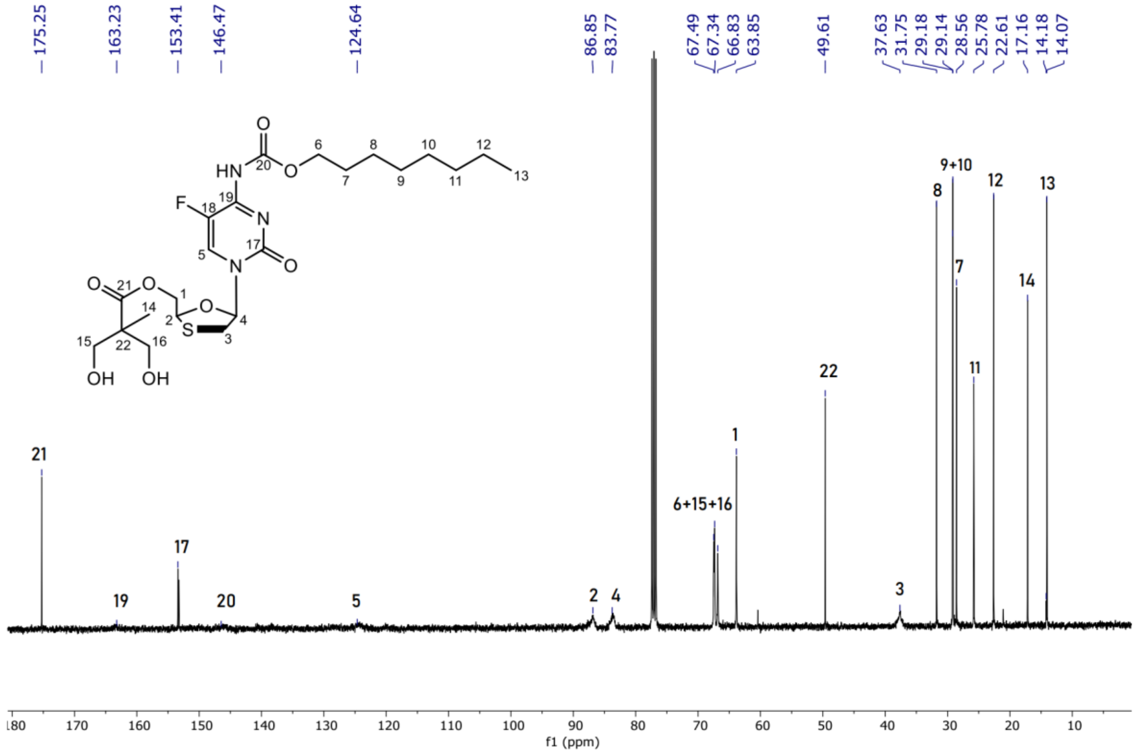

*Figure S34: ^{13}C NMR (CDCl$_3$, 100 MHz) of n-octyl-carbamate diol monomer **7c**.*

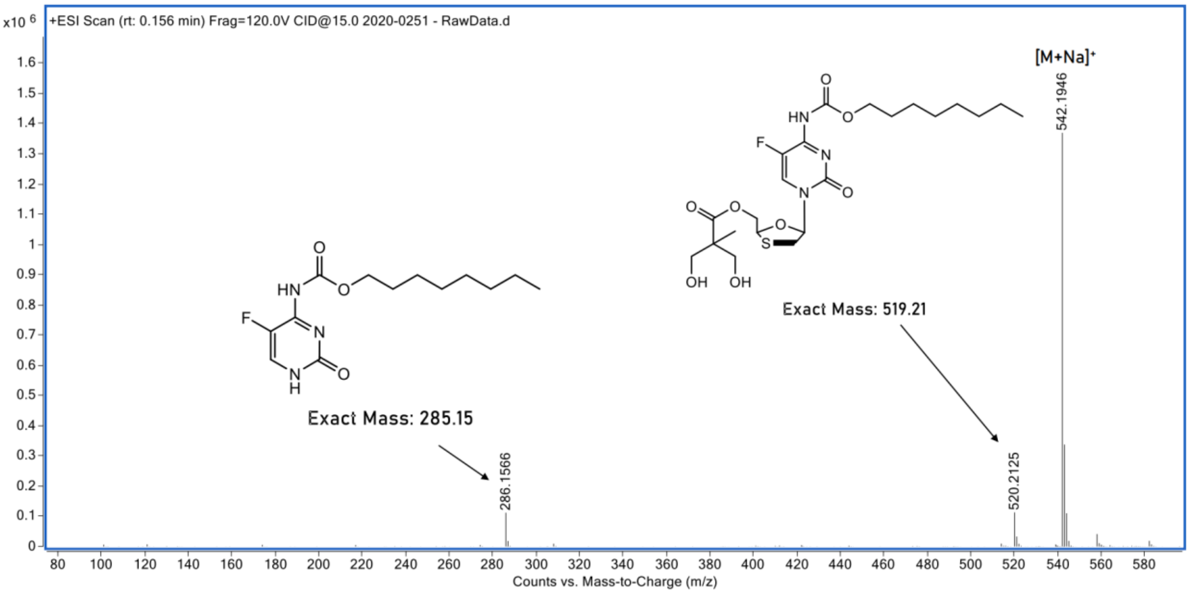

*Figure S35: ESI-MS spectrum of n-octyl-carbamate diol monomer **7c** (positive ion mode).*

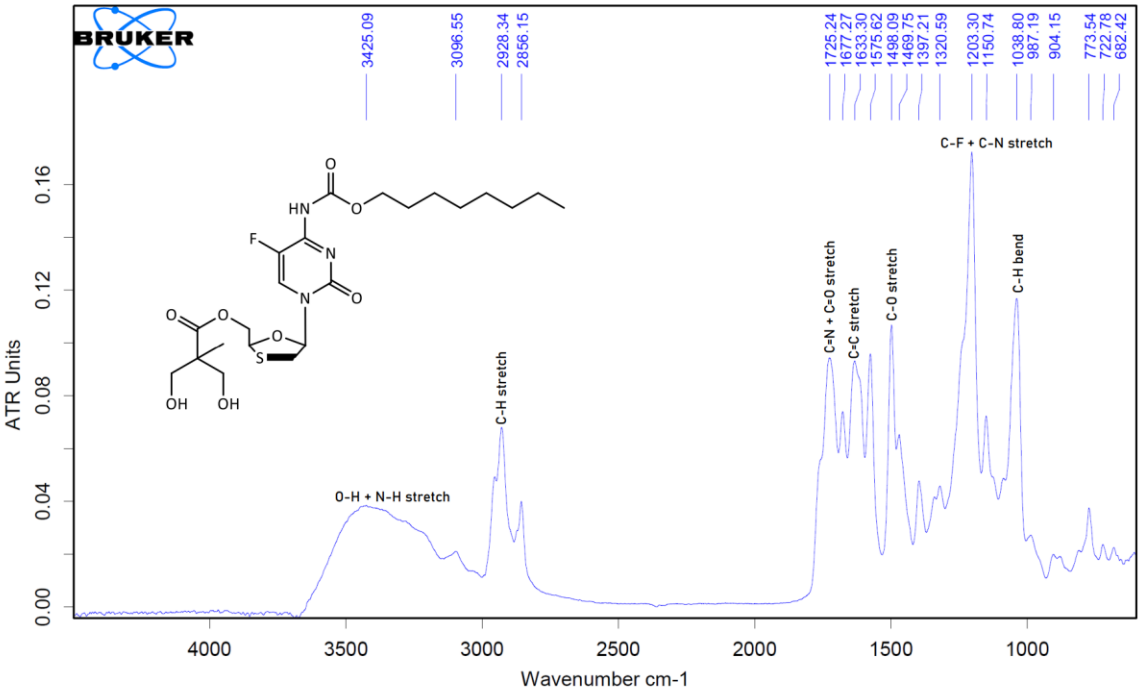

*Figure S36: FTIR spectrum of n-octyl-carbamate diol monomer **7c.***

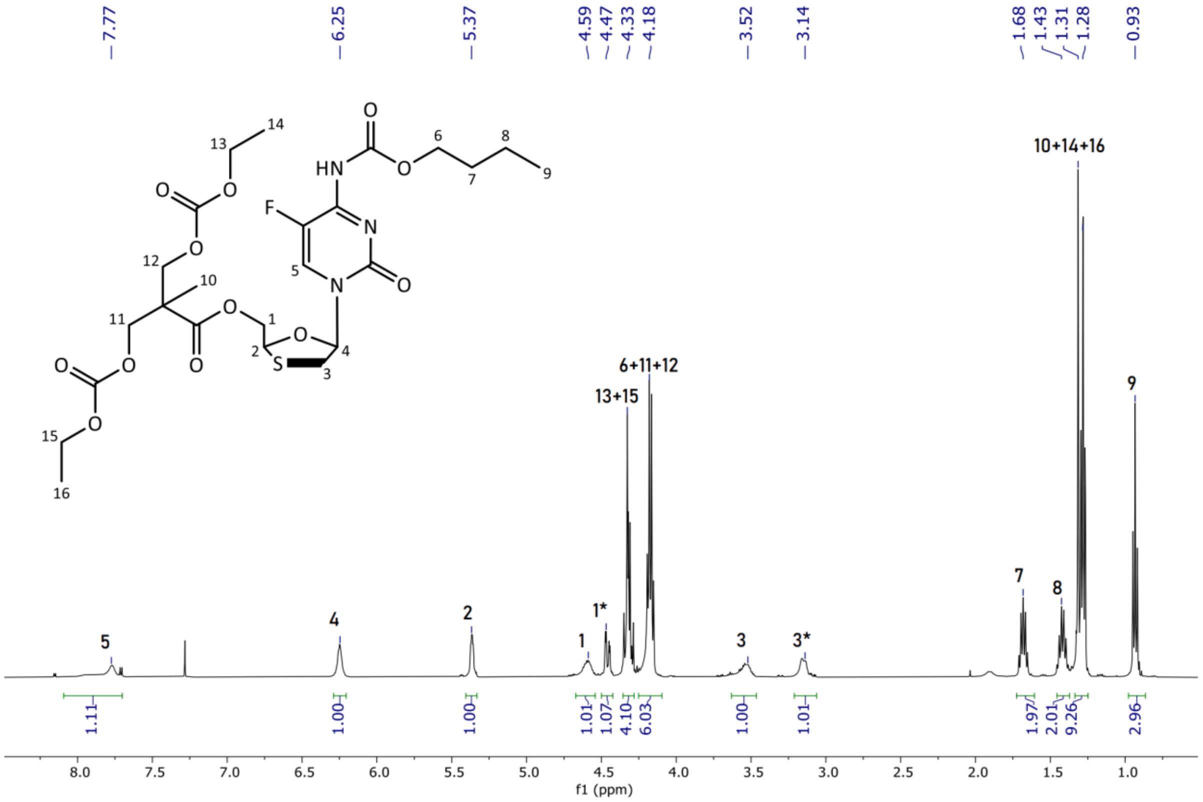

*Figure S37: ¹H NMR ($CDCl_3$, 500 MHz) of model dicarbonate compound **12**.*

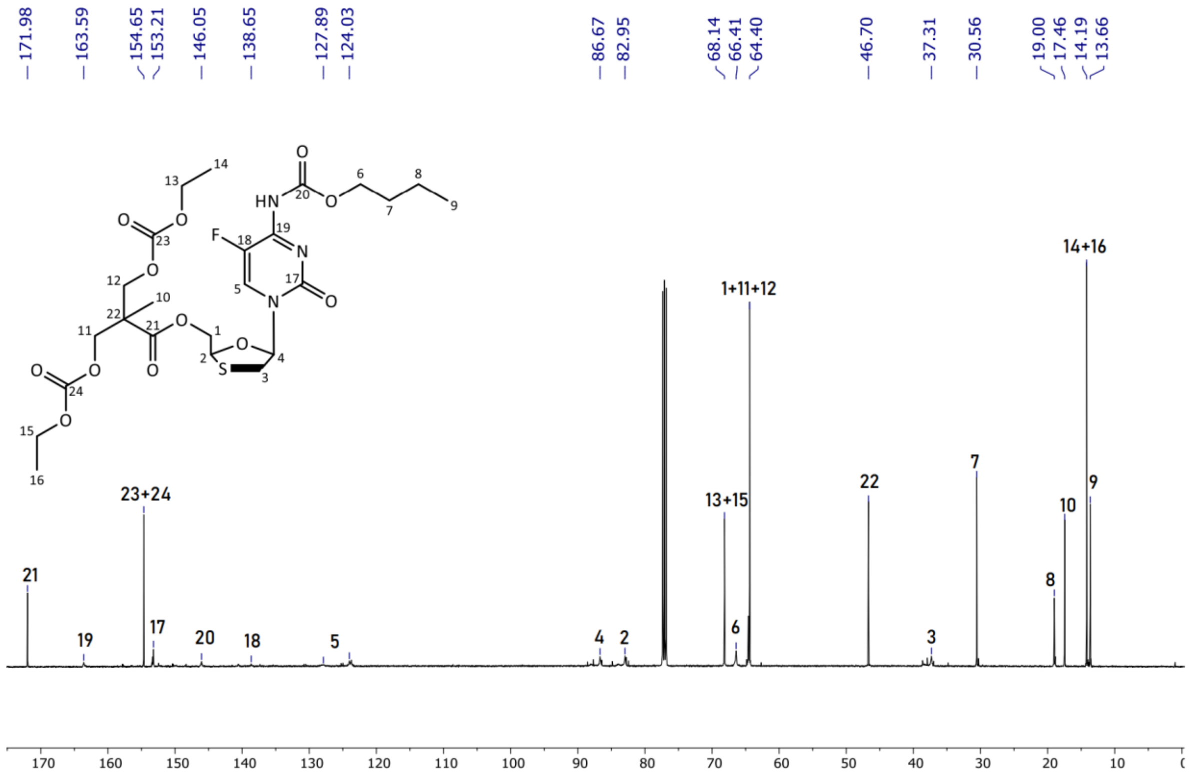

*Figure S38: ¹³C NMR ($CDCl_3$, 100 MHz) of model dicarbonate compound **12.***

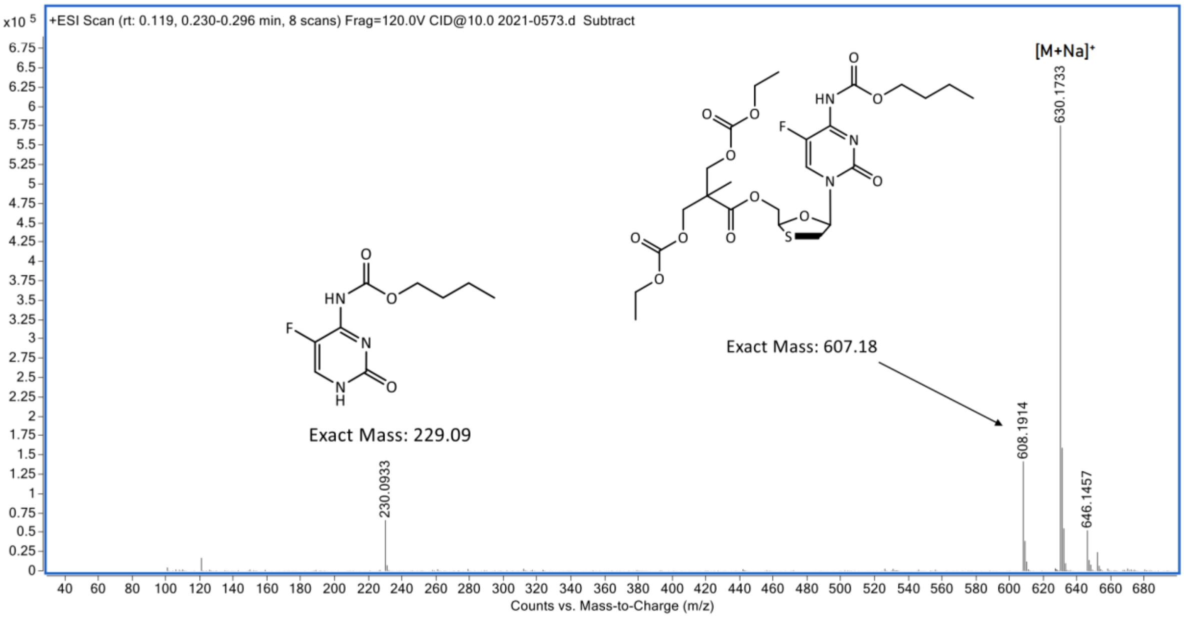

Figure S39: ESI-MS spectrum of model dicarbonate compound ***12*** *(positive ion mode).*

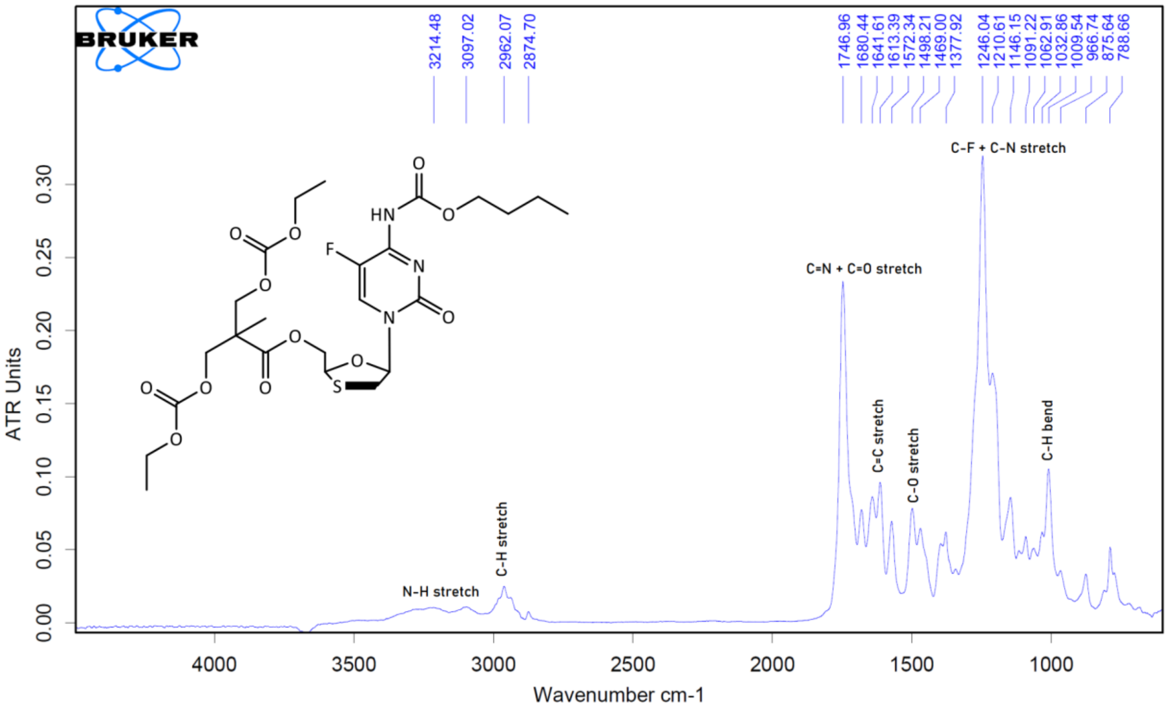

Figure S40: FTIR spectrum of model dicarbonate compound ***12.***

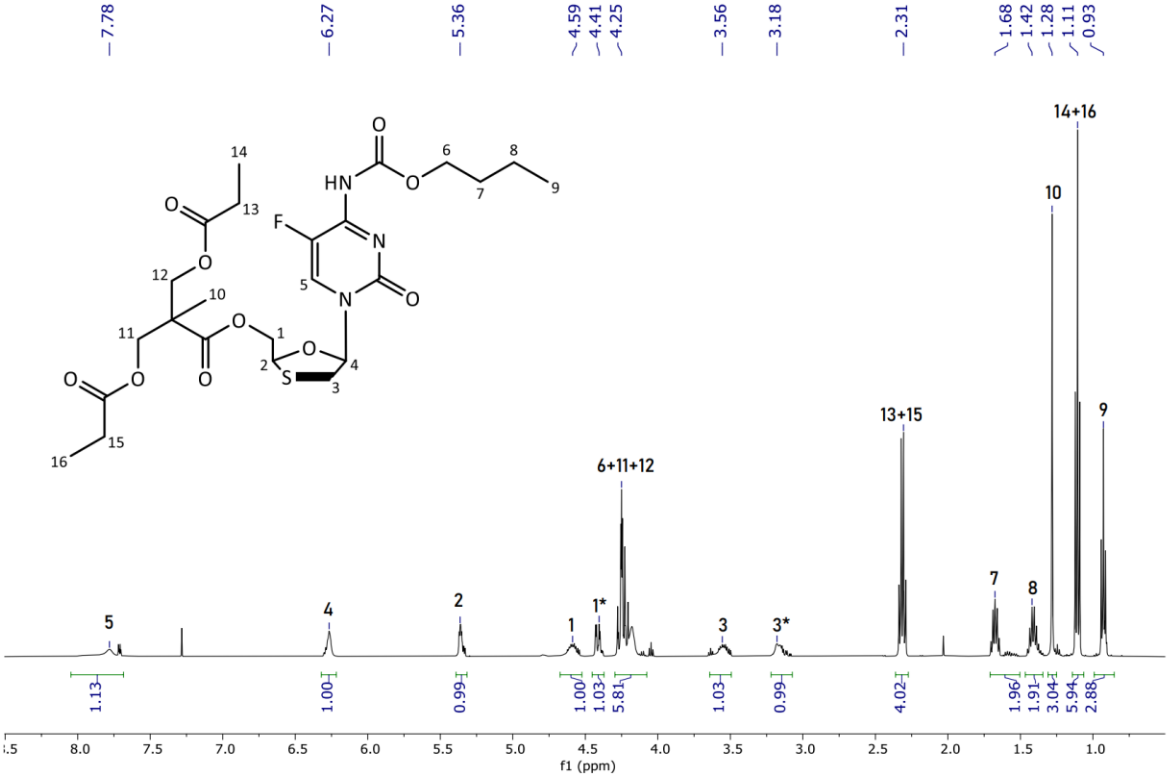

*Figure S41: ^{1}H NMR ($CDCl_3$, 500 MHz) of model diester compound **13**.*

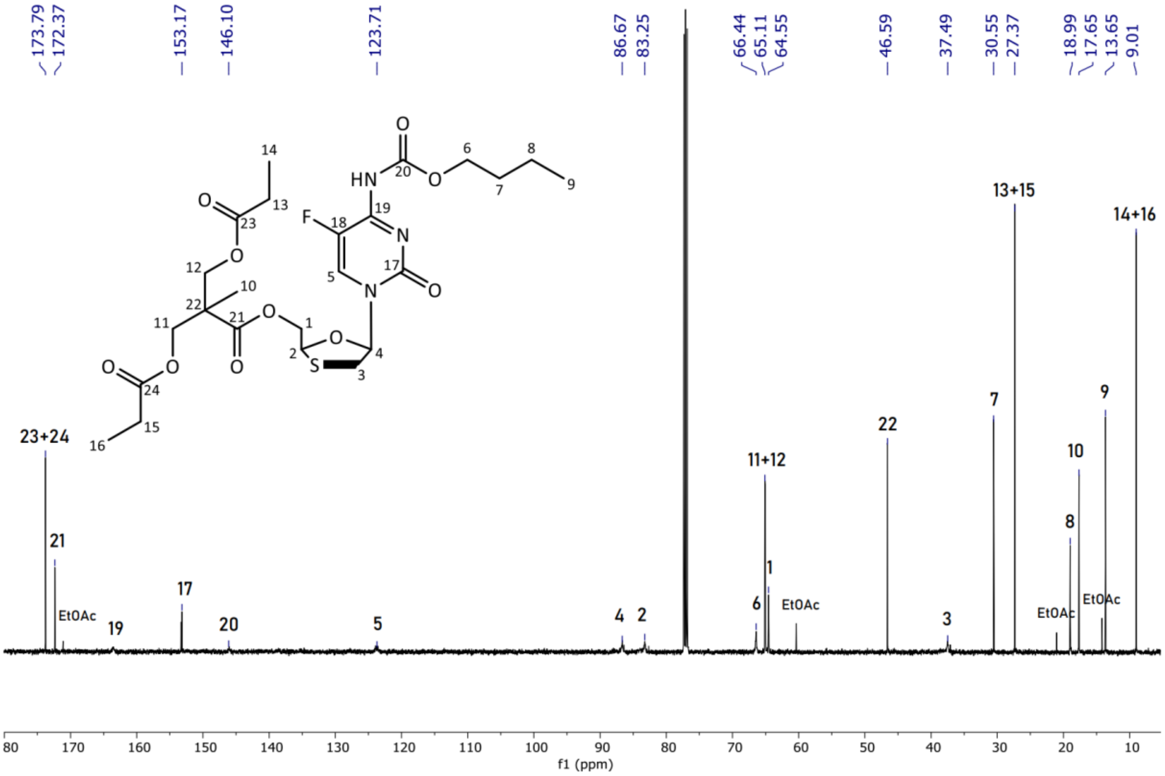

*Figure S42: ^{13}C NMR ($CDCl_3$, 100 MHz) of model diester compound **13**.*

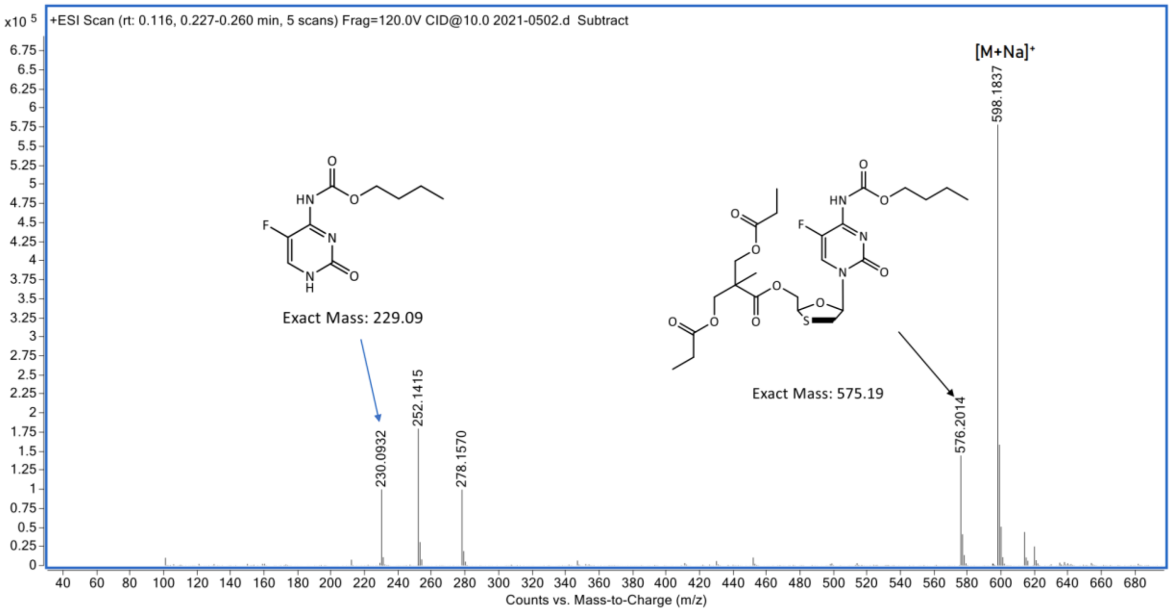

Figure S43: ESI-MS spectrum of model diester compound ***13****.*

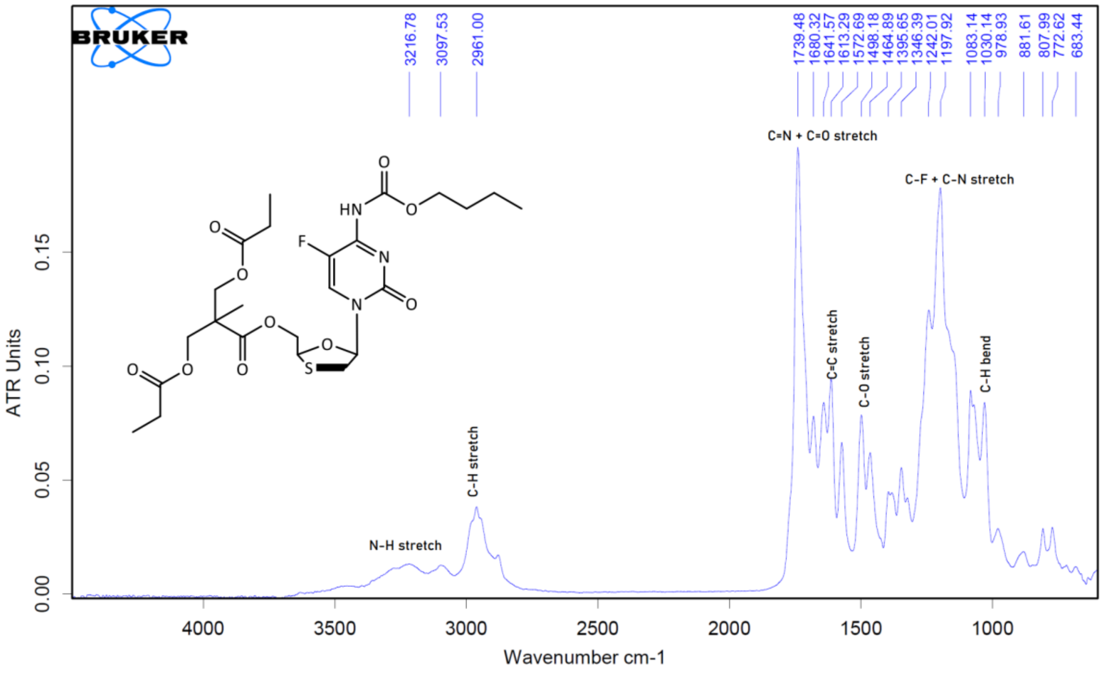

Figure S44: FTIR spectrum of model diester compound ***13****.*

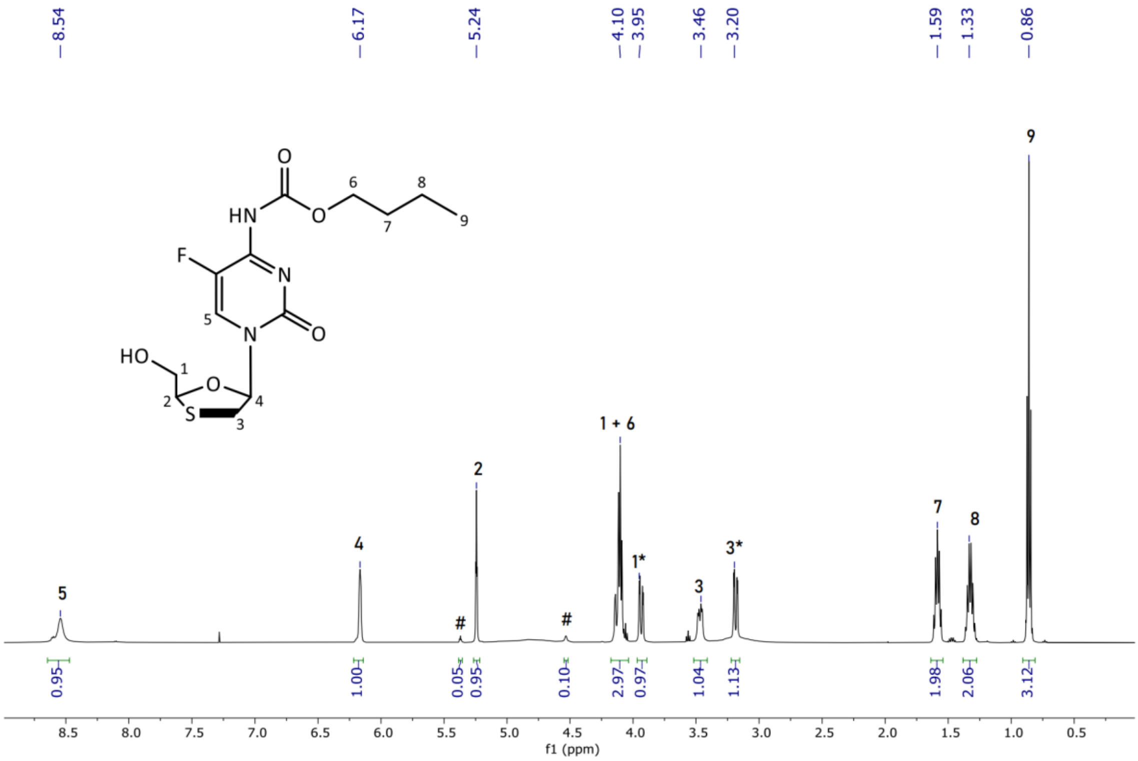

Figure S45: ^{1}H NMR ($CDCl_3$, 500 MHz) of model mono-carbamate compound **14**.

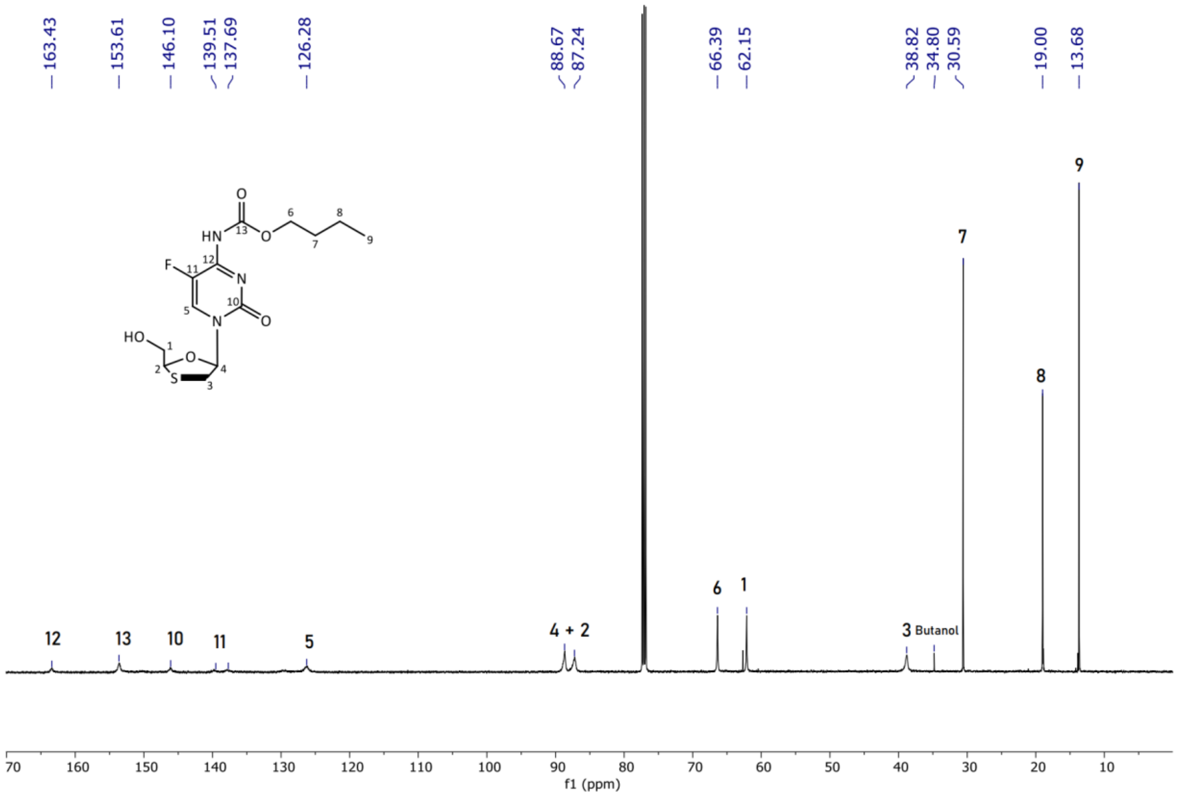

Figure S46: ^{13}C NMR ($CDCl_3$, 100 MHz) of model mono-carbamate compound **14**.

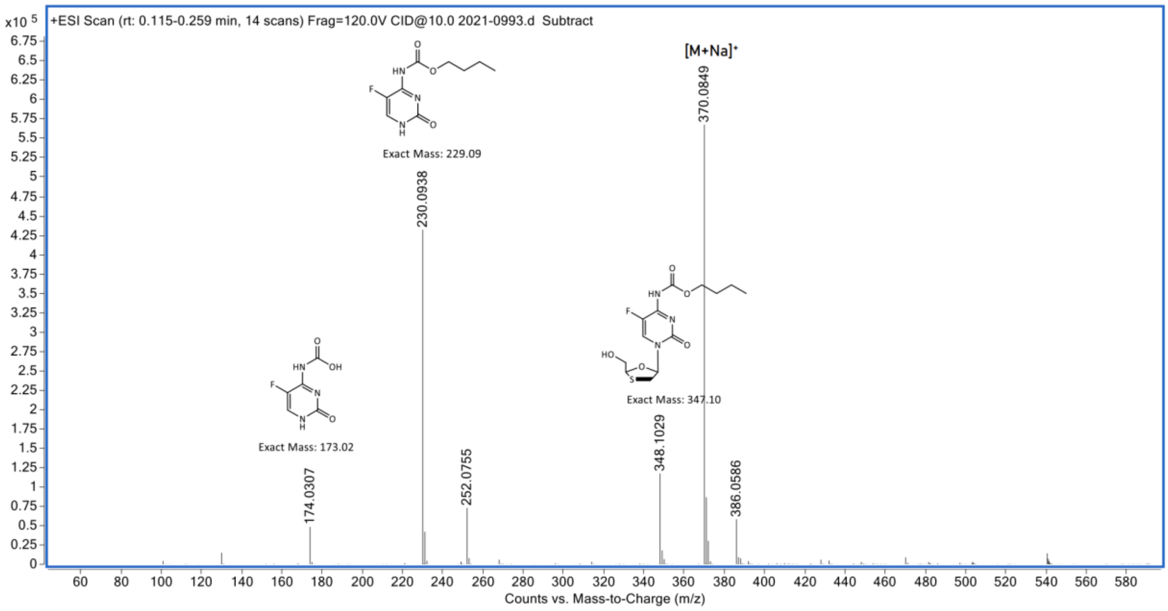

Figure S47: ESI-MS spectrum of model mono-carbamate compound ***14*** *(positive ion mode).*

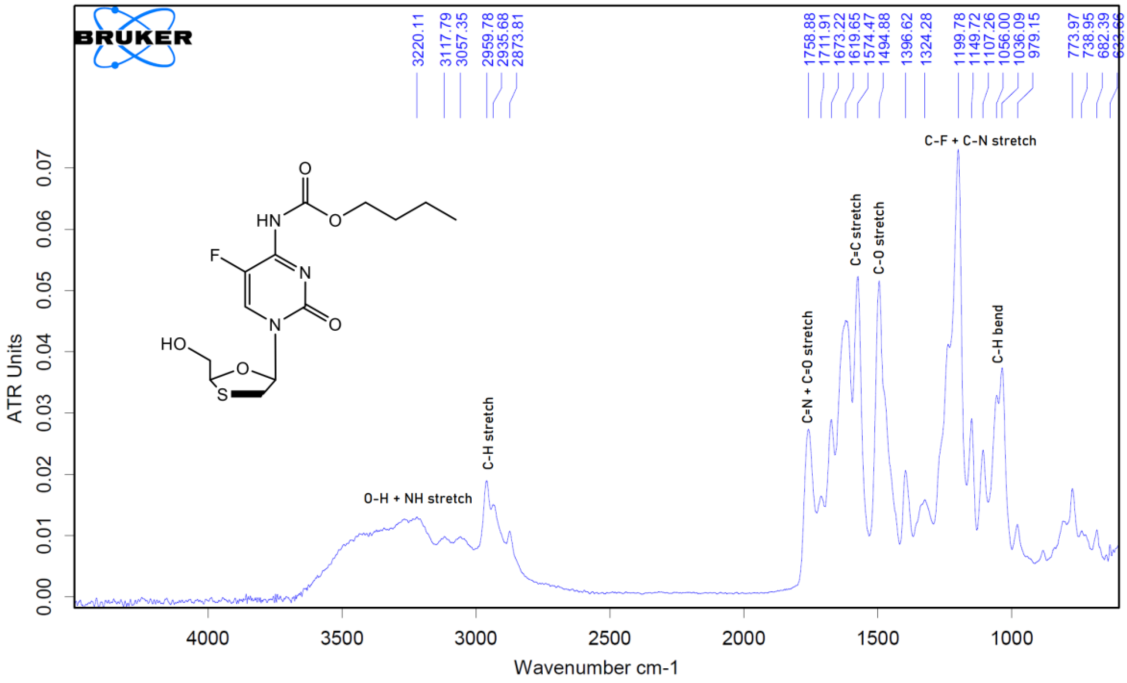

Figure S48: FTIR spectrum of model mono-carbamate compound ***14****.*

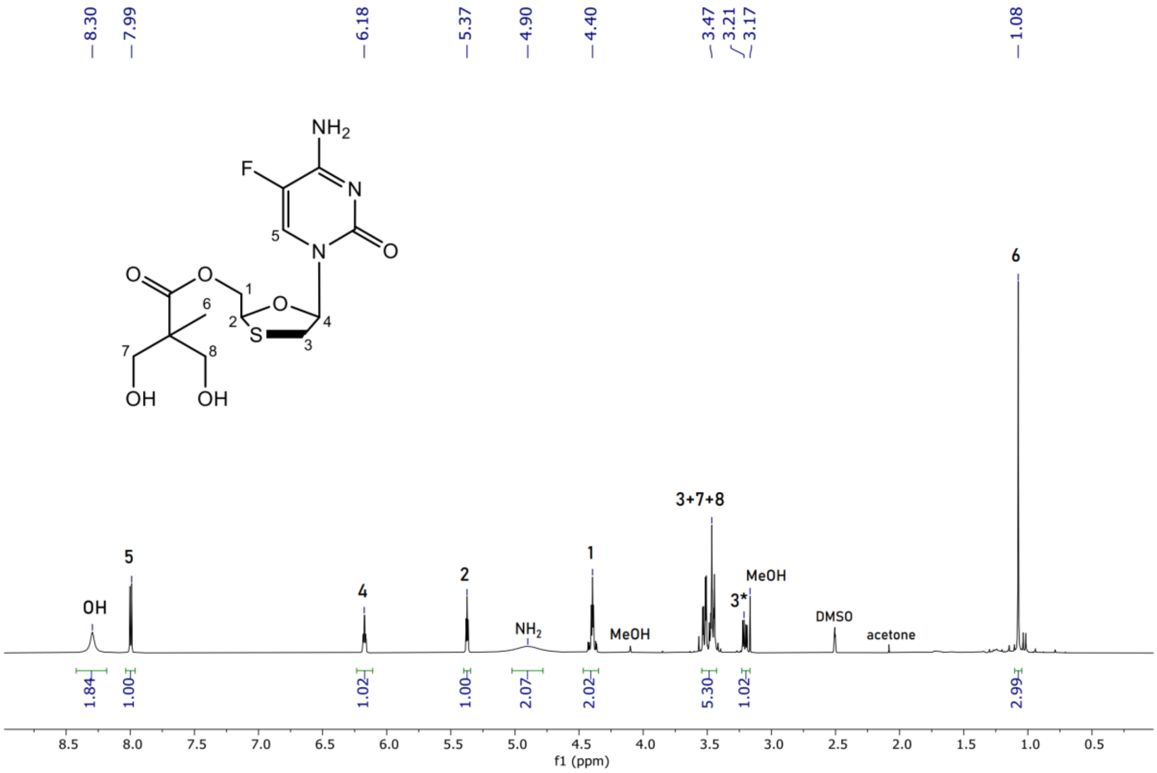

*Figure S49: ^{1}H NMR ($CDCl_3$, 500 MHz) of model monoester compound **15**.*

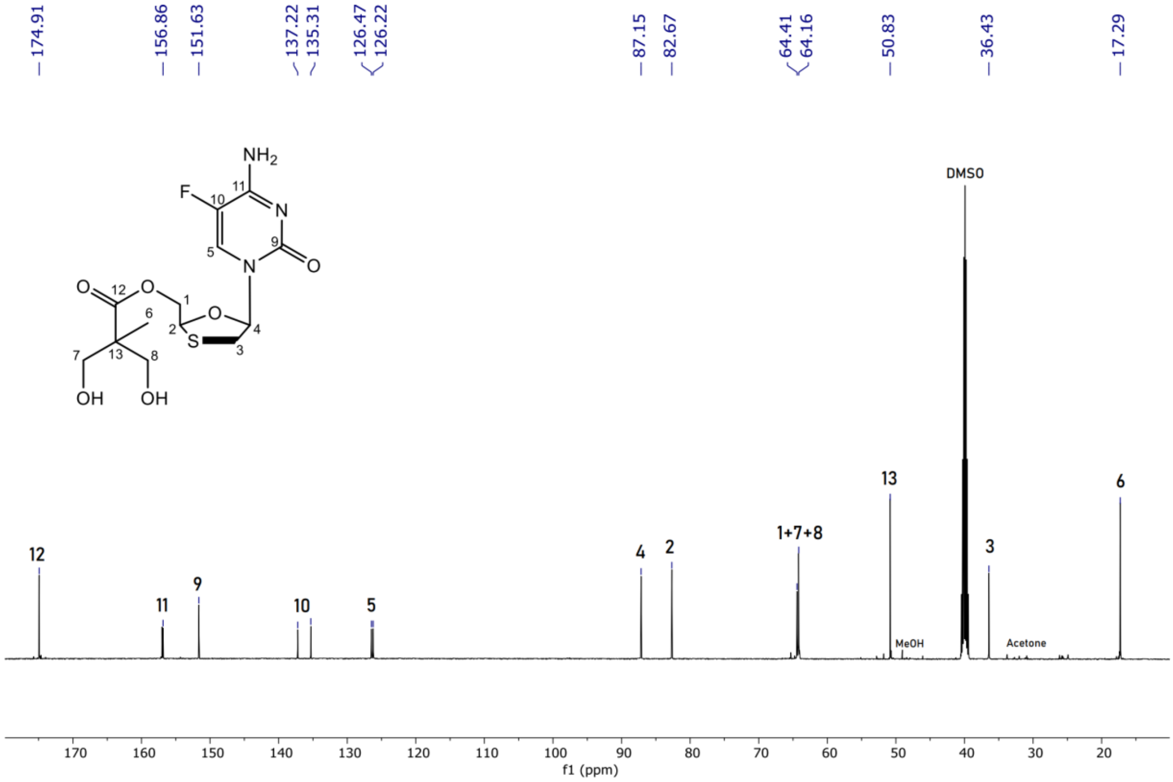

*Figure S50: ^{13}C NMR ($CDCl_3$, 100 MHz) of model monoester compound **15**.*

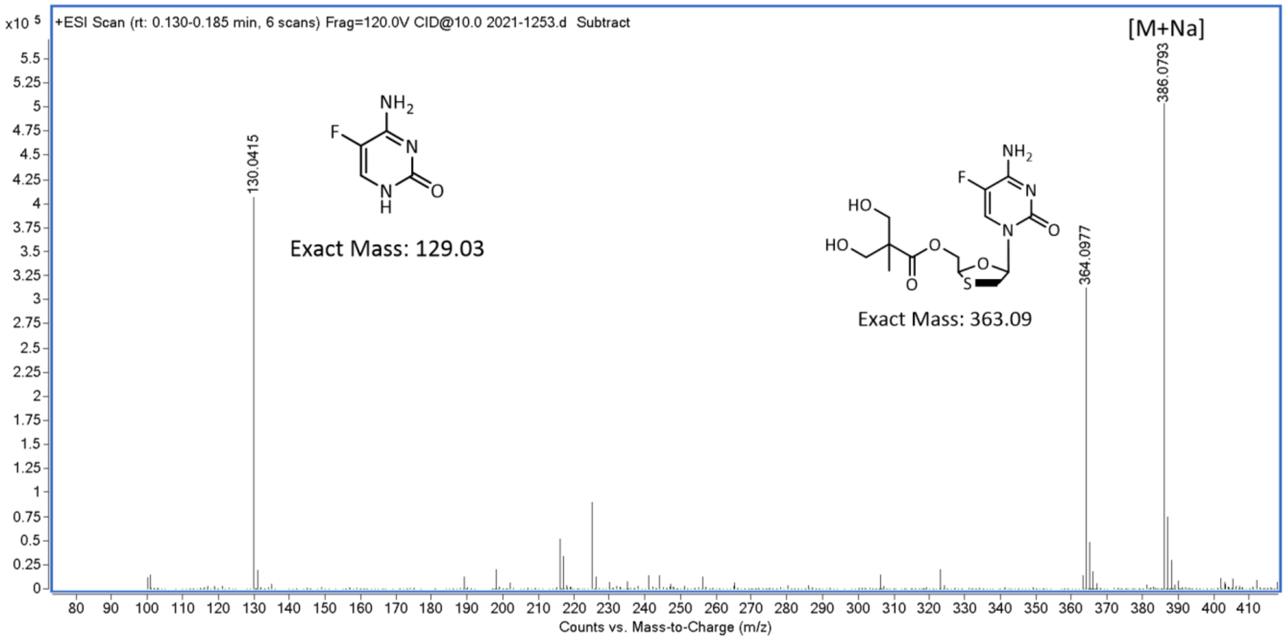

*Figure S51: ESI-MS spectrum of model monoester compound **15**.*

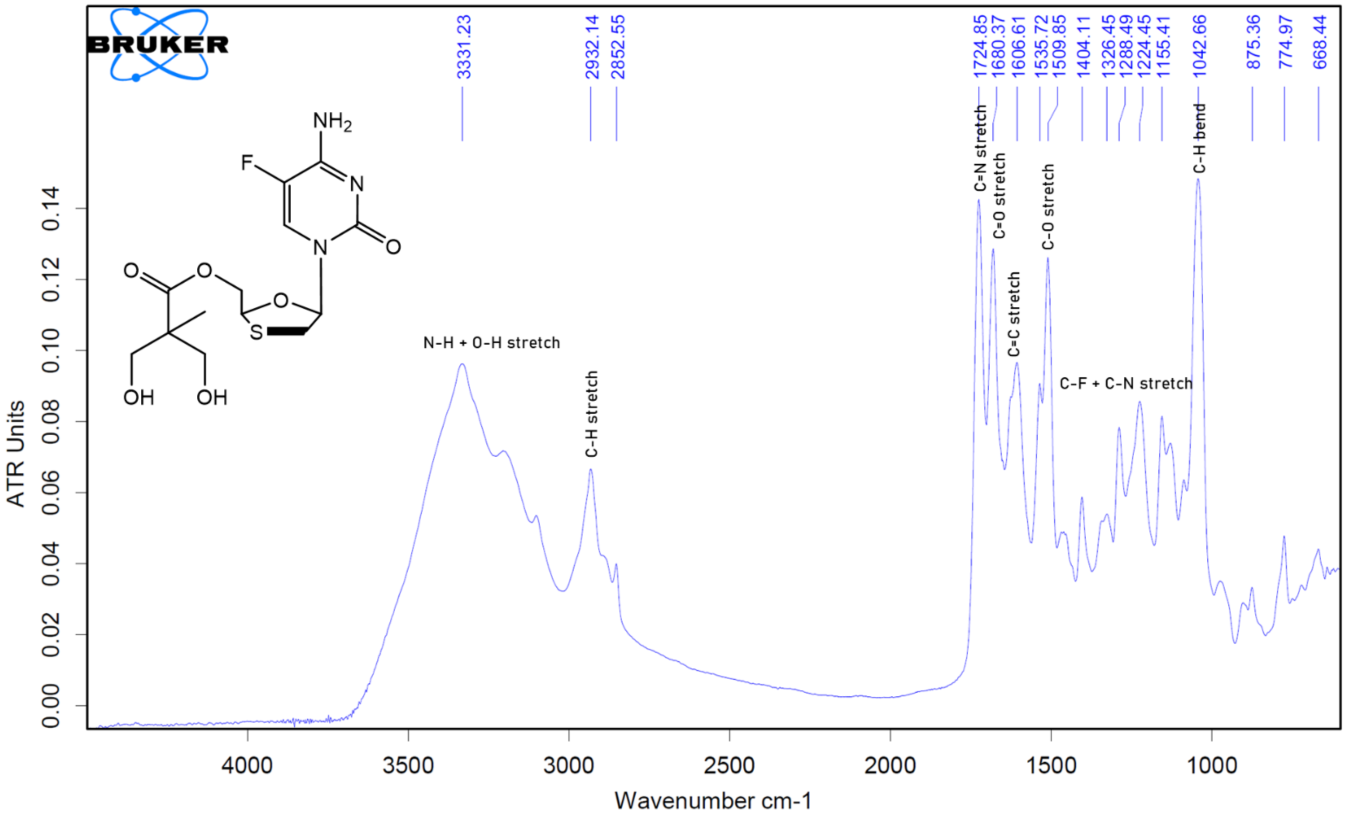

*Figure S52: FTIR spectrum of model monoester compound **15**.*

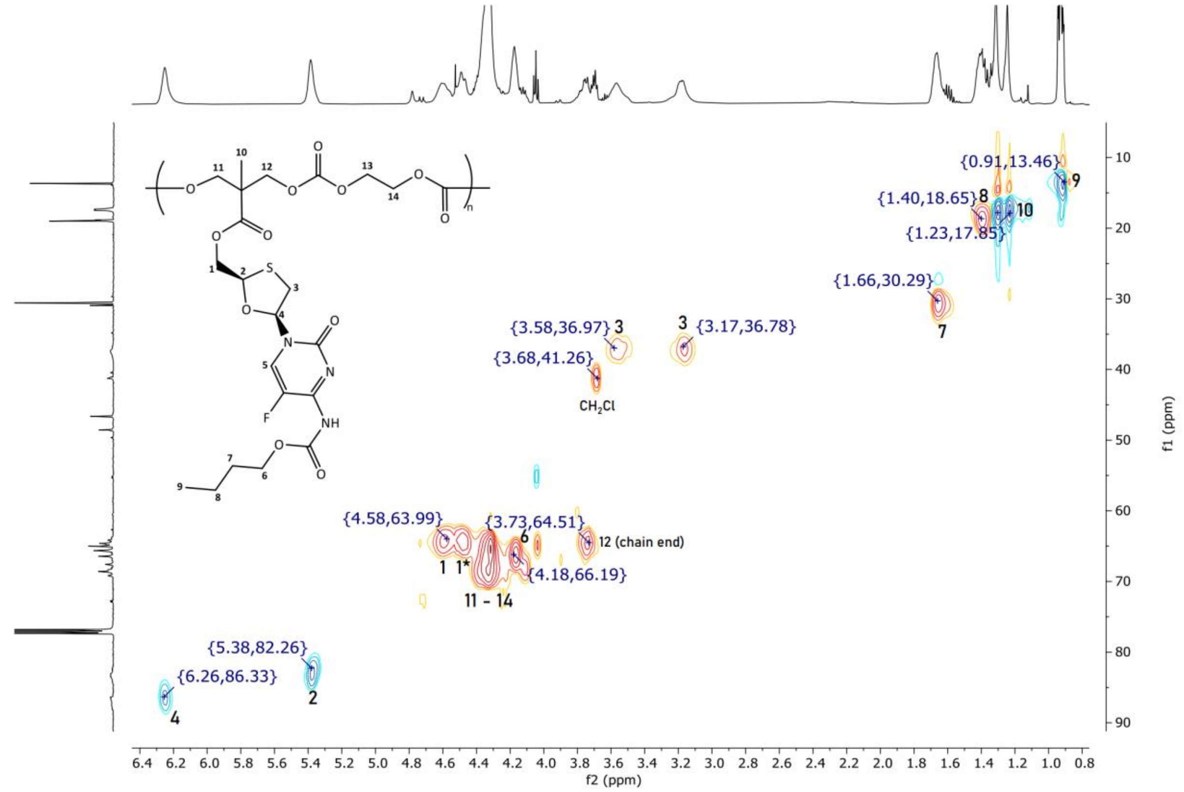

Figure S53: 2D-Heteronucelar single quantum coherence (HSQC) spectrum of polycarb(Et-nBu) ***8a****.*

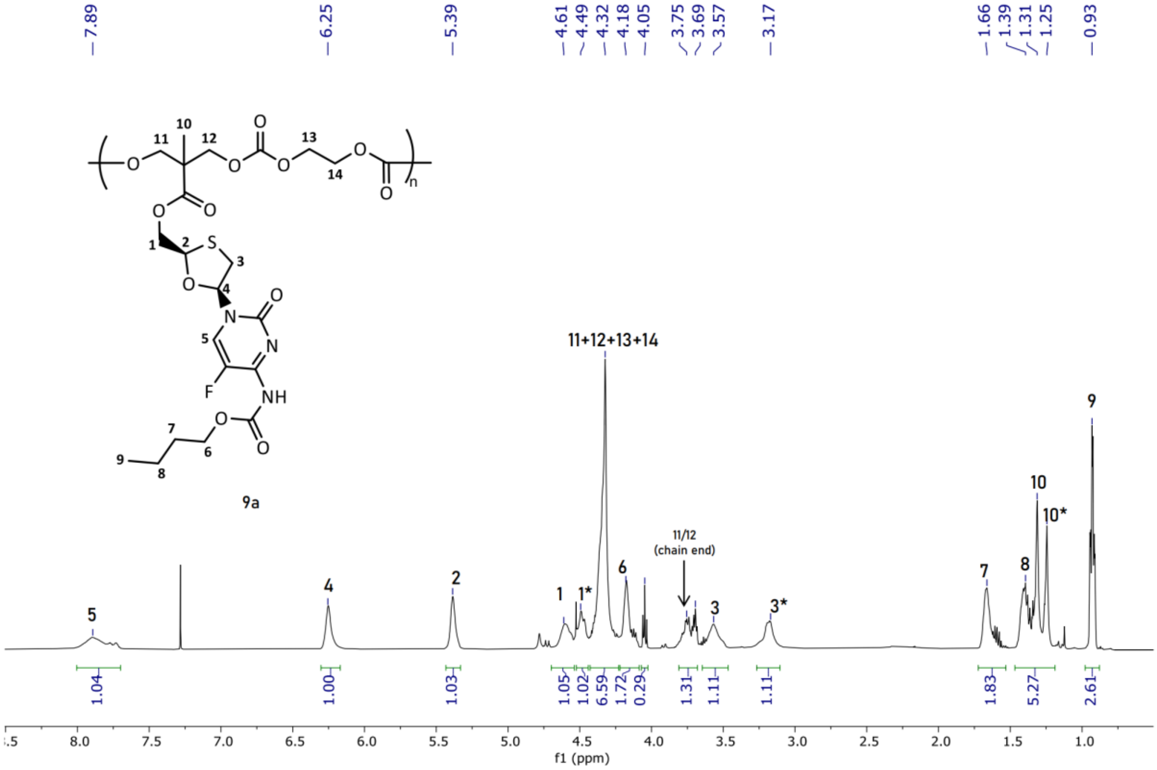

Figure S54: ^{1}H NMR ($CDCl_3$, 500 MHz) of polycarb(Et-nBu) ***8a****.*

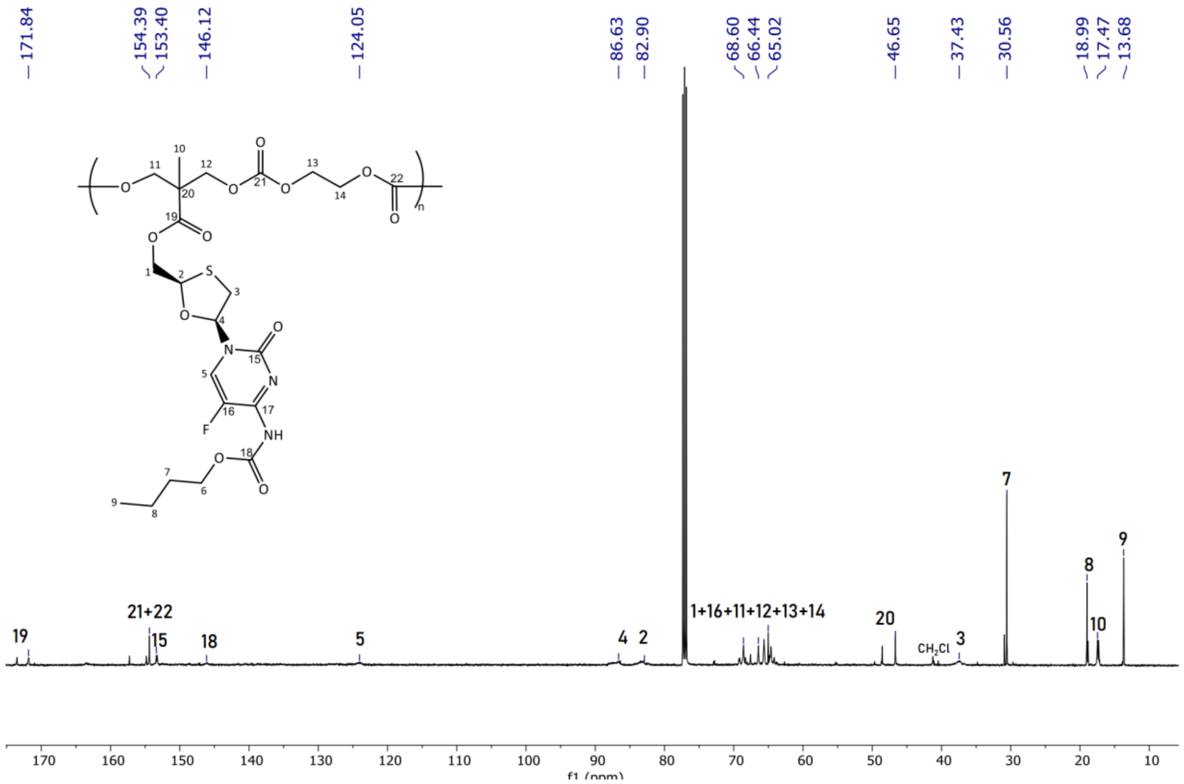

Figure S55: ^{13}C NMR ($CDCl_3$, 100 MHz) of polycarb(Et-nBu) ***8a.***

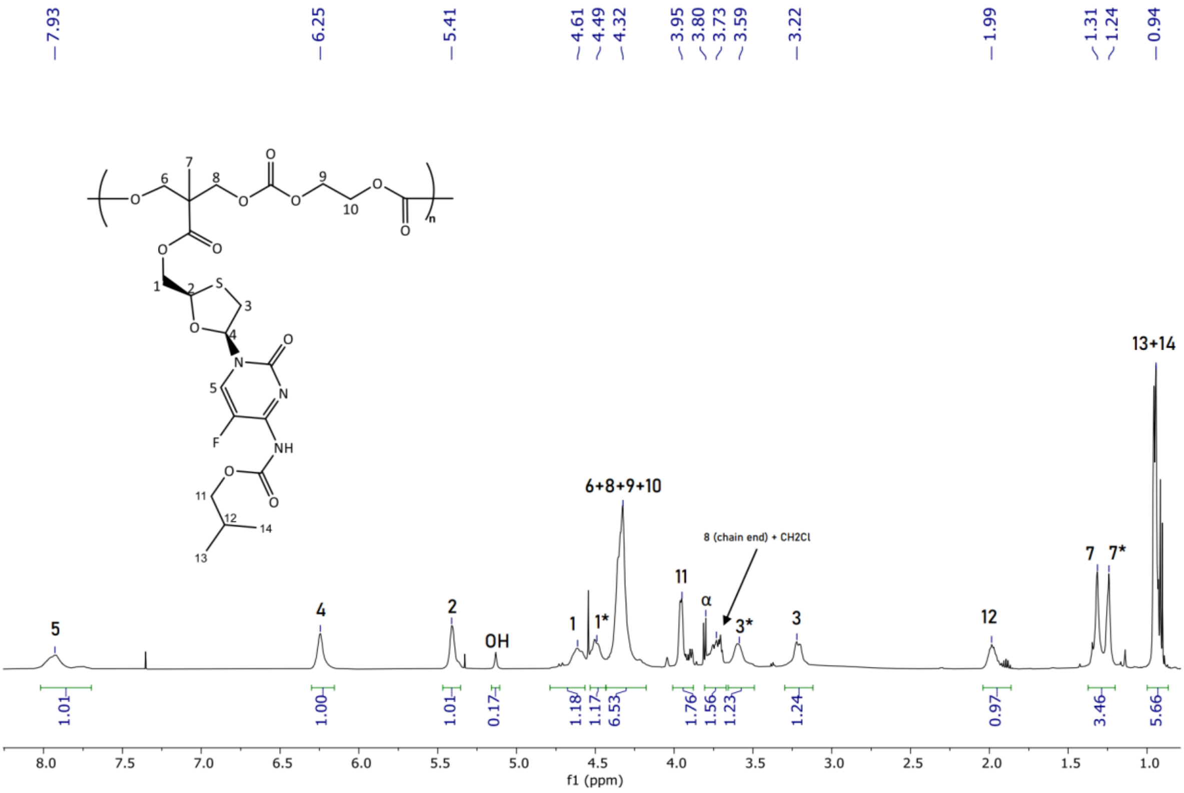

Figure S56: ^{1}H NMR ($CDCl_3$, 500 MHz) of polycarb(Et-iBu) ***8b****.*

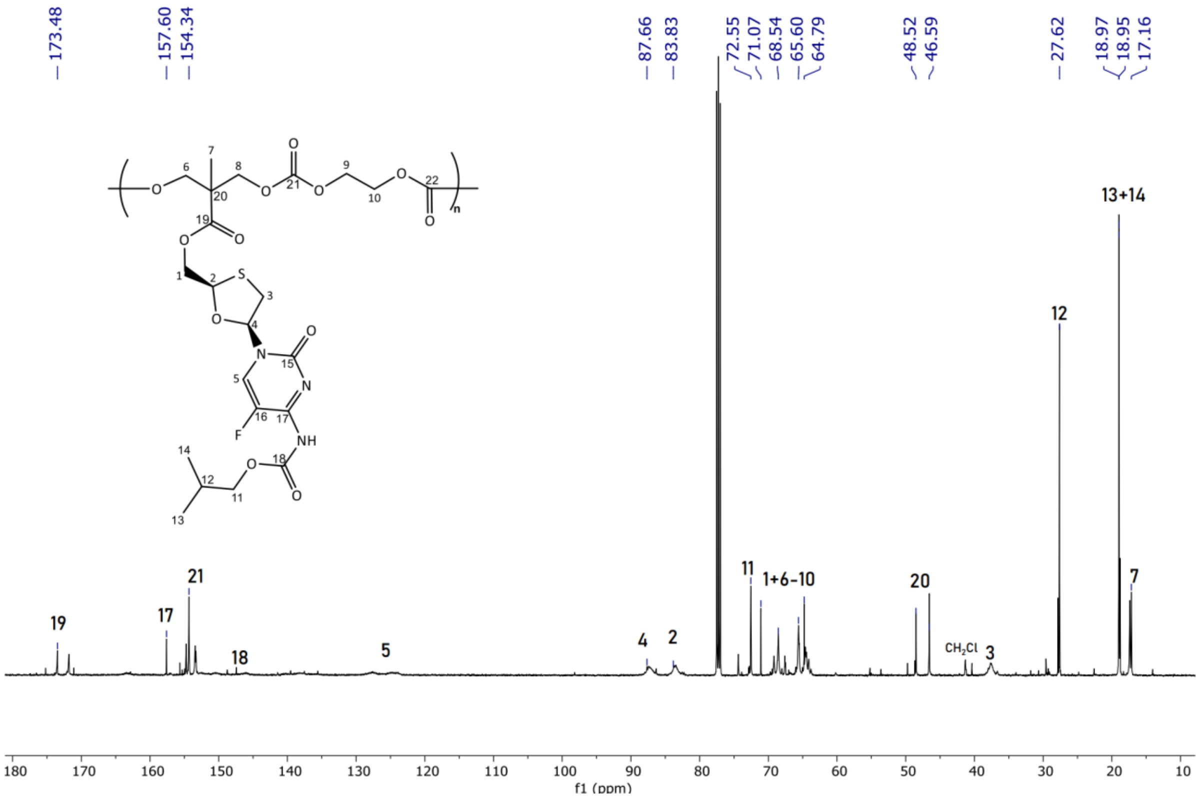

Figure S57: ^{13}C NMR ($CDCl_3$, 100 MHz) polycarb(Et-iBu) ***8b.***

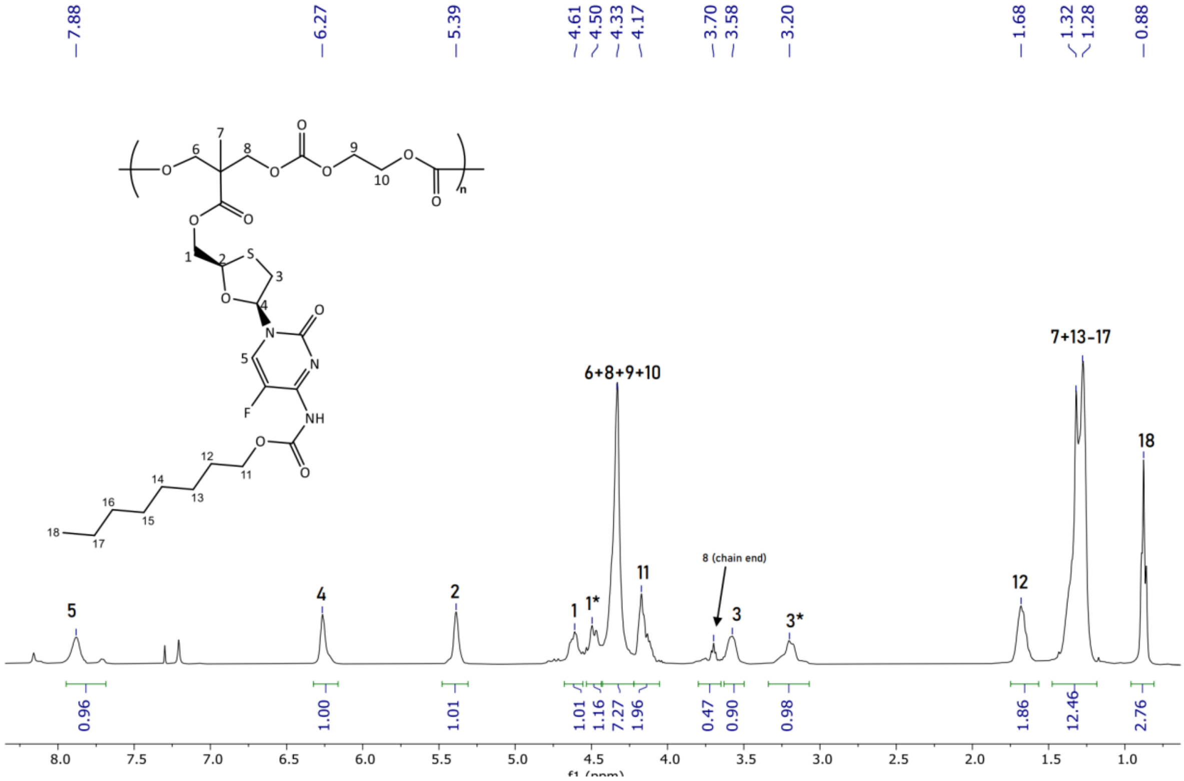

Figure S58: ^{1}H NMR ($CDCl_3$, 500 MHz) of polycarb(Et-nOct) ***8c****.*

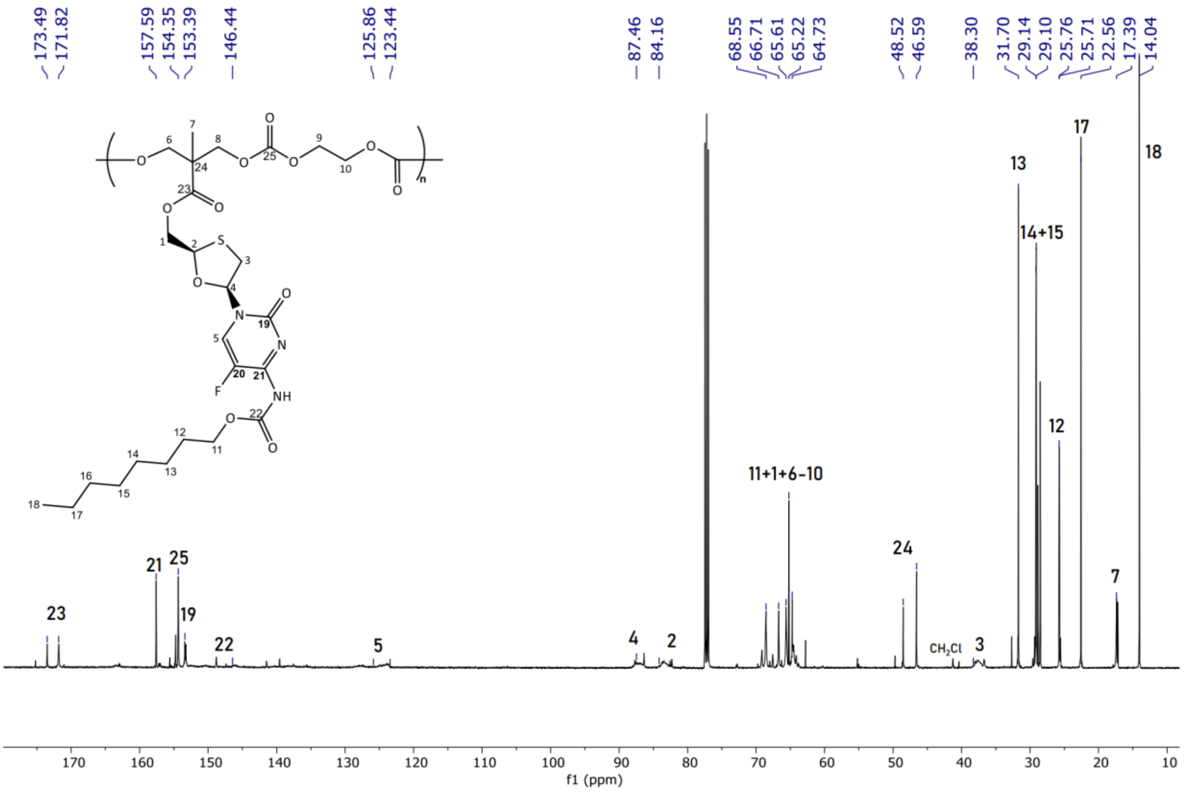

Figure S59: ^{13}C NMR ($CDCl_3$, 100 MHz) of polycarb(Et-nOct) ***8c****.*

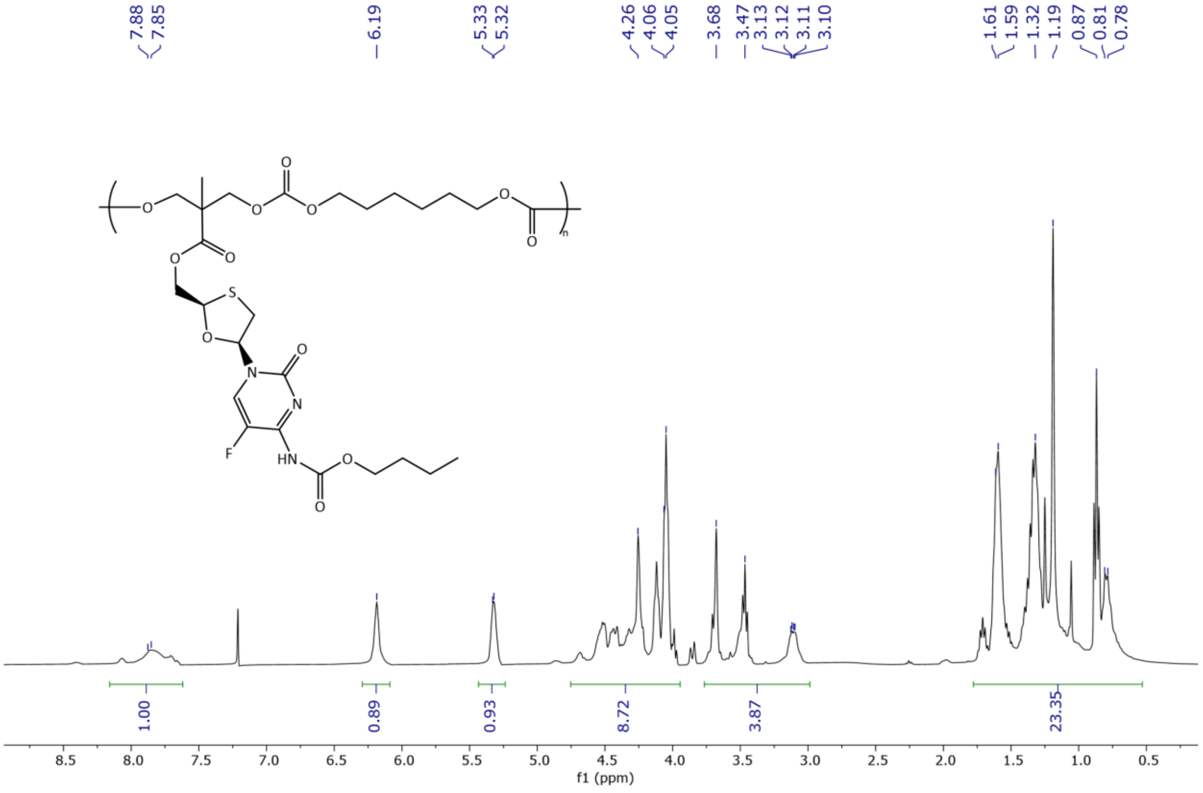

Figure S60: ^{1}H NMR ($CDCl_3$, 500 MHz) of polycarb(Hex-nBu) ***9a****.*

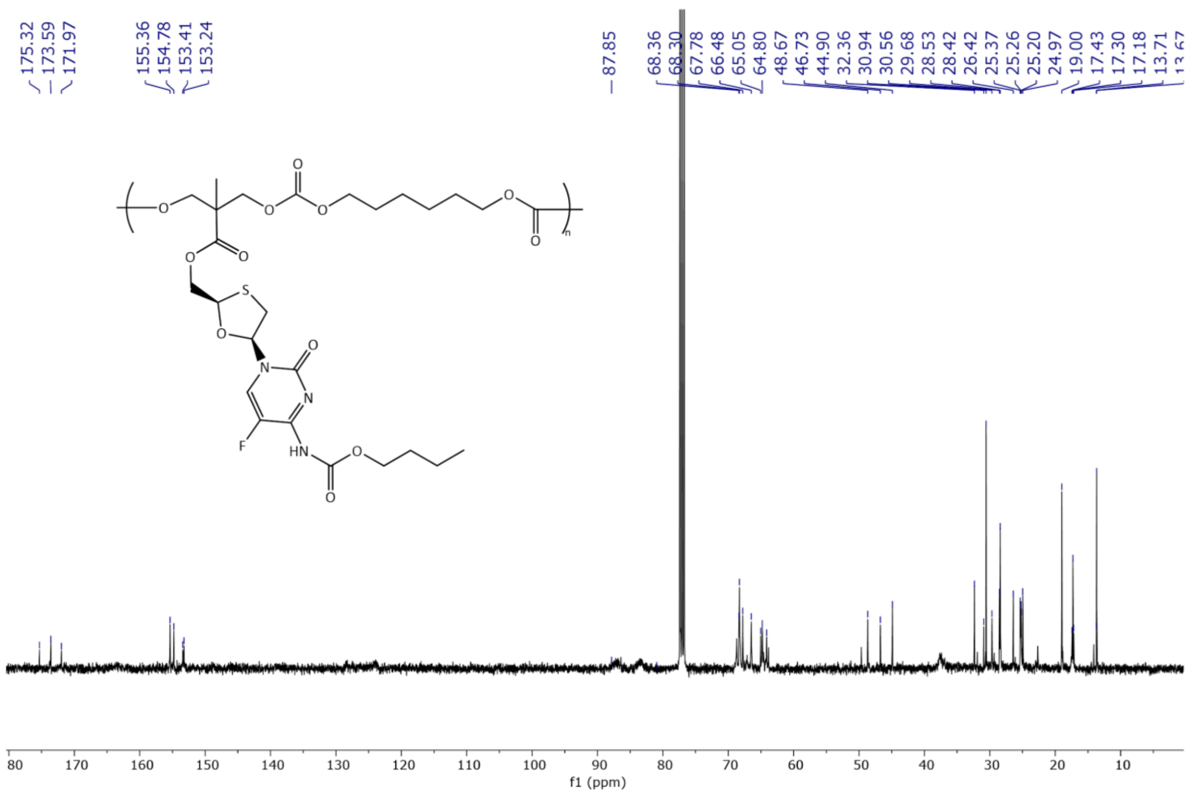

Figure S61: ^{13}C NMR ($CDCl_3$, 100 MHz) of polycarb(Hex-nBu) ***9a.***

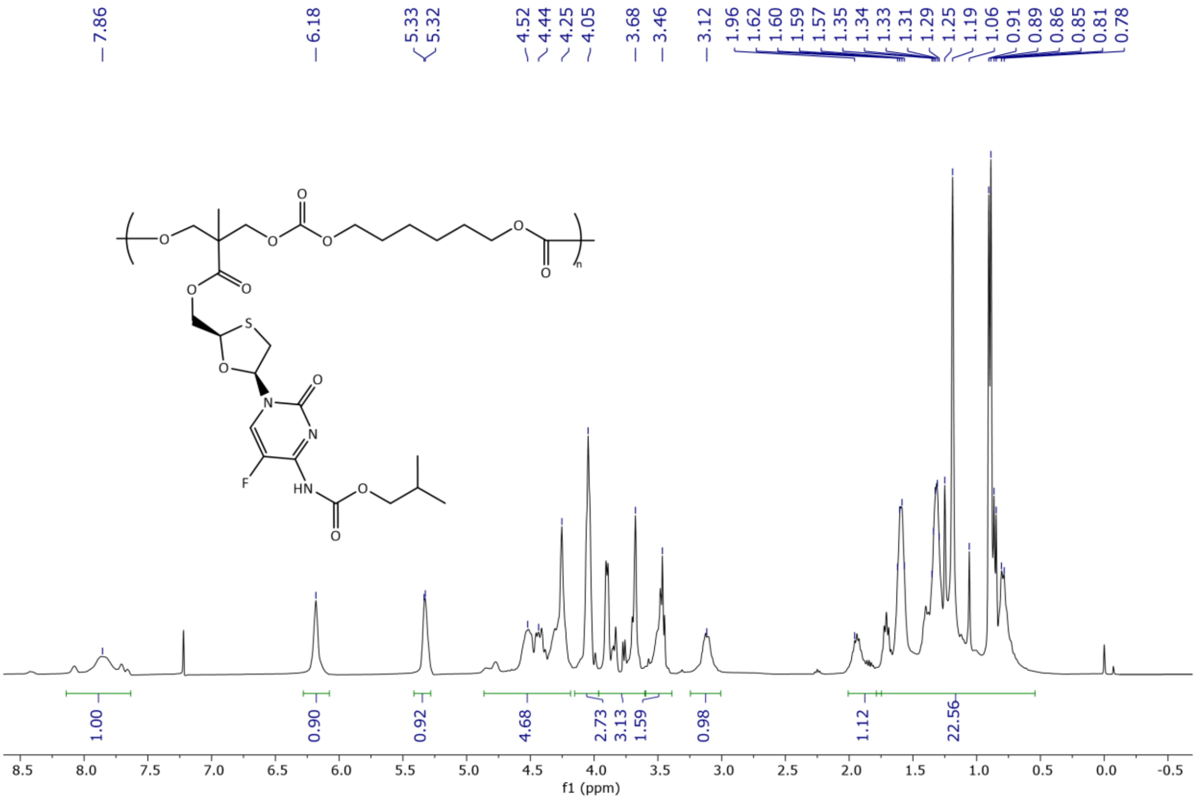

Figure S62: ^{1}H NMR ($CDCl_3$, 500 MHz) of polycarb(Hex-iBu) ***9b***.

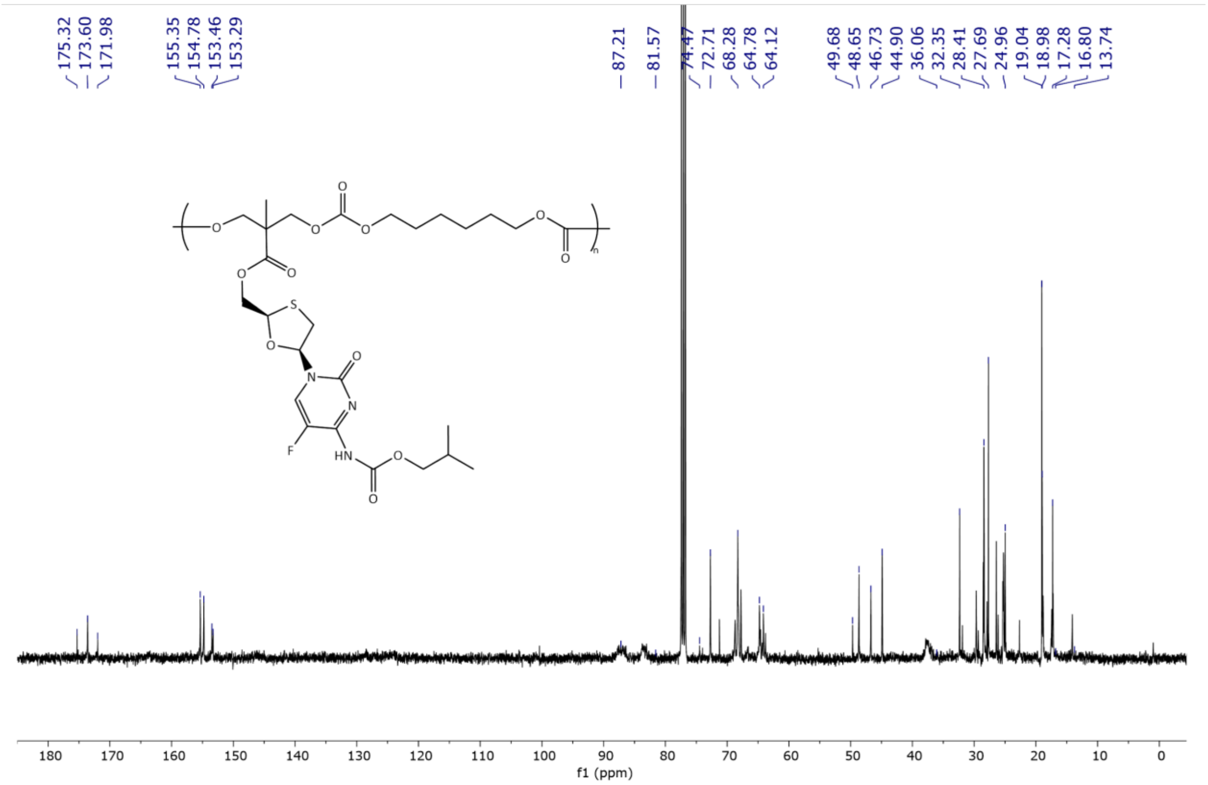

Figure S63: ^{13}C NMR (CDCl$_3$, 100 MHz) of polycarb(Hex-iBu) ***9b.***

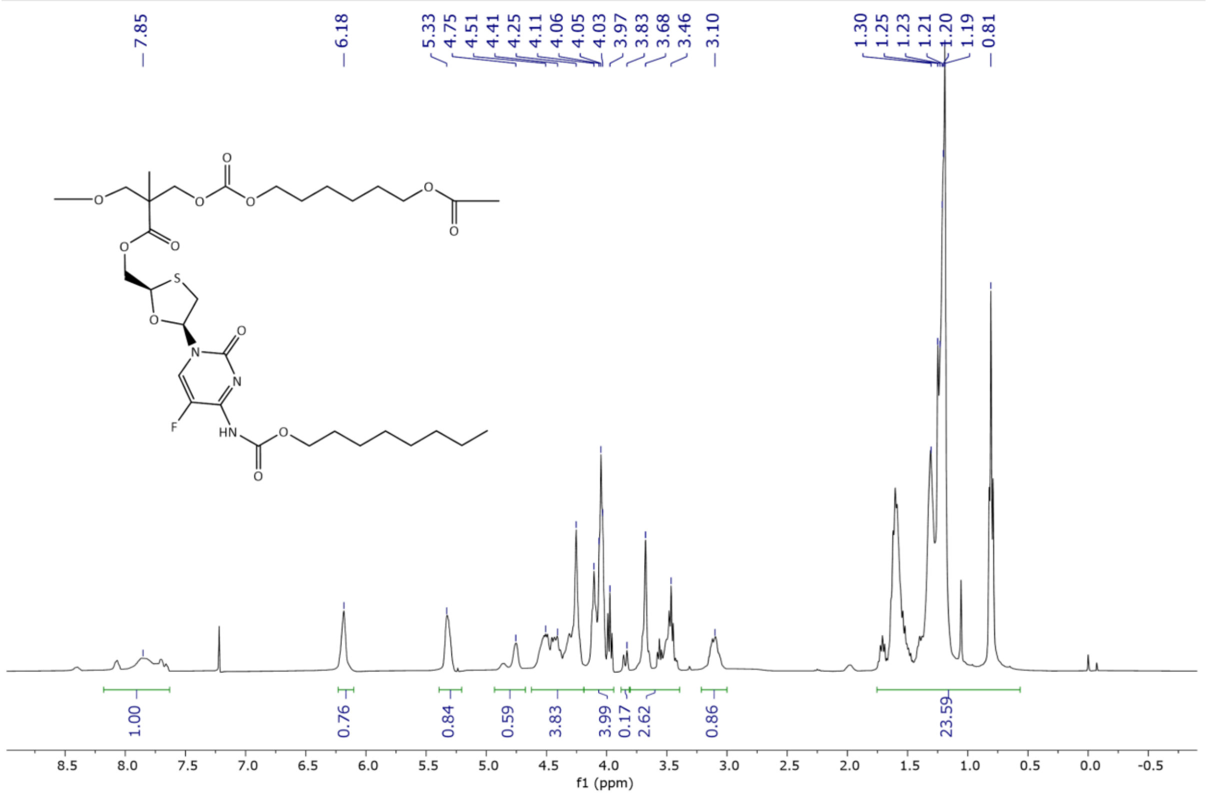

Figure S64: ^{1}H NMR (CDCl$_3$, 500 MHz) of polycarb(Hex-Oct) ***9c****.*

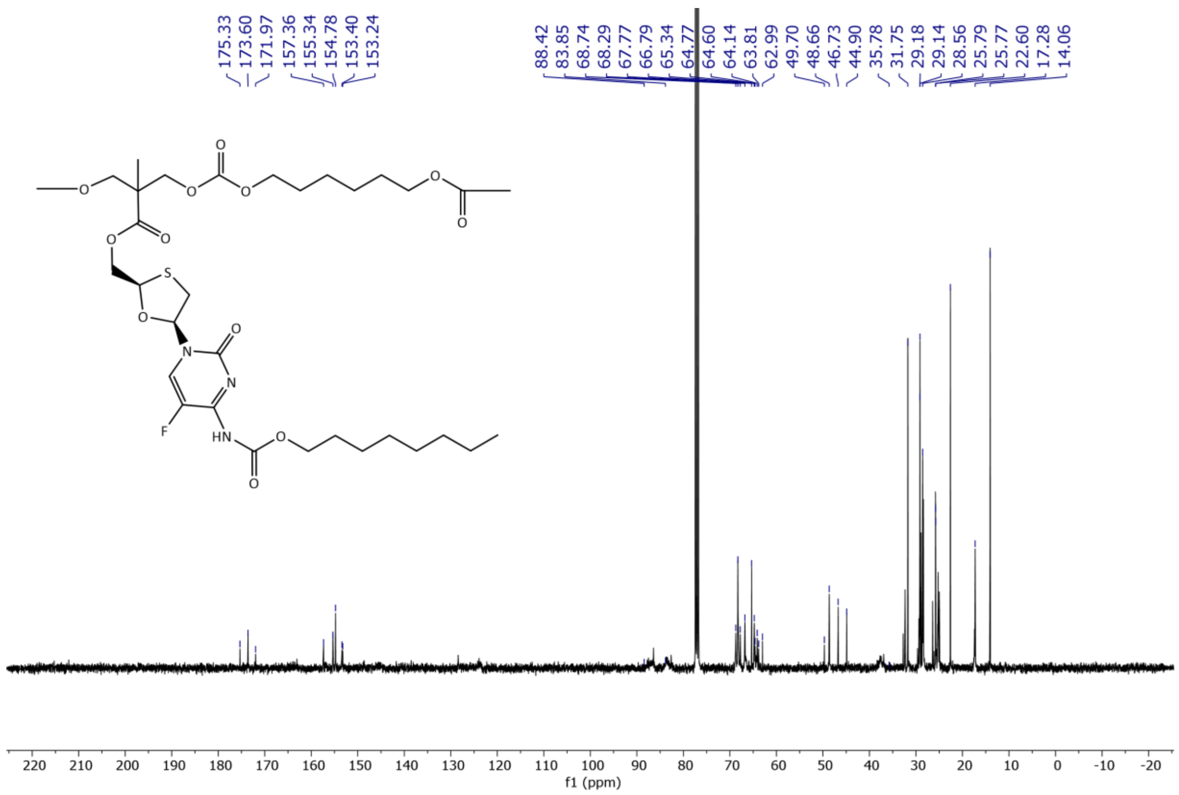

Figure S65: ^{13}C NMR ($CDCl_3$, 100 MHz) of polycarb(Hex-oct) ***9c****.*

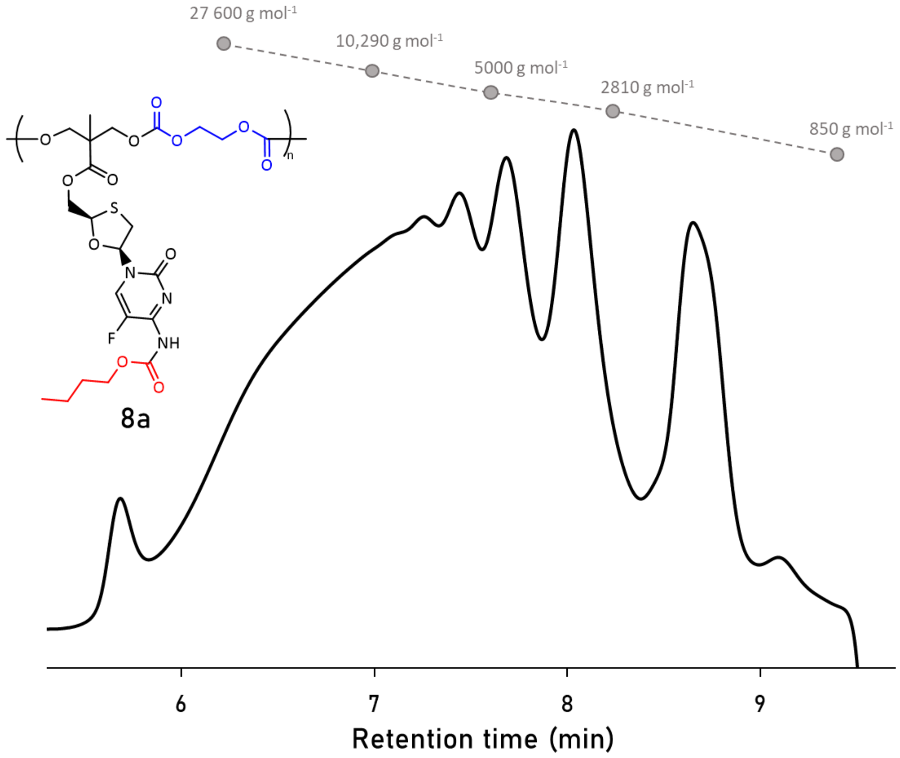

Figure S66: SEC chromatogram of polycarb(Et-nBu) ***8a****.*

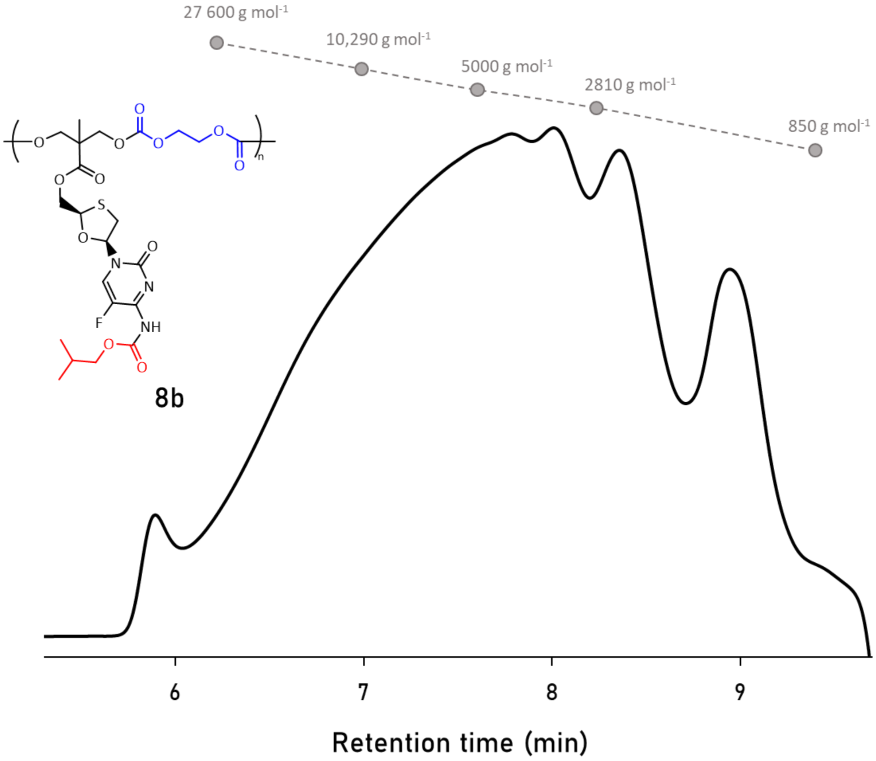

Figure S67: SC chromatogram of polycarb(Et-iBu) ***8b***.

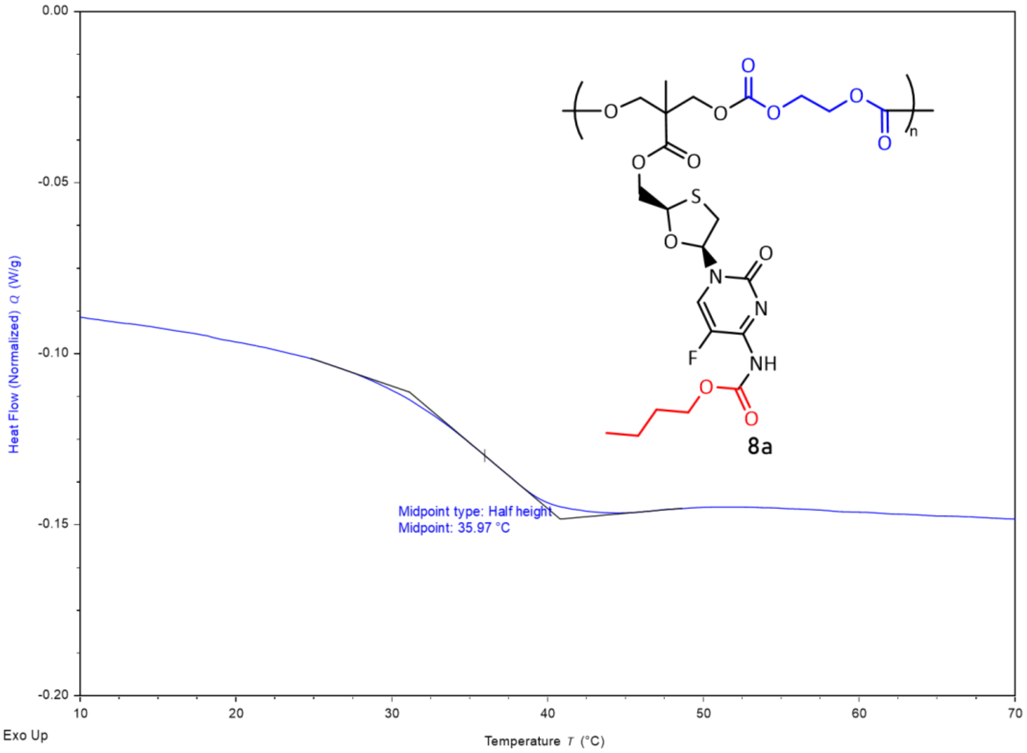

Figure S68: DSC chromatogram of polycarb(Et-nBu) ***8a***.

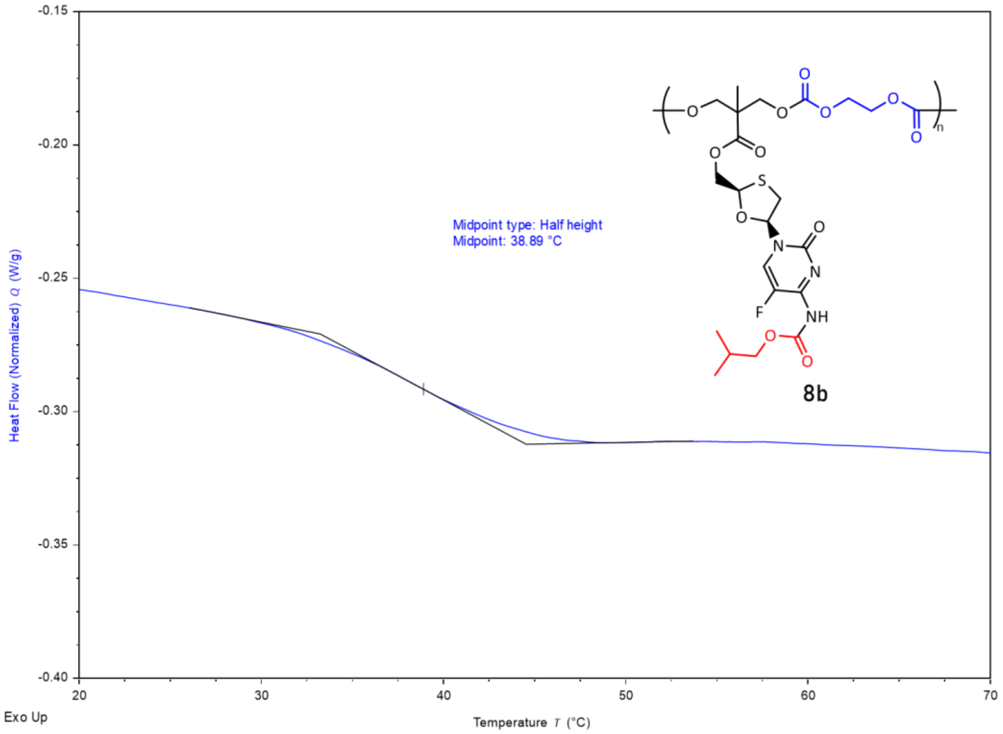

Figure S69: DSC chromatogram of polycarb(Et-iBu) ***8b***

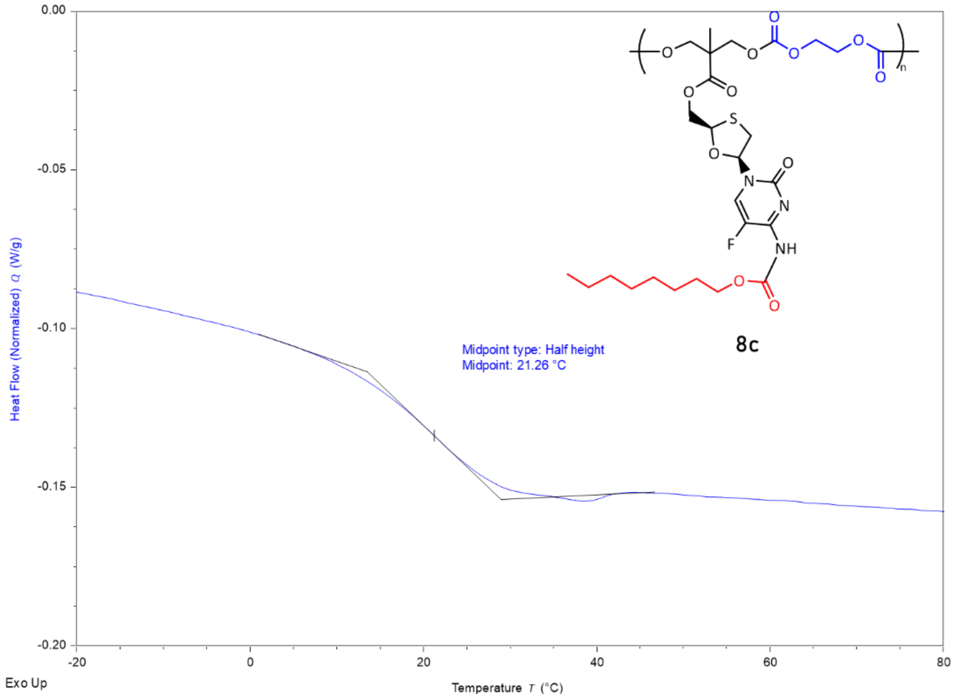

Figure S70: DSC chromatogram of polycarb(Et-Oct) ***8c.***

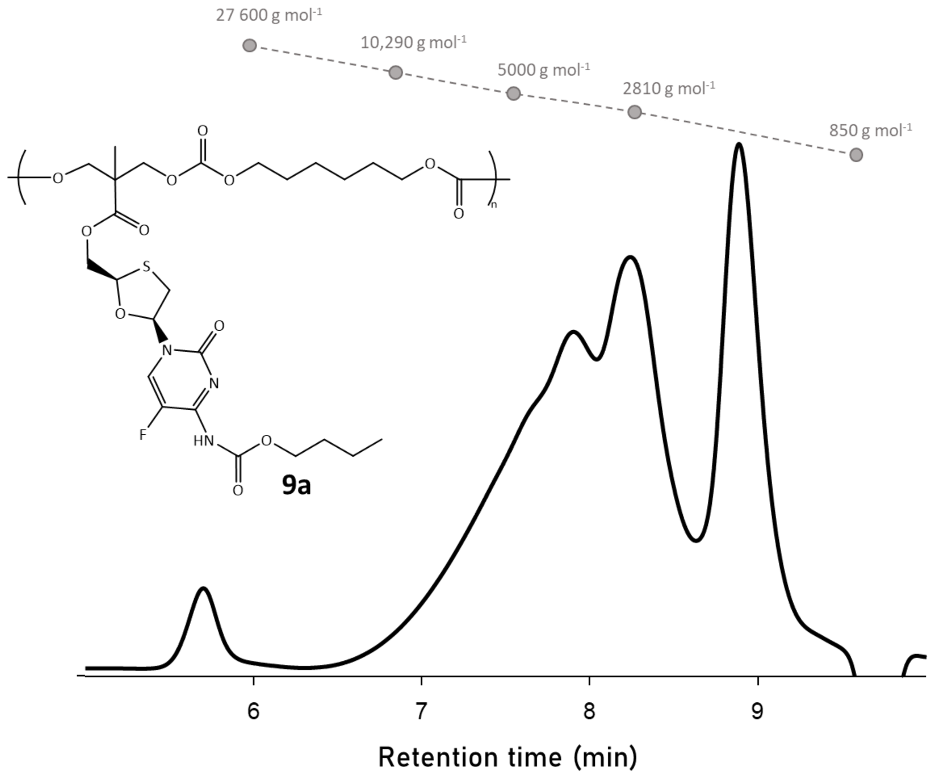

Figure S71: SEC chromatogram of polycarb(Hex-nBu) ***9a****.*

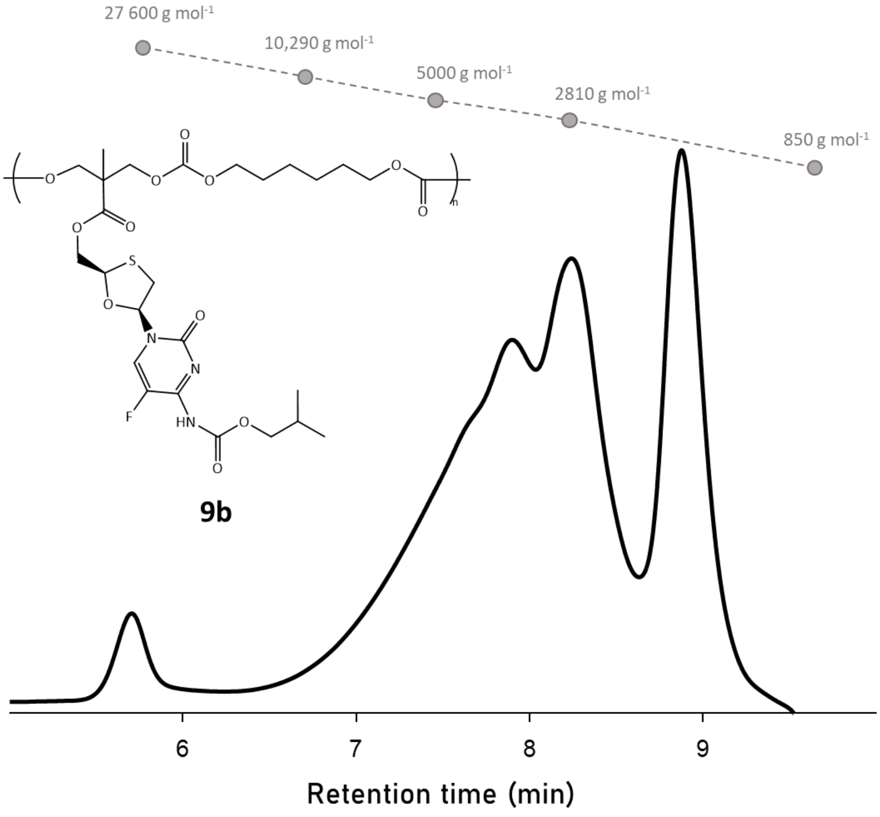

Figure S72: SEC chromatogram of polycarb(Hex-iBu) ***9b.***

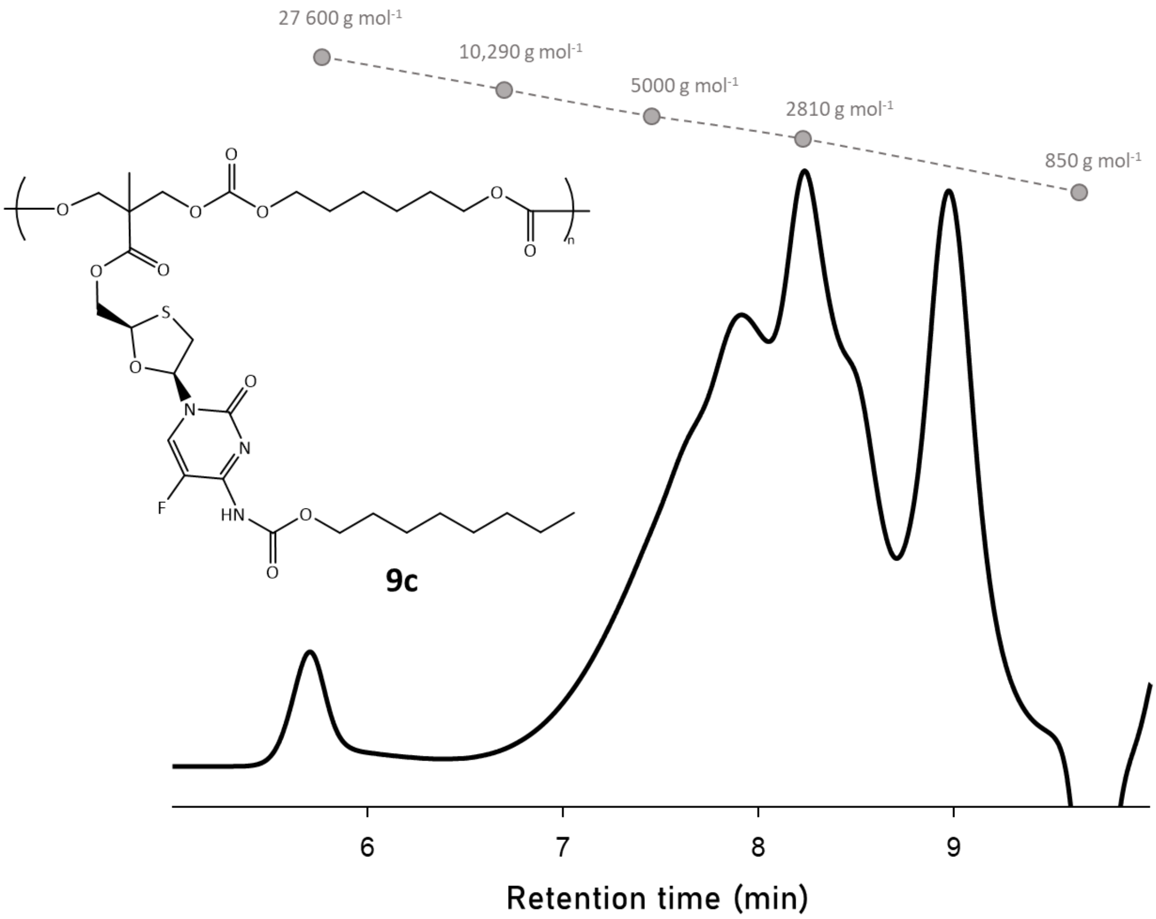

Figure S73: SEC chromatogram of polycarb(Hex-Oct) ***9c.***

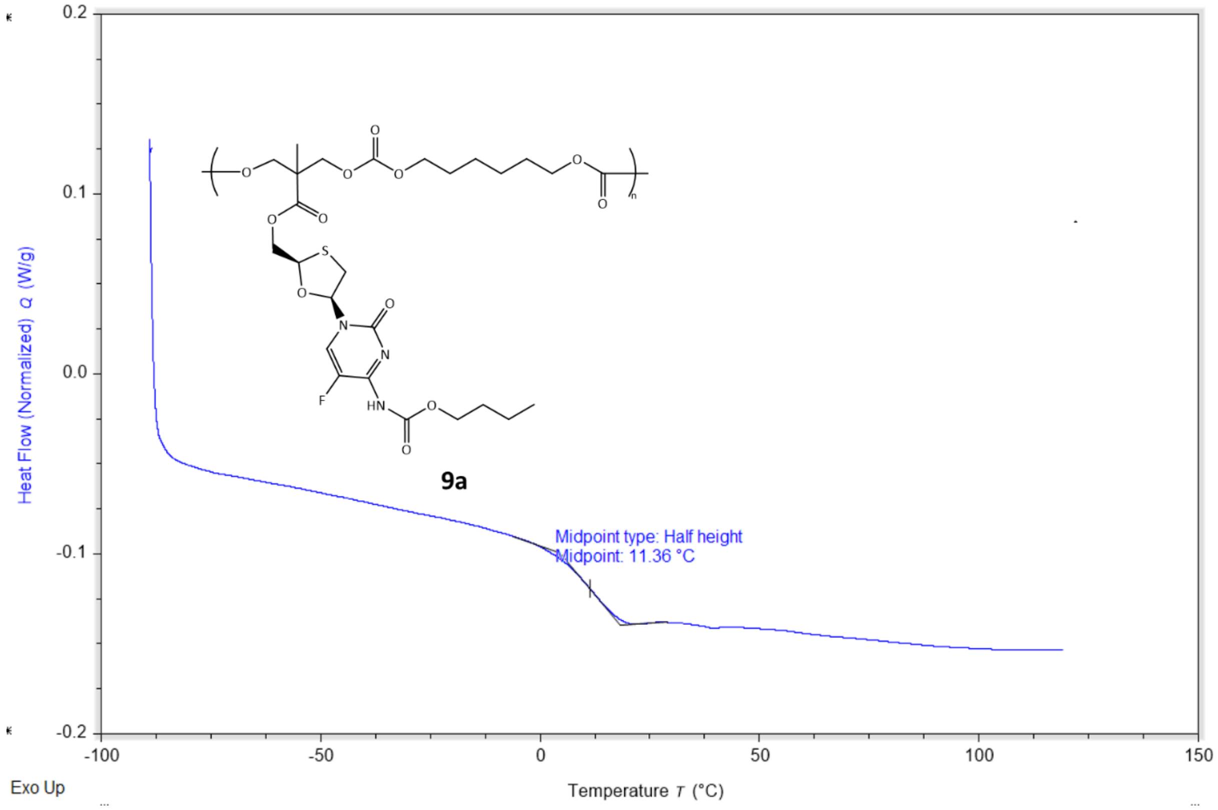

Figure S74: DSC chromatogram of polycarb (Hex-nBu) ***9a****.*

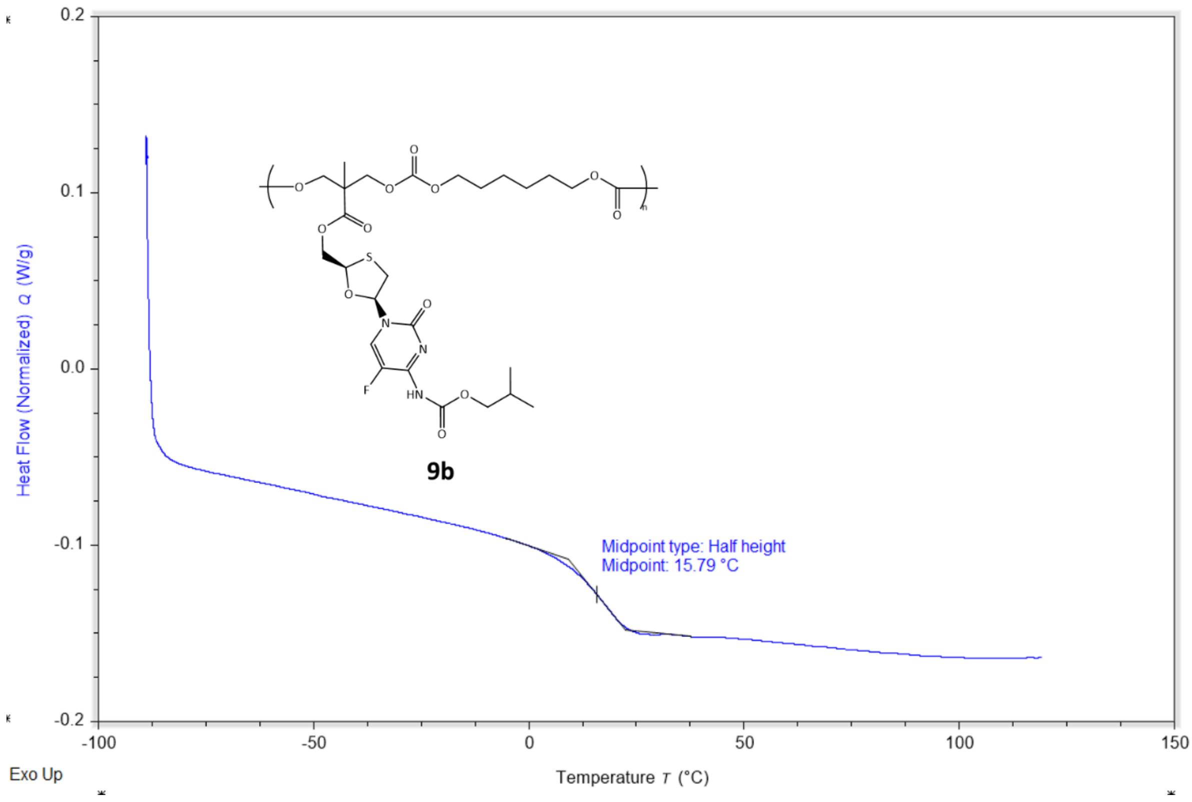

Figure S75: DSC chromatogram of polycarb (Hex-iBu) ***9b.***

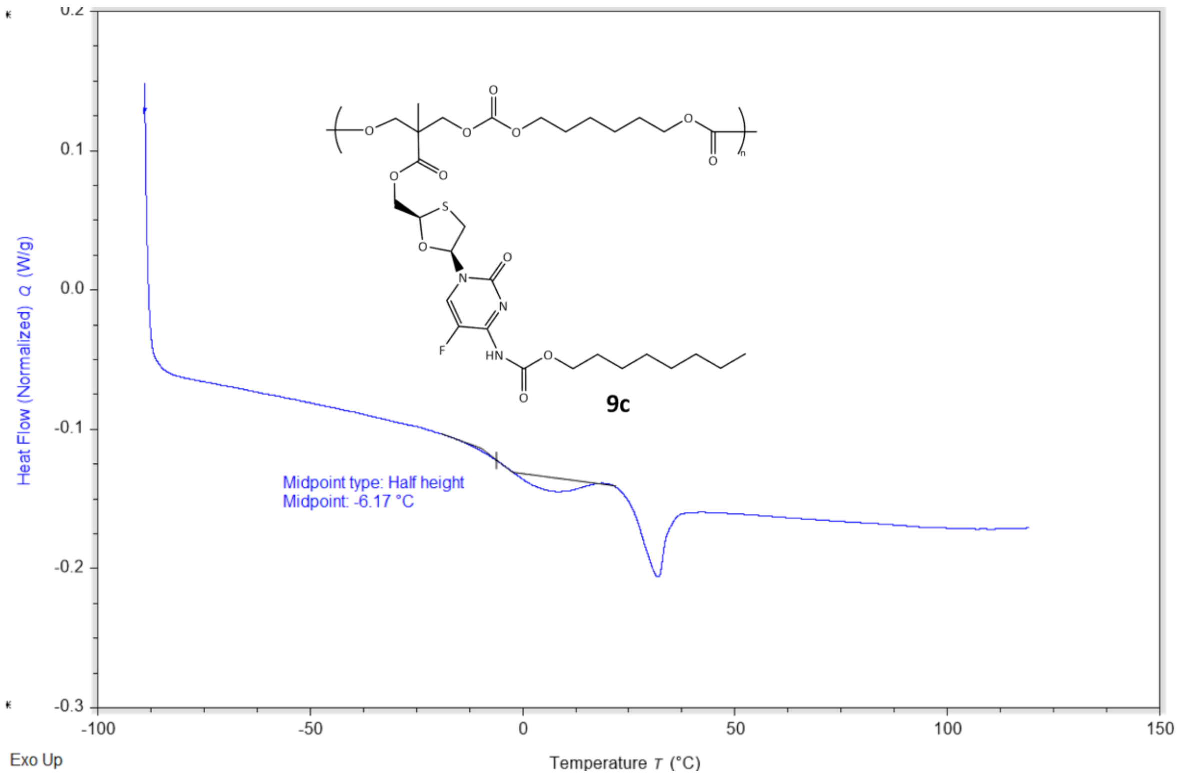

Figure S76: DSC chromatogram of polycarb (Hex-Oct) ***9c.***

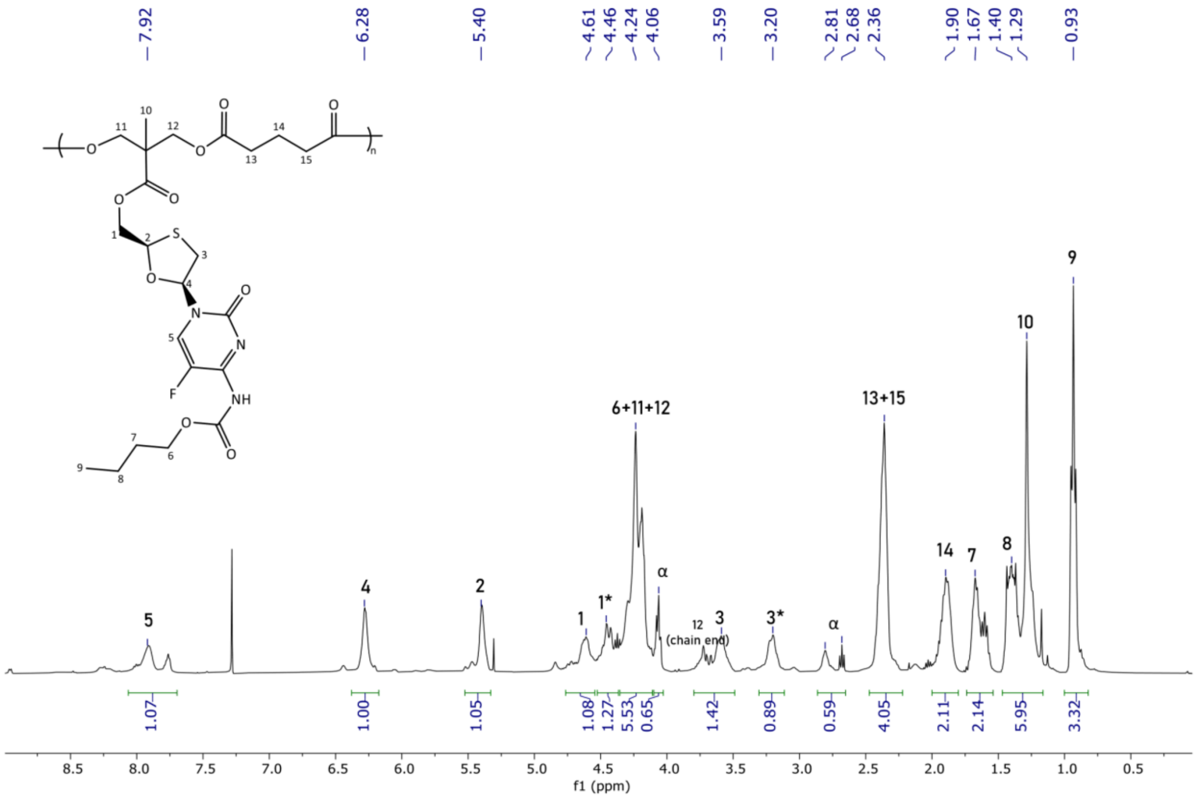

Figure S77: ^{1}H NMR ($CDCl_3$, 500 MHz) of polyester (Glu-nBu) ***10a.***

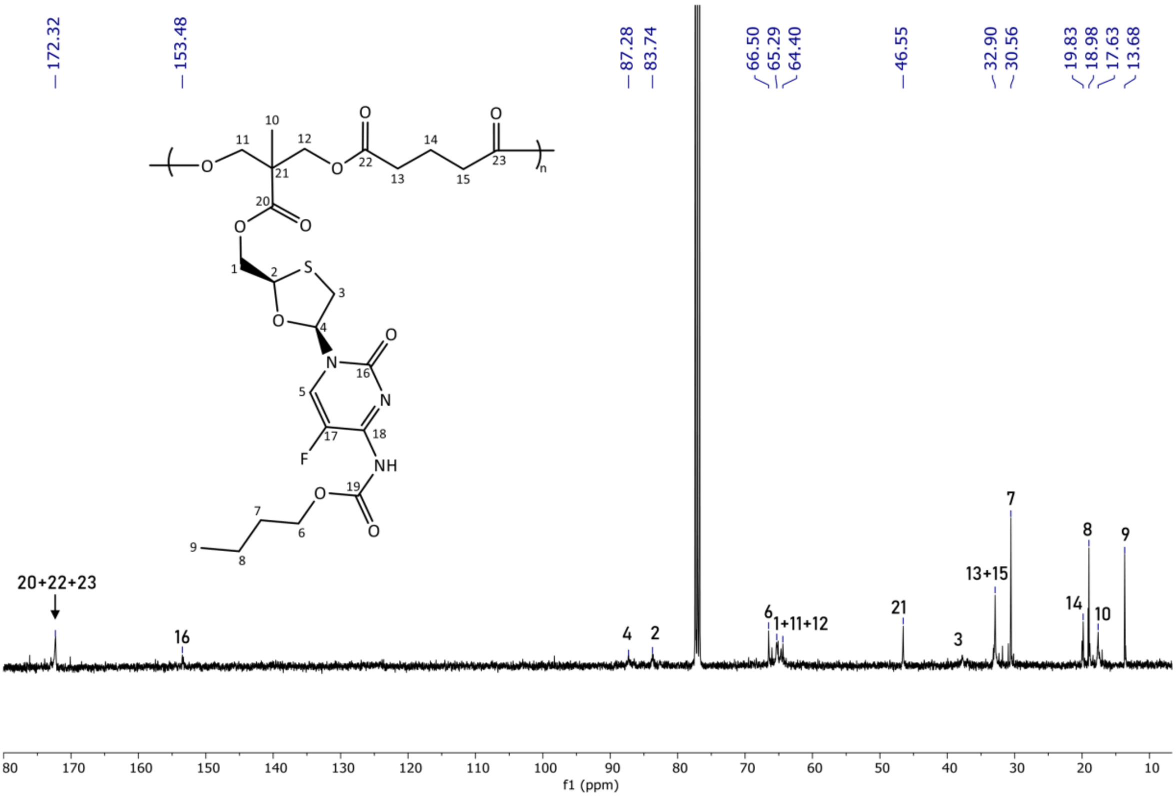

Figure S78: ^{13}C NMR ($CDCl_3$, 100 MHz) of polyester (Glu-nBu) ***10a.***

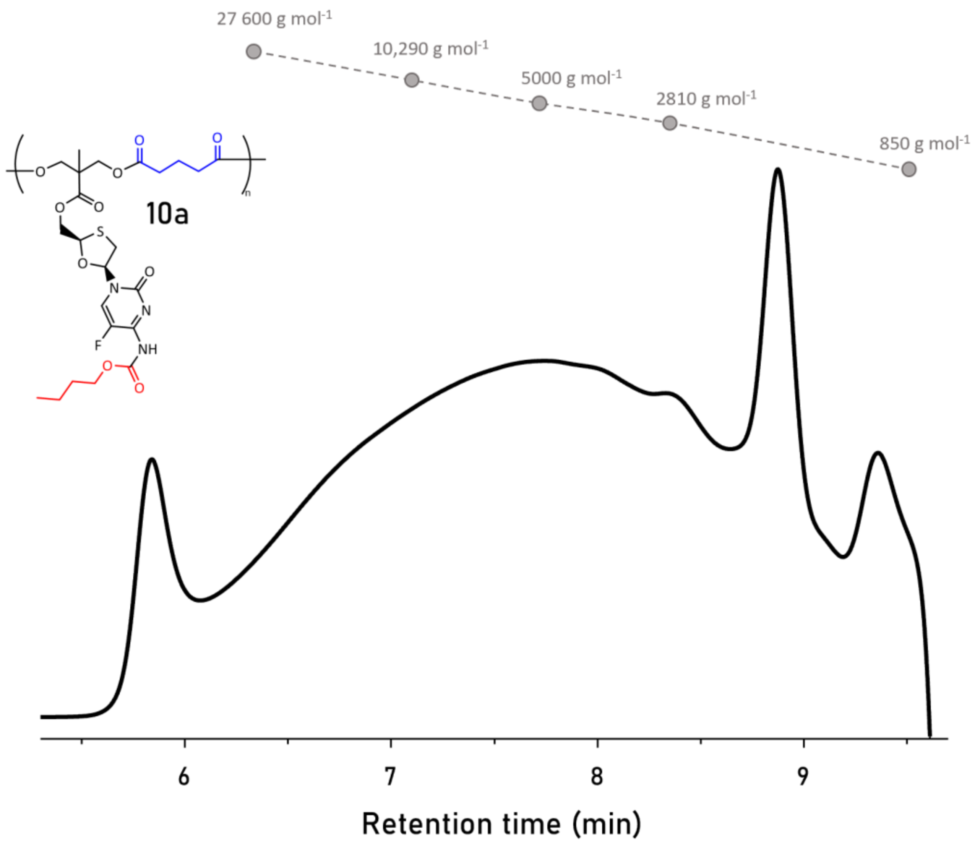

Figure S79: SEC chromatogram of polyester (Glu-nBu) ***10a****.*

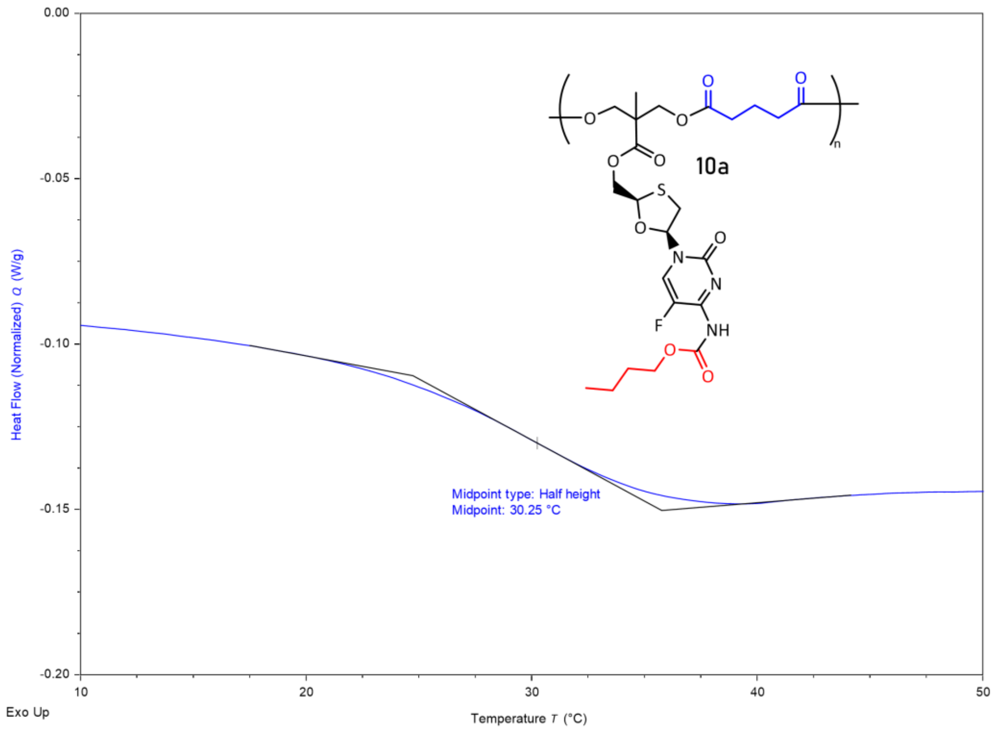

Figure S80: DSC chromatogram of polyester (Glu-nBu) ***10a****.*

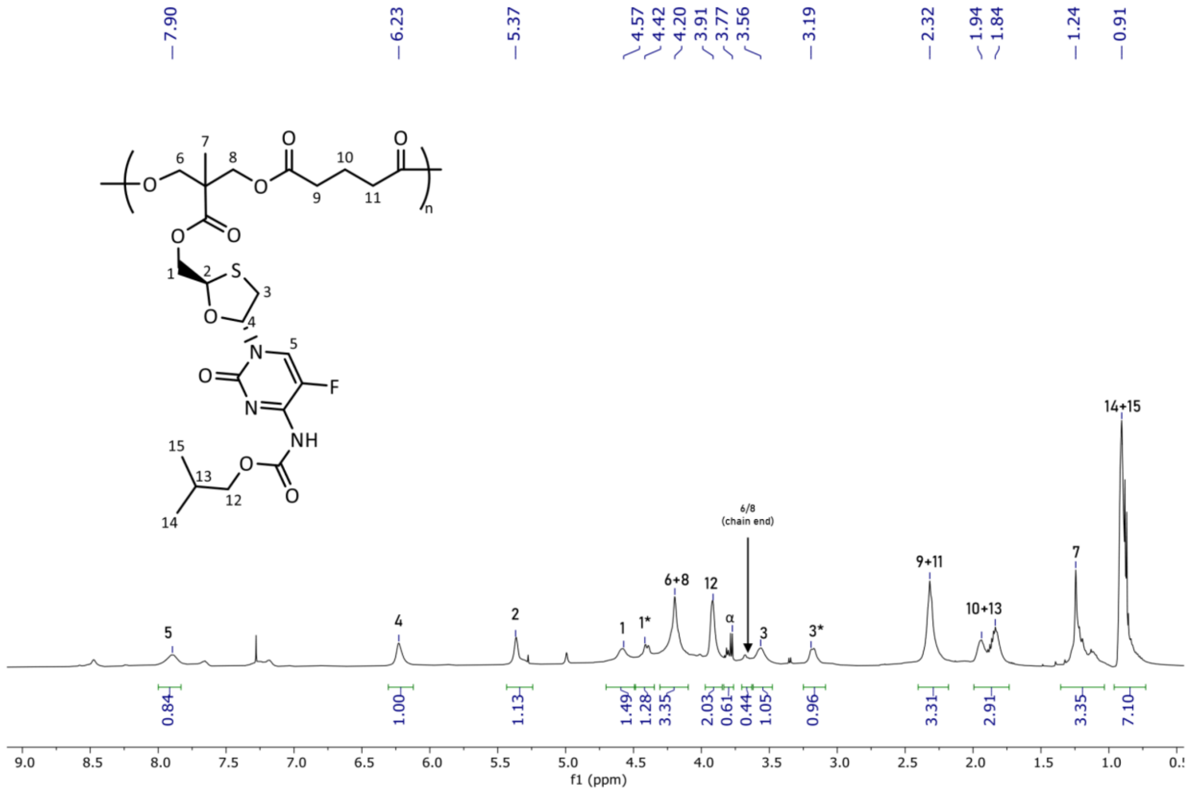

Figure S81: ^{1}H NMR ($CDCl_3$, 500 MHz) of polyester (Glu-iBu) ***10b****.*

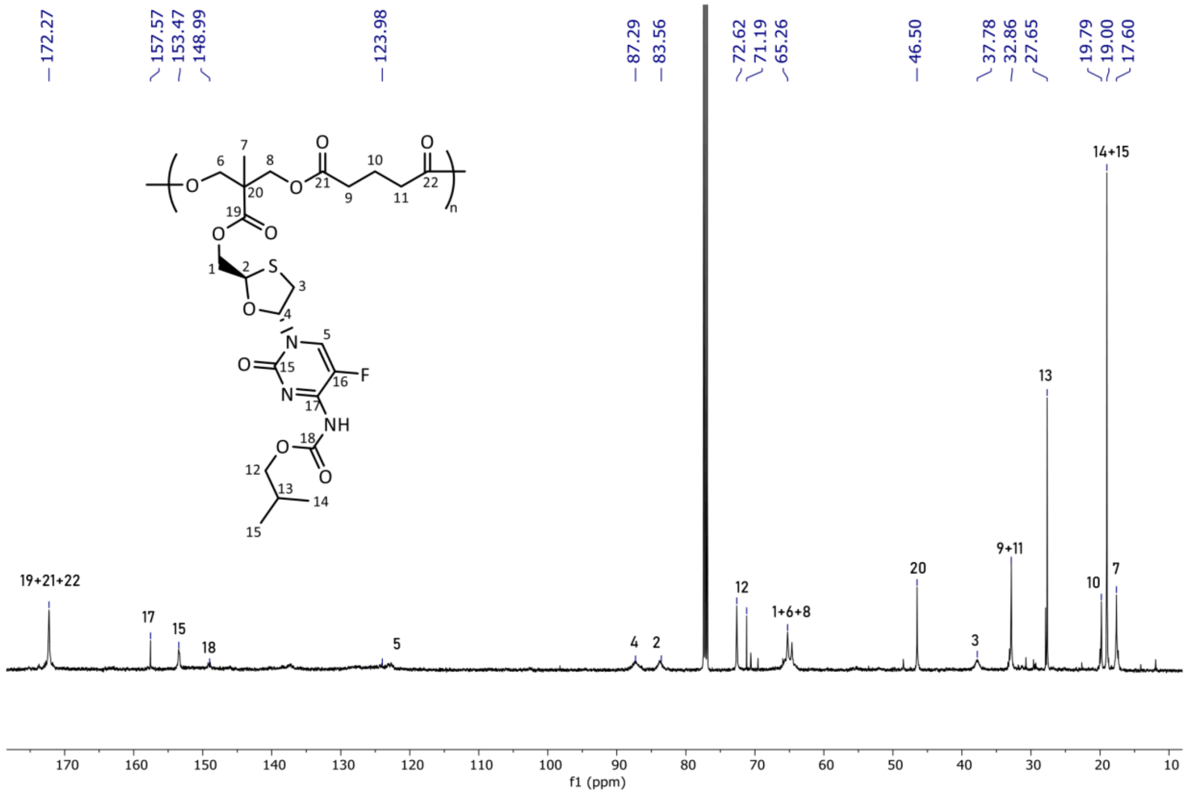

Figure S82: ^{13}C NMR ($CDCl_3$, 100 MHz) polyester (Glu-iBu) ***10b****.*

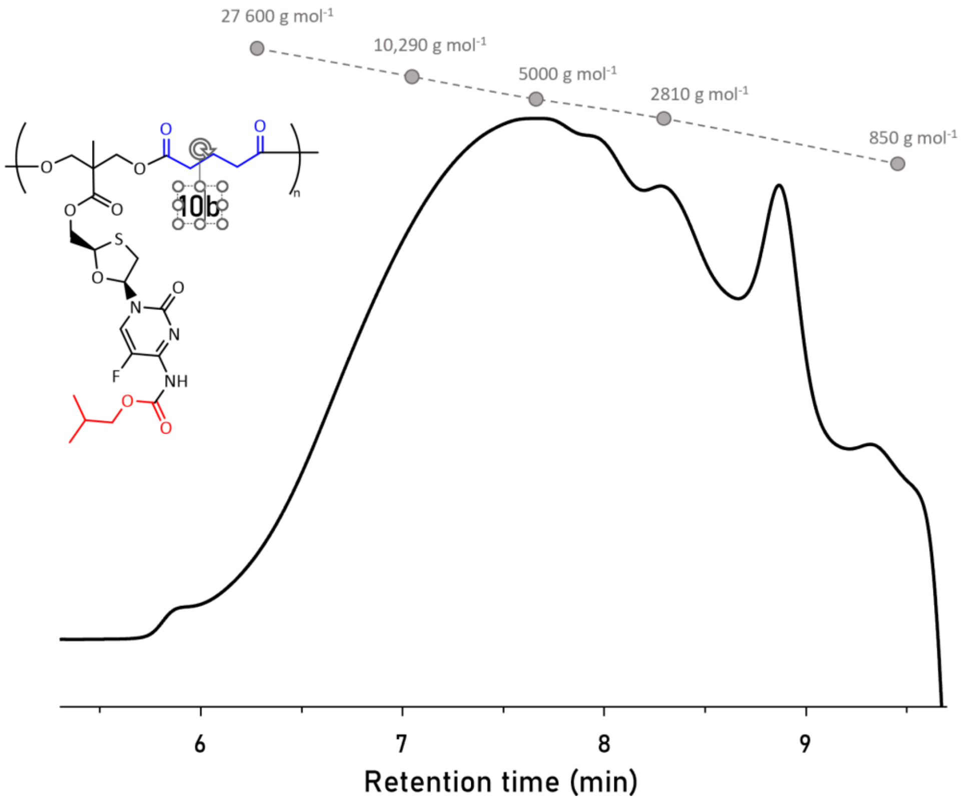

Figure S83: SEC chromatogram of polyester (Glu-iBu) ***10b****.*

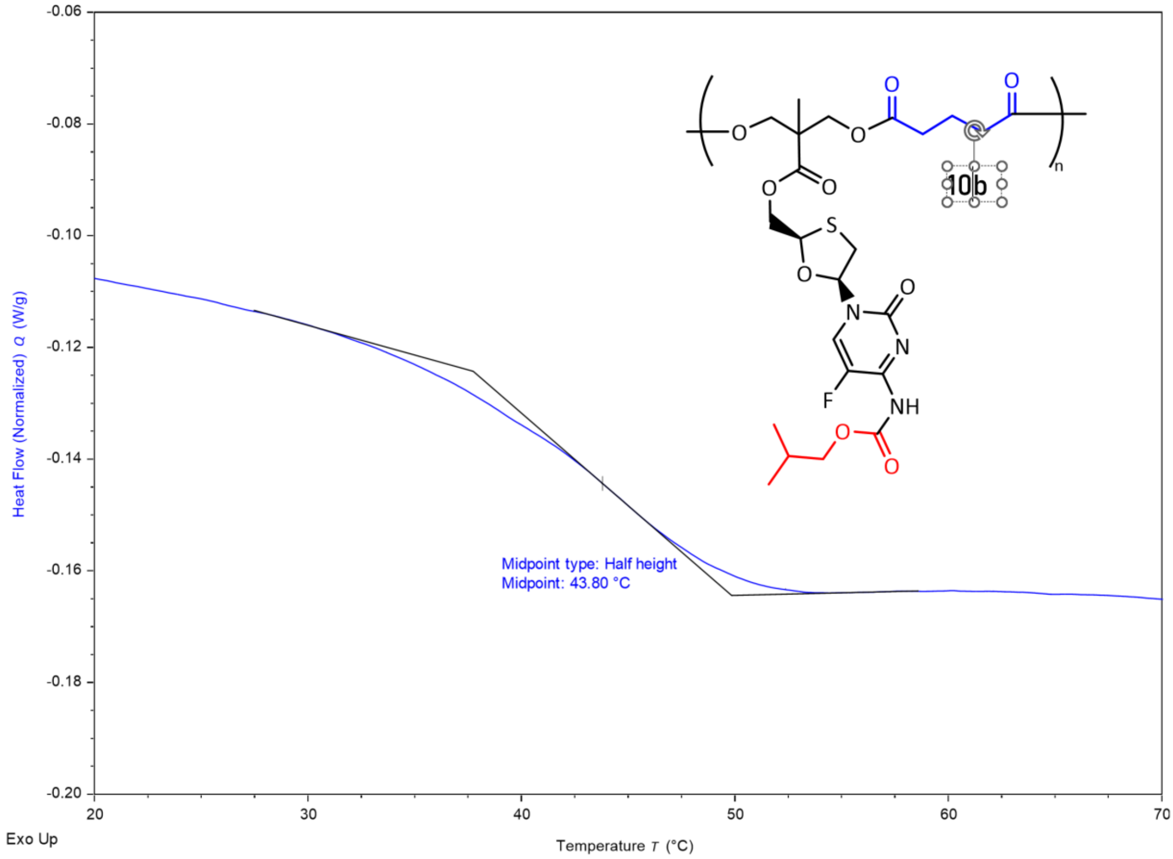

Figure S84: DSC chromatogram of polyester (Glu-iBu) ***10b****.*

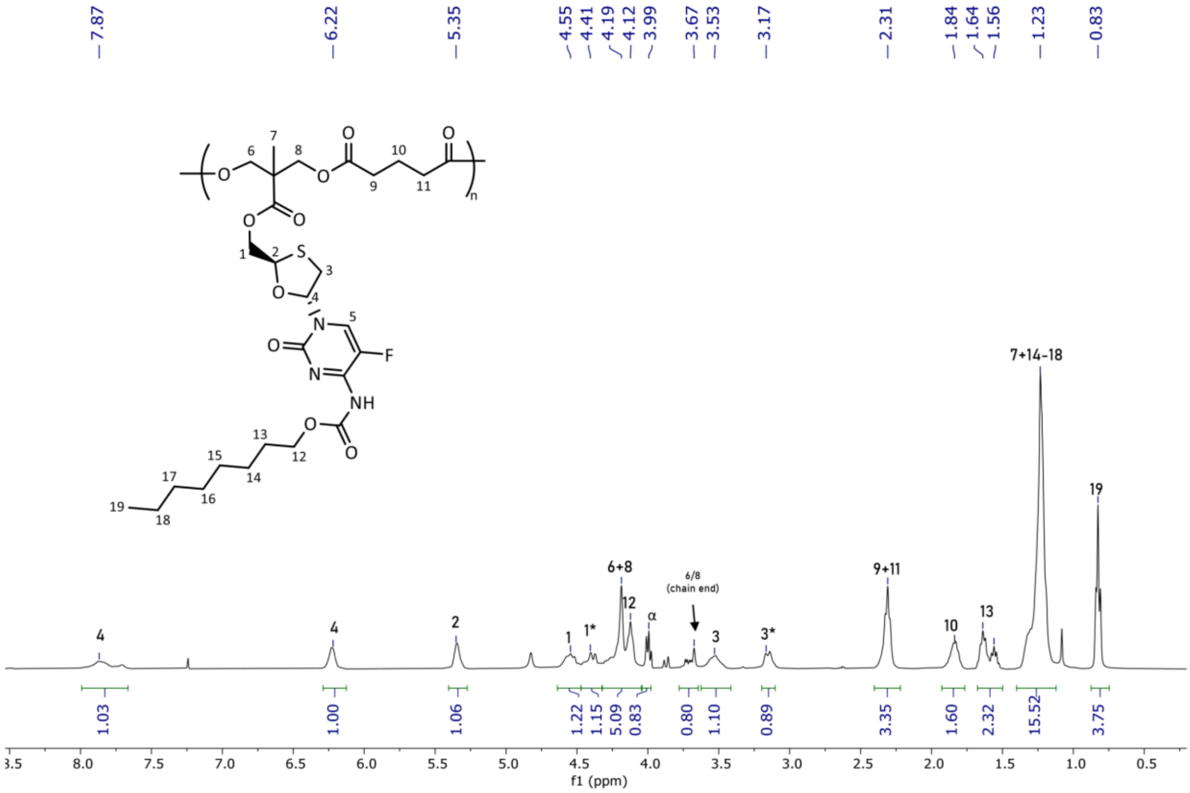

Figure S85: ^{1}H NMR (CDCl$_3$, 500 MHz) of polyester (Glu-Oct) ***10c****.*

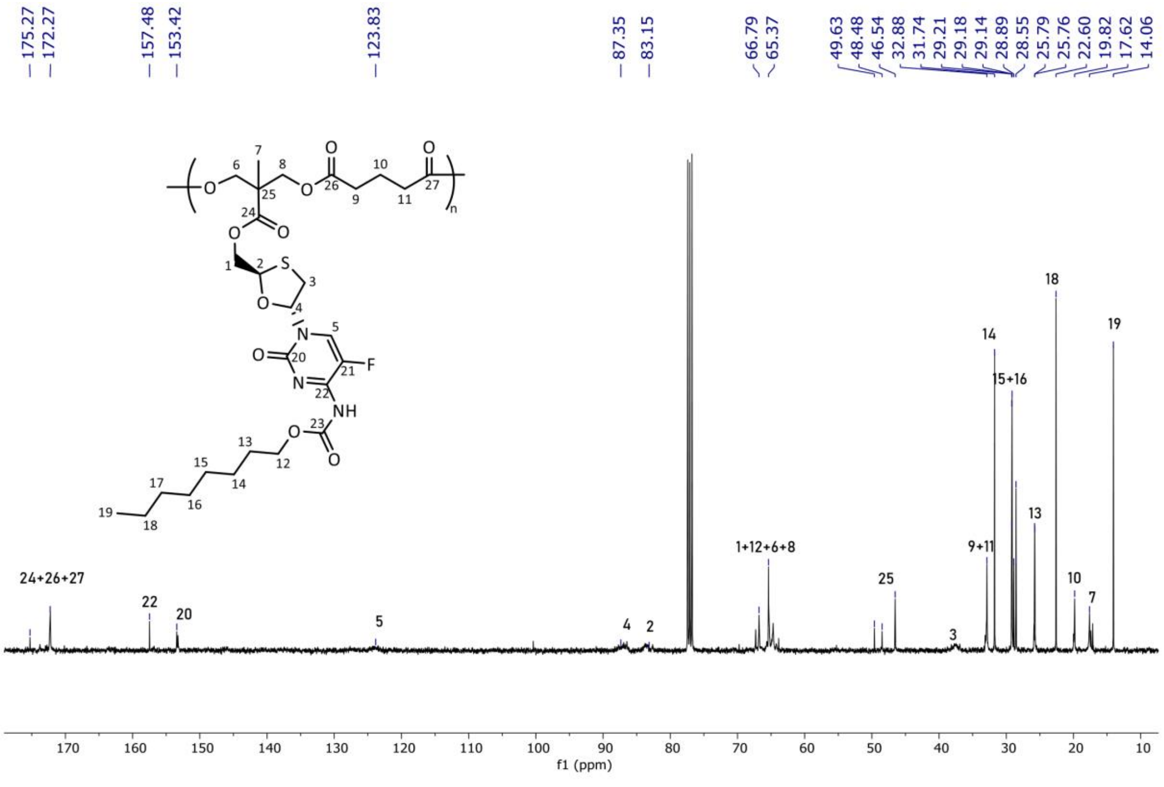

Figure S86: ^{13}C NMR (CDCl$_3$, 100 MHz) polyester (Glu-Oct) ***10c****.*

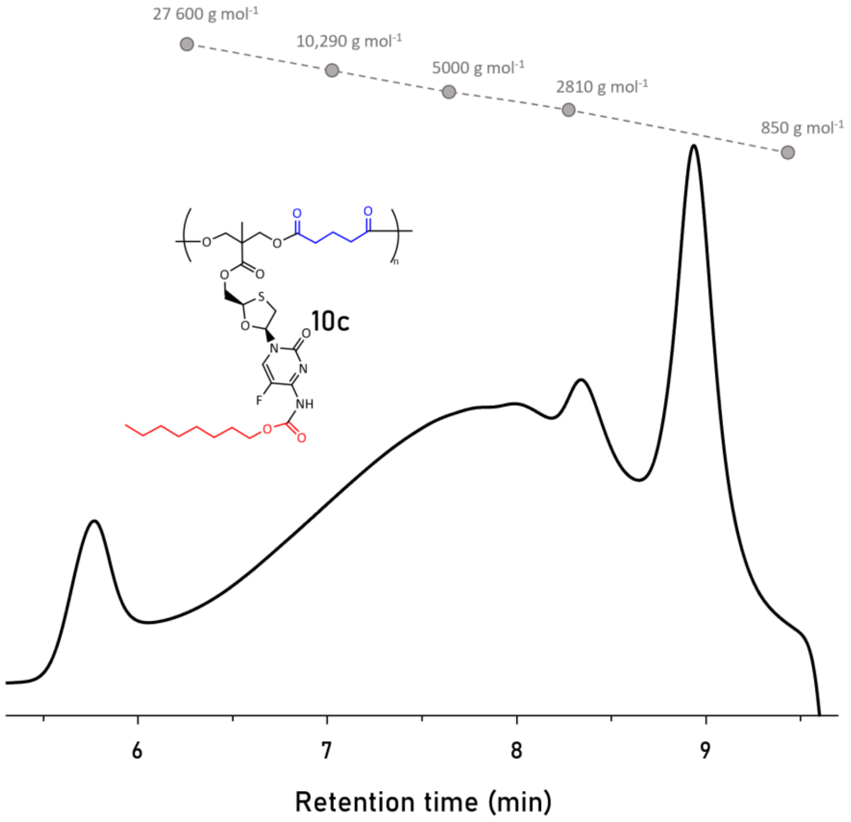

Figure S87: SEC chromatogram of polyester (Glu-Oct) ***10c****.*

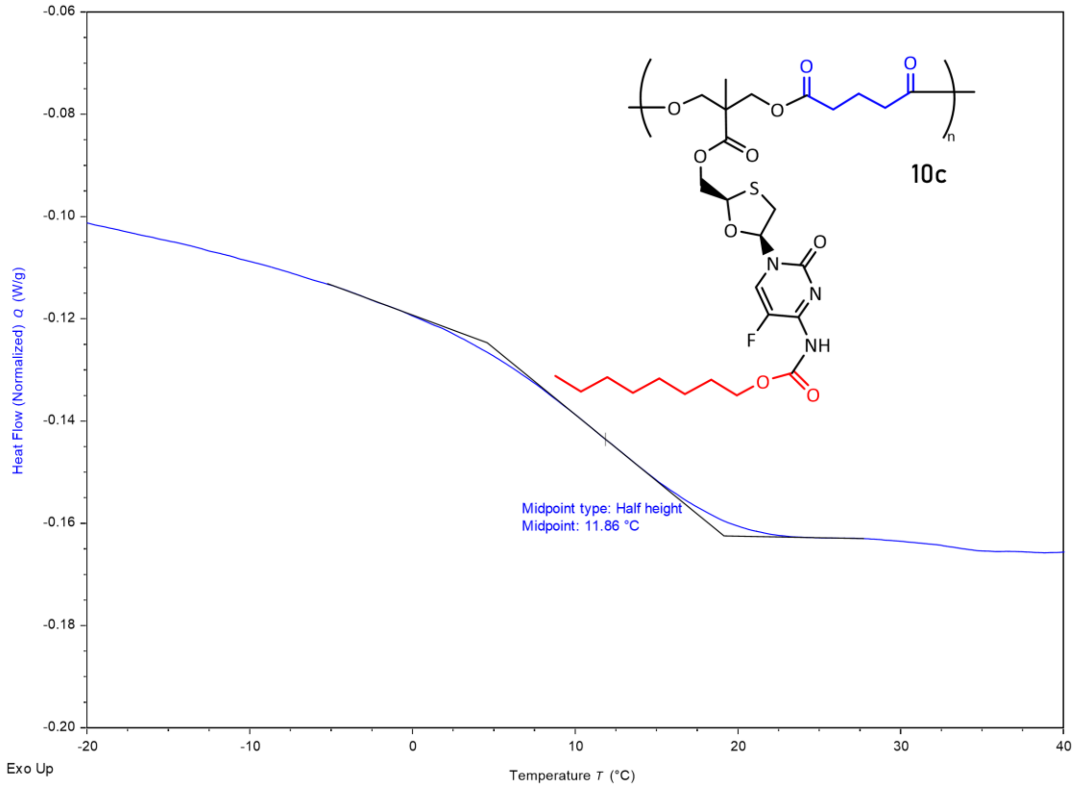

Figure S88: DSC chromatogram of polyester (Glu-Oct) ***10c.***

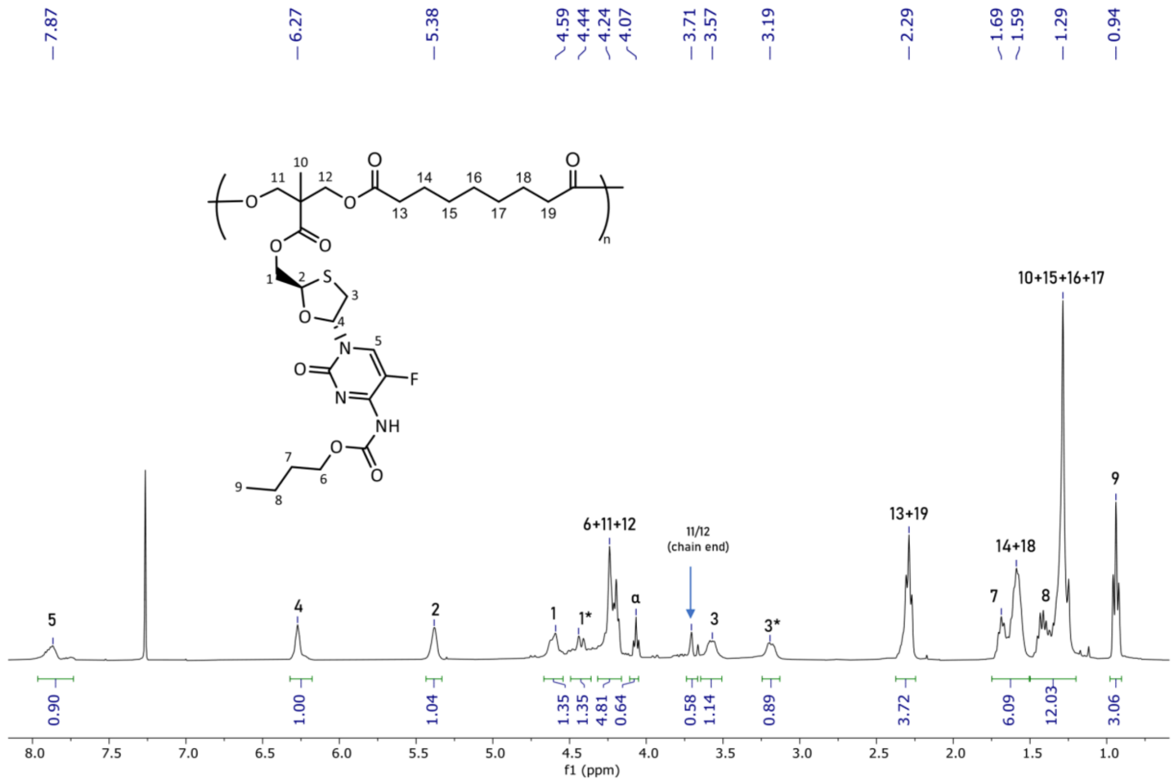

Figure S89: ^{1}H NMR ($CDCl_3$, 500 MHz) of polyester (Az-nBu) ***11a***.

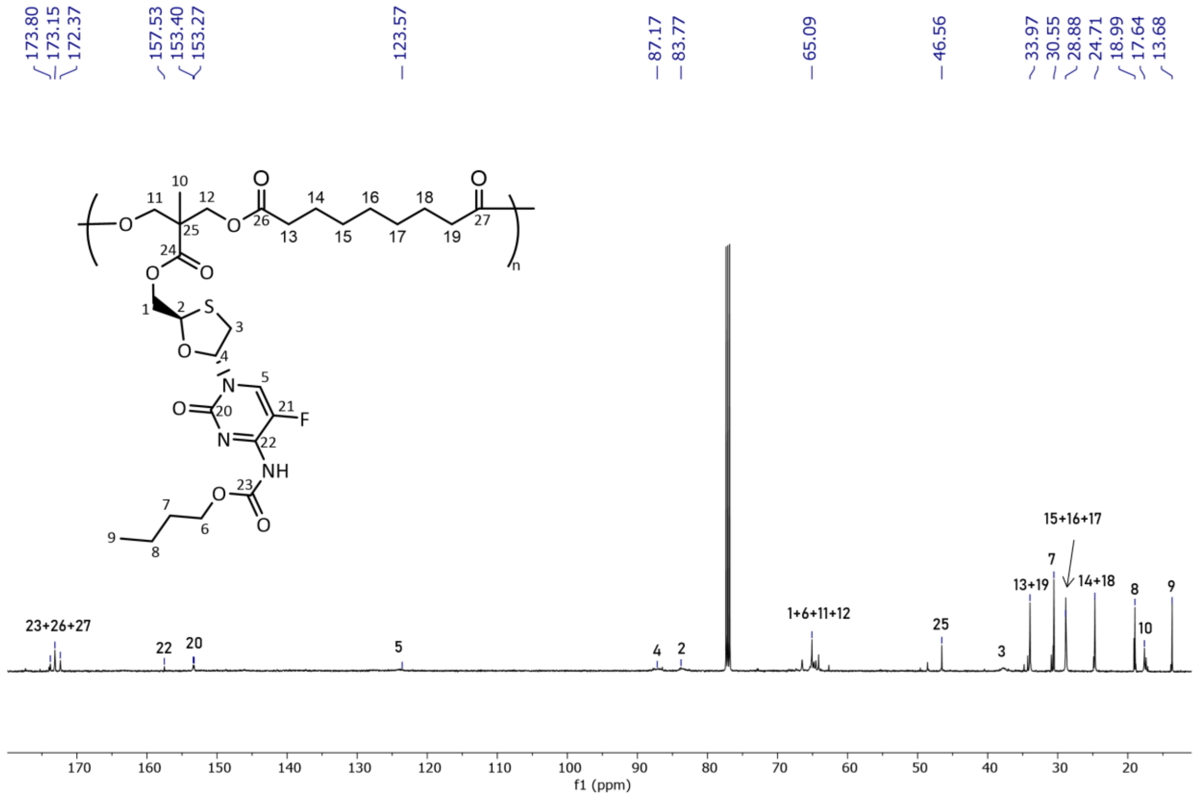

Figure S90: ^{13}C NMR ($CDCl_3$, 100 MHz) polyester (Az-nBu) ***11a***.

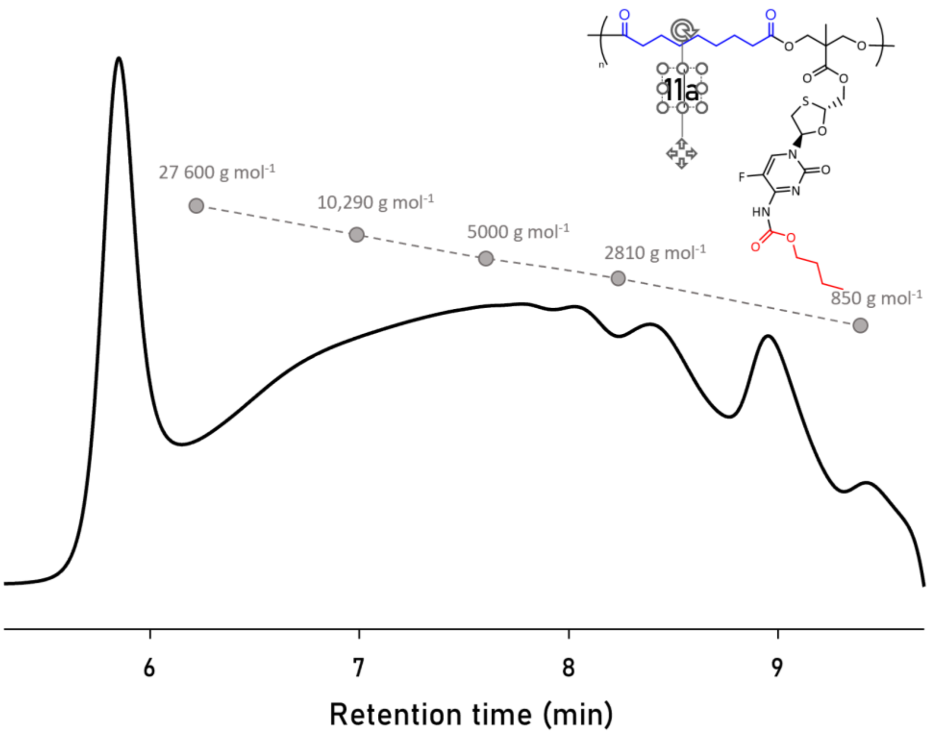

*Figure S91: SEC chromatogram of polyester (Az-nBu) **11a**.*

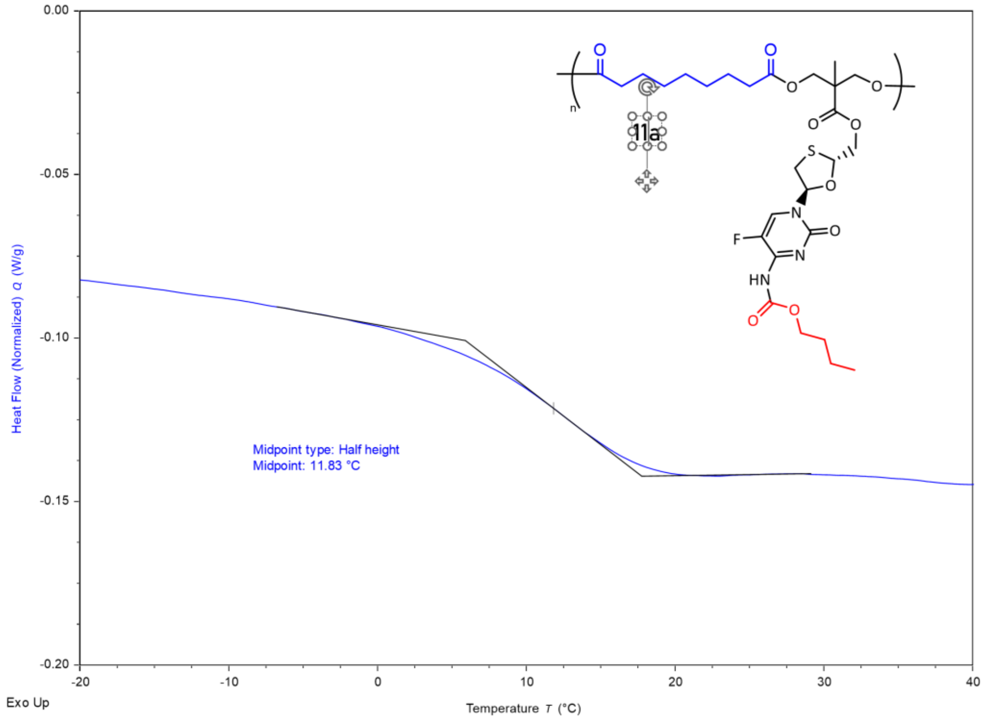

*Figure S92: DSC chromatogram of polyester (Az-nBu) **11a**.*

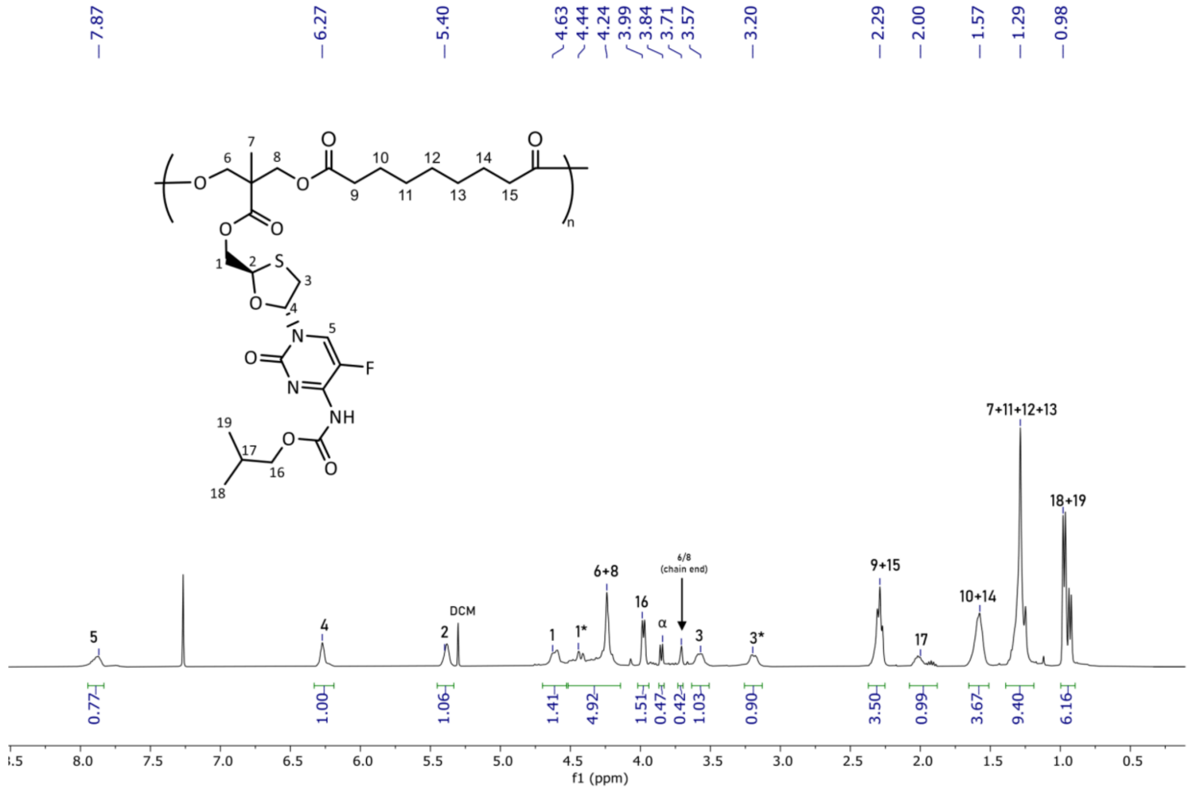

Figure S93: ^{1}H NMR ($CDCl_3$, 500 MHz) of polyester (Az-iBu) ***11b.***

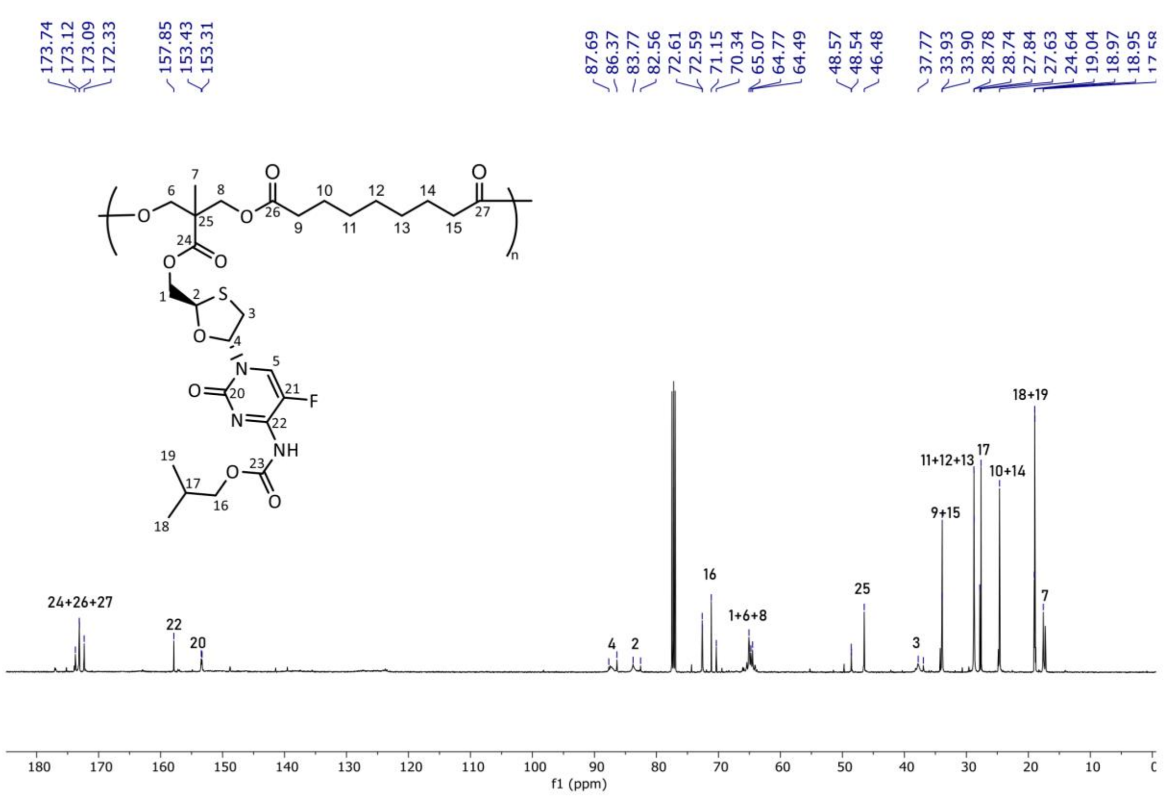

Figure S94: ^{13}C NMR ($CDCl_3$, 100 MHz) polyester (Az-iBu) ***11b.***

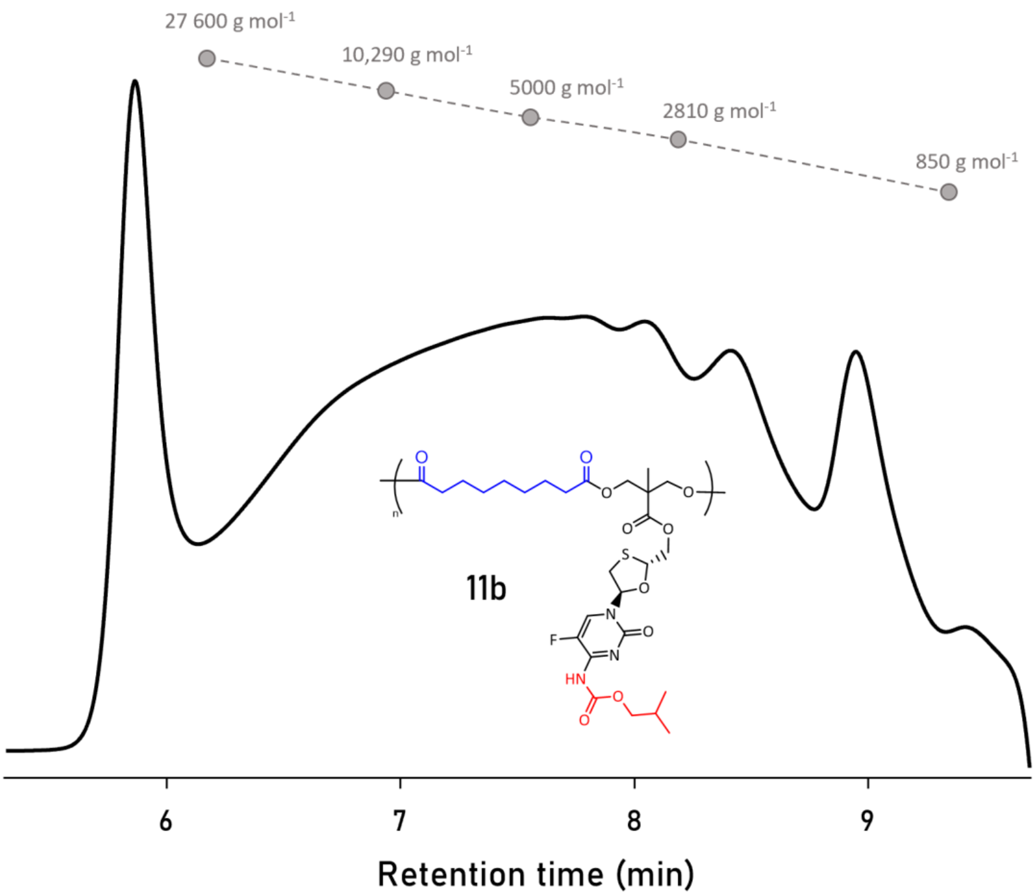

Figure S95: SEC chromatogram of polyester (Az-iBu) ***11b.***

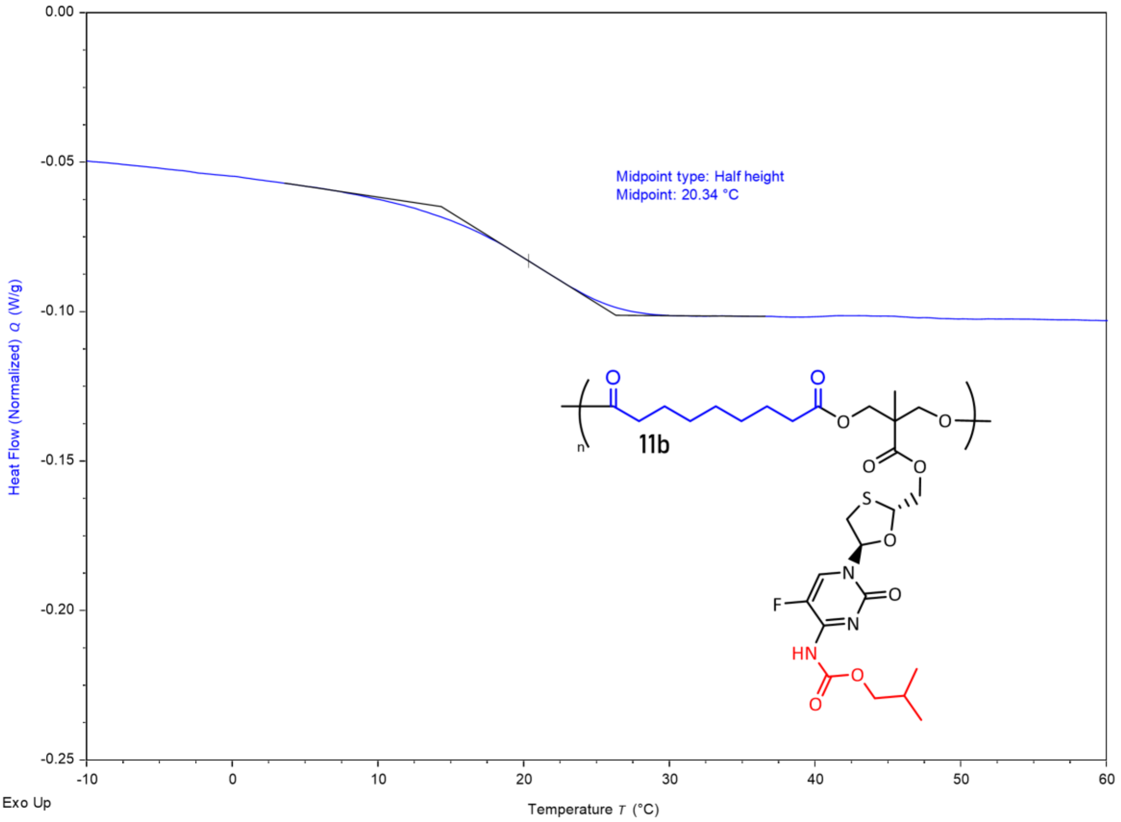

Figure S96: DSC chromatogram of polyester (Az-iBu) ***11b****.*

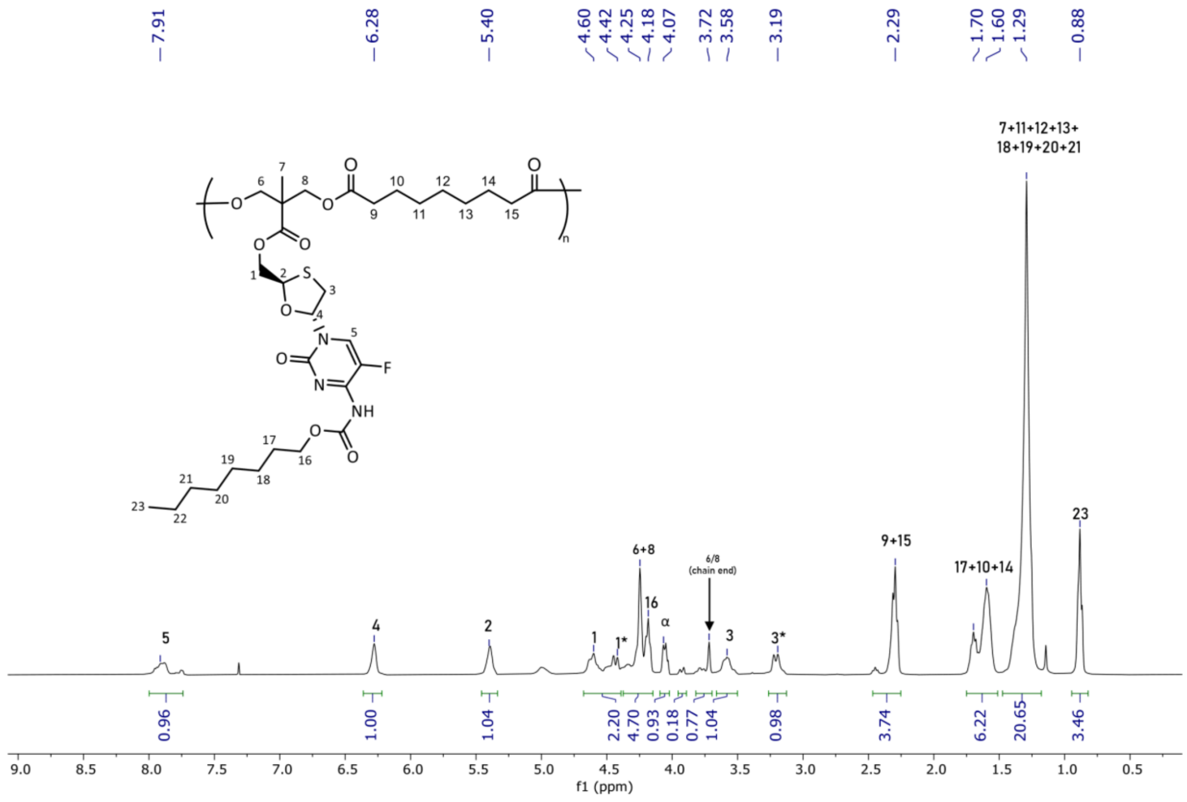

*Figure S97: ^{1}H NMR ($CDCl_3$, 500 MHz) of polyester (Az-Oct) **11c**.*

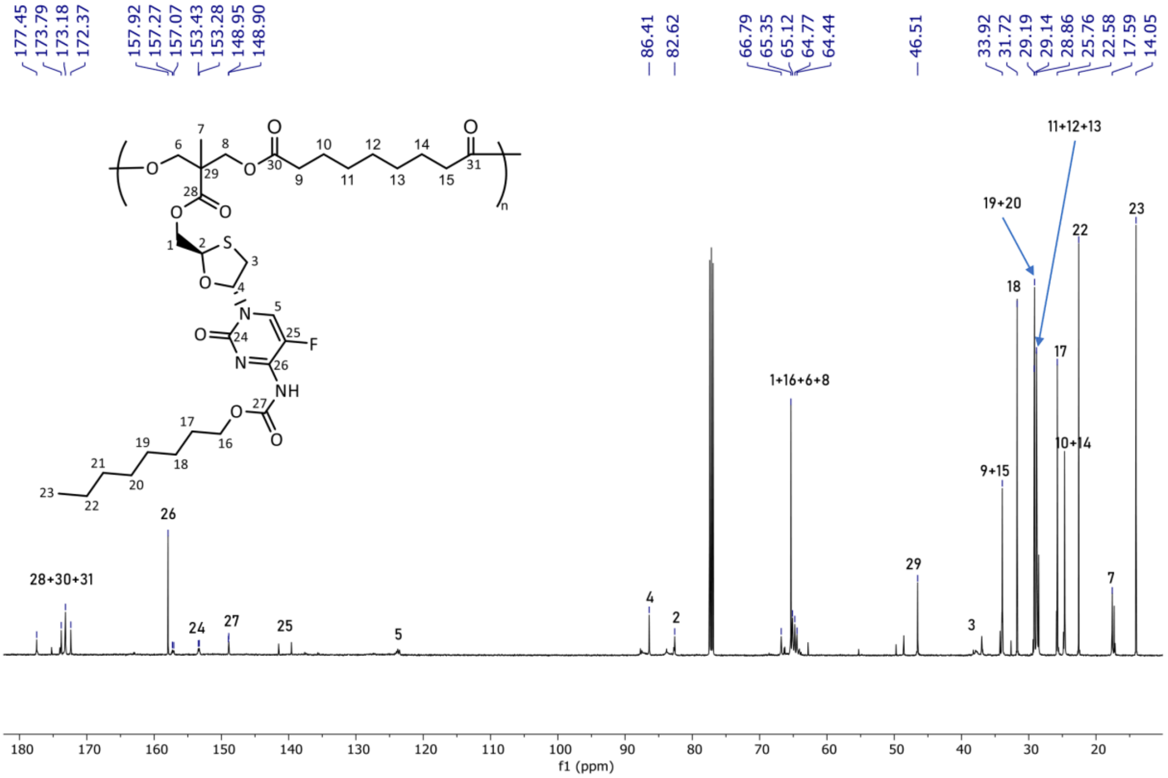

*Figure S98: ^{13}C NMR ($CDCl_3$, 100 MHz) polyester (Az-Oct) **11c**.*

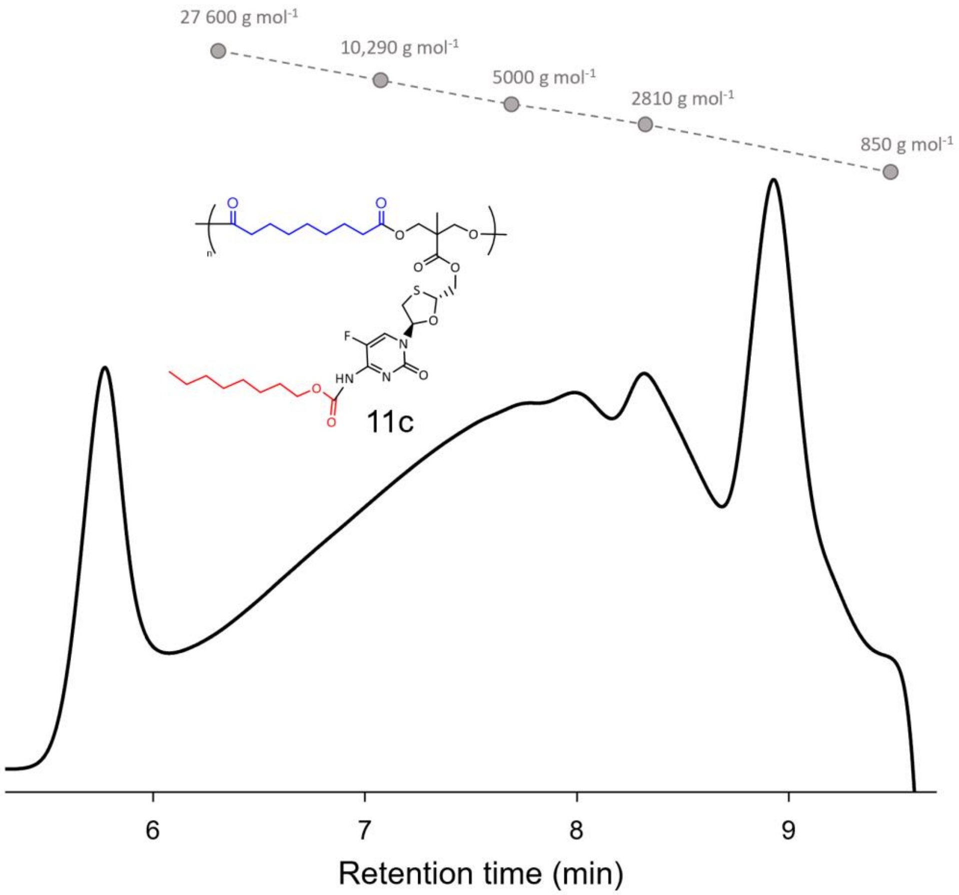

Figure S99: SEC chromatogram of polyester (Az-Oct) ***11c****.*

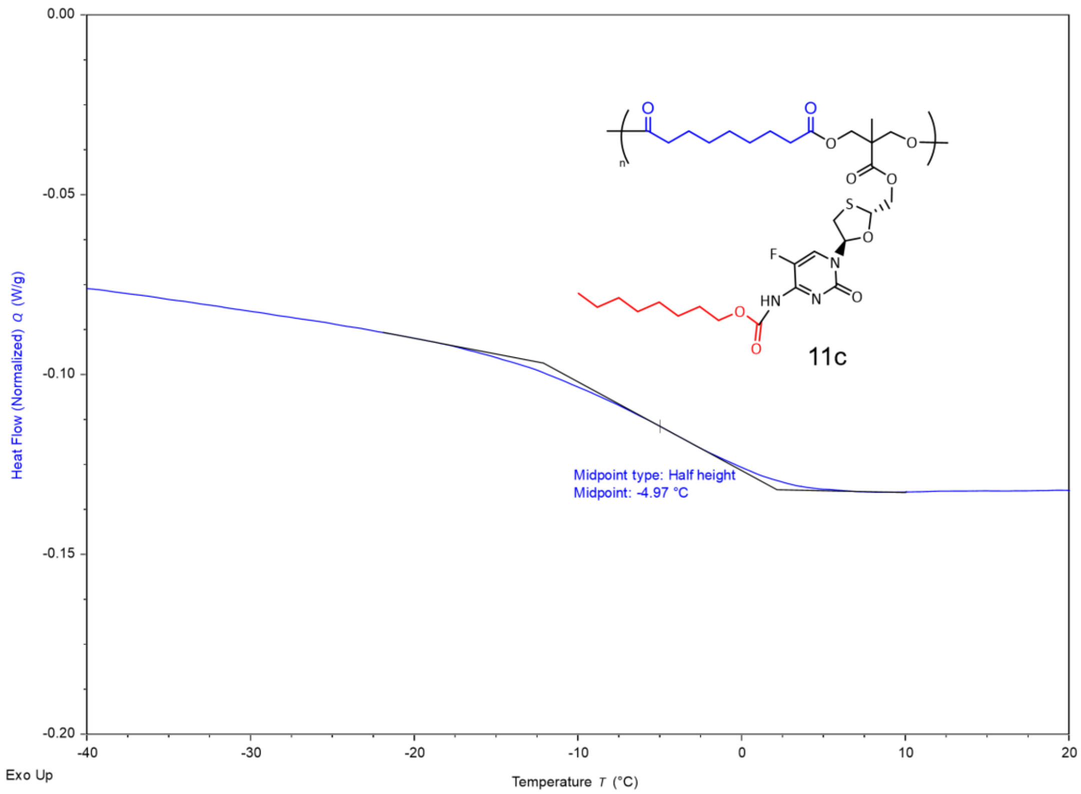

Figure S100: DSC chromatogram of polyester (Az-Oct) ***11c****.*

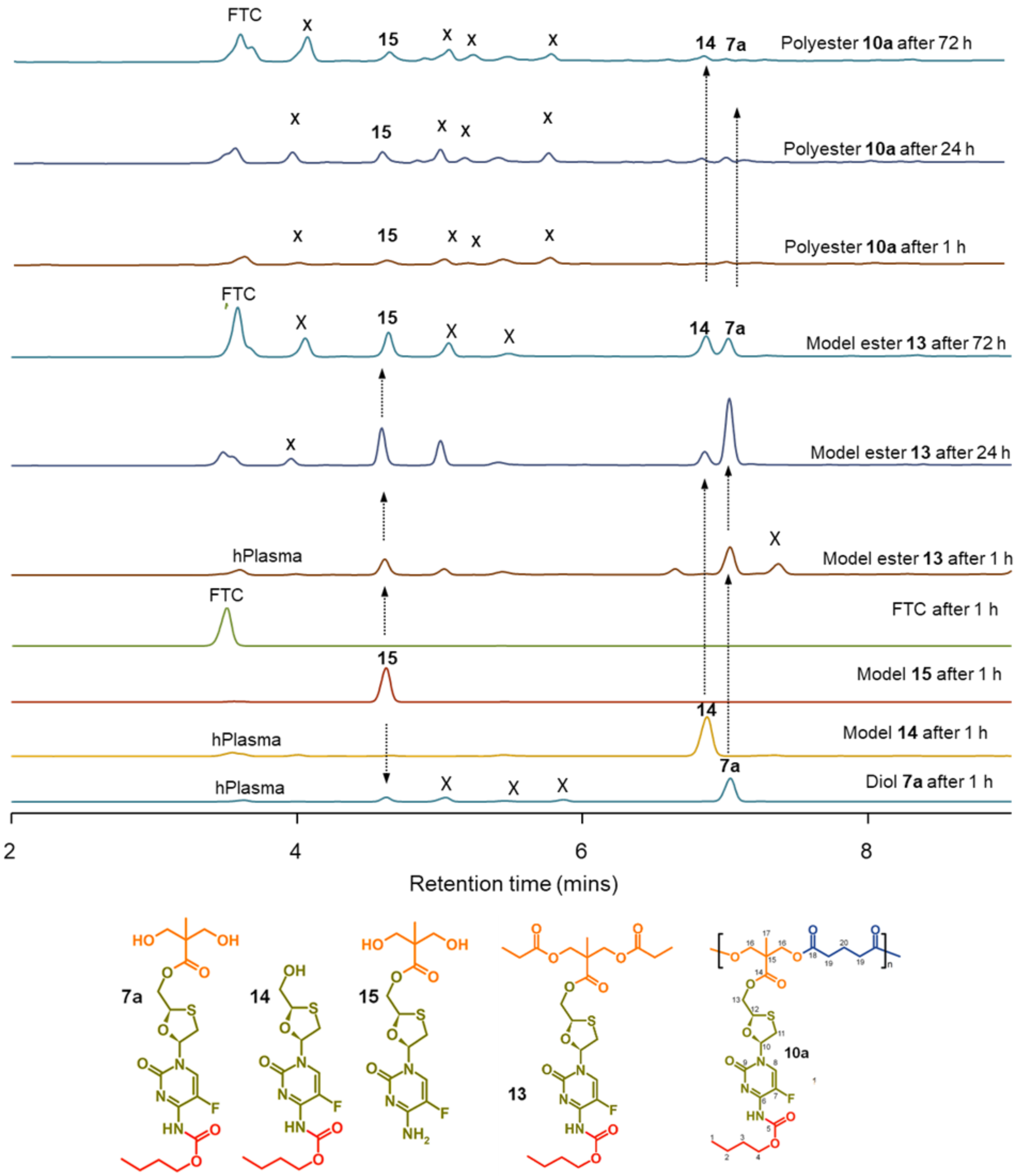

Figure S101: Overlaid HPLC chromatogram (UV detector at λ = 280 nm) of polyester(Glu-nBu) ***10a****, showing evolution of degradation products in gendered hPlasma over 72 hours.*

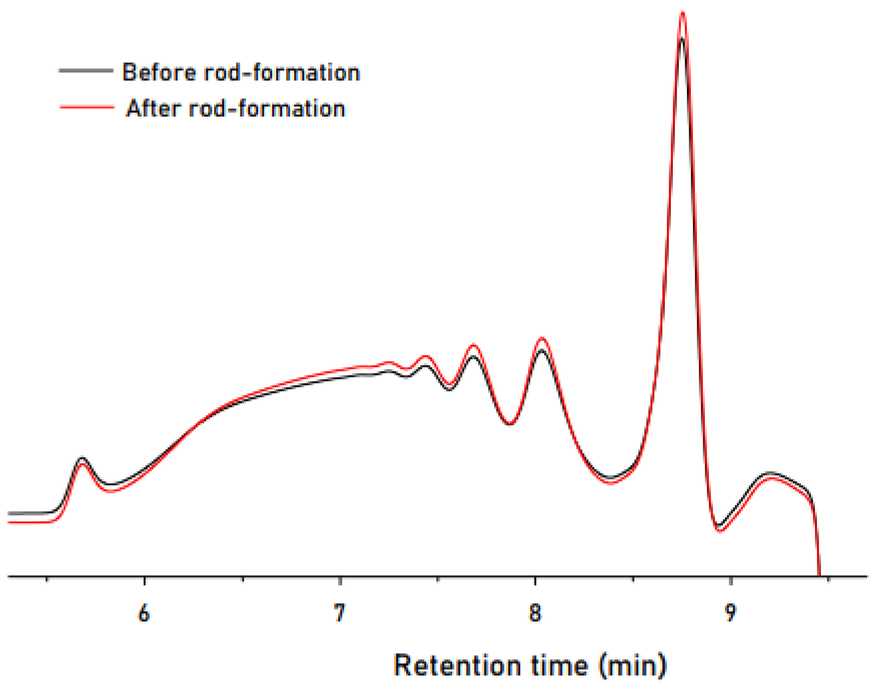

*Figure S102: SEC chromatogram of polycarb(Et-nBu) **8a** before and after rod-formation.*

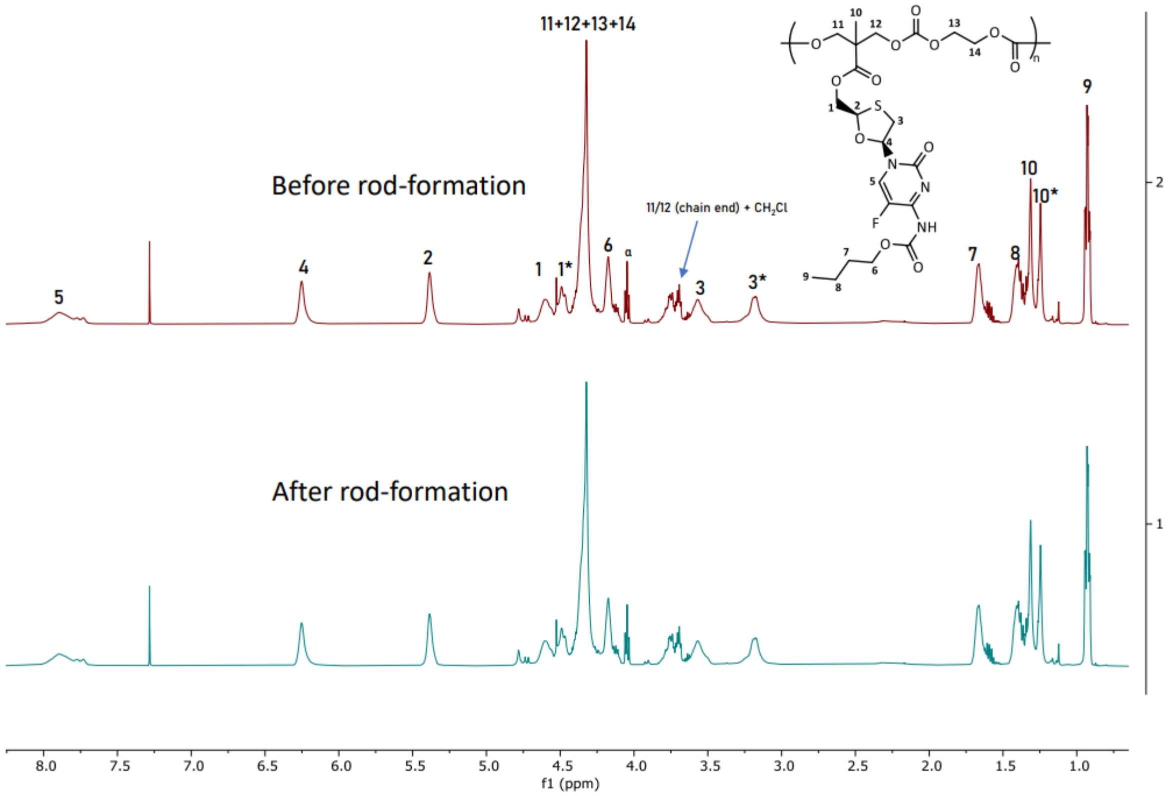

*Figure S103: ^{1}H NMR ($CDCl_3$, 500 MHz) of polycarb(Et-nBu) **8a** before and after rod-formation.*

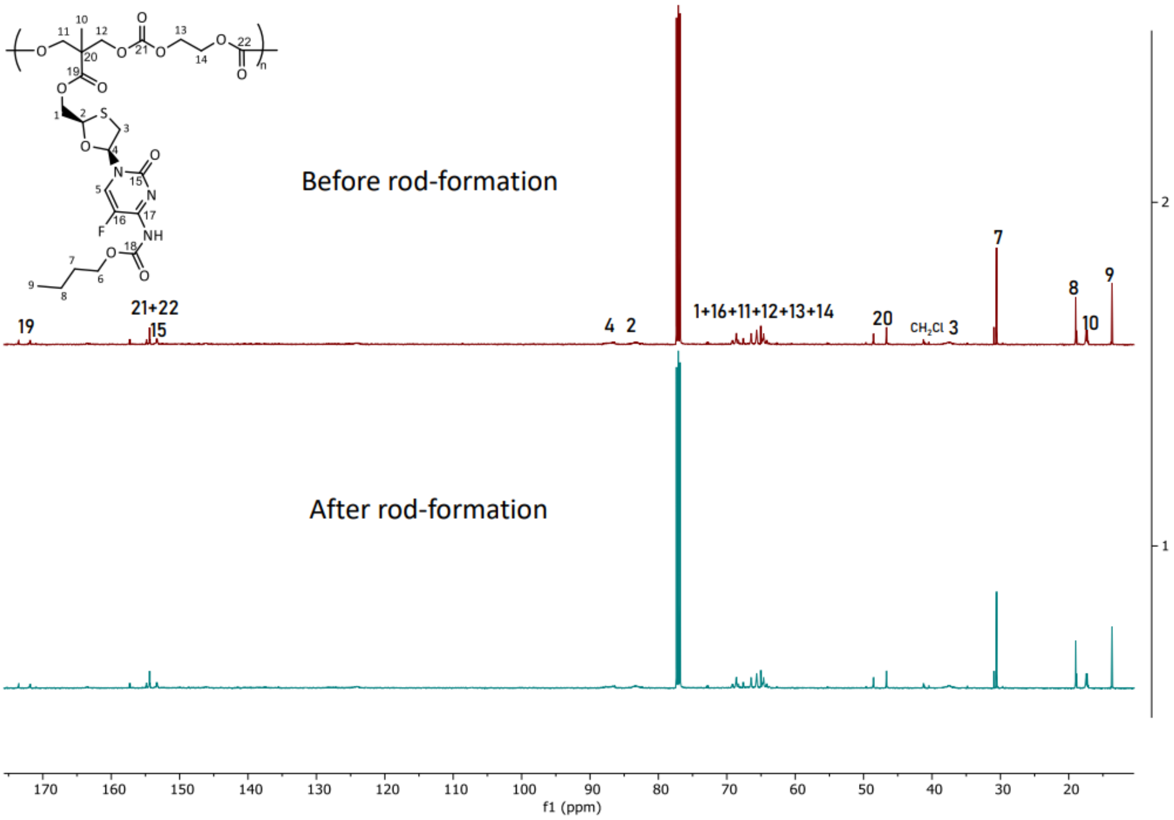

Figure S104: ^{13}C NMR ($CDCl_3$, 100 MHz) of polycarb(Et-nBu) ***8a*** *before and after rod-formation.*
